# Homoconjugation-induced enhancements of photophysical properties in donor–acceptor triptycenes arise from interplay between intramolecular charge transfer and exciton states

**DOI:** 10.1039/d6sc01073c

**Published:** 2026-06-08

**Authors:** Stefan Warrington, Hristo Ivov Gonev, Gary S. Nichol, Eleanor M. Dodd, Simon J. Coles, Thomas J. Penfold, Marc K. Etherington, Tracey M. Clarke, Iain A. Wright

**Affiliations:** a EaStCHEM School of Chemistry, University of Edinburgh Joseph Black Building, David Brewster Road Edinburgh EH9 3FJ UK iain.wright@ed.ac.uk; b Department of Chemistry, Loughborough University Epinal Way Loughborough Leicestershire LE11 3TU UK; c Department of Chemistry, University College London Christopher Ingold Building London WC1H 0AJ UK tracey.clarke@ucl.ac.uk; d EPSRC Crystallographic Service, Department of Chemistry, University of Southampton Highfield Southampton SO17 1BJ UK; e Chemistry, School of Natural and Environmental Sciences, Newcastle University Newcastle upon Tyne NE1 7RU UK; f School of Engineering, Physics and Mathematics, Northumbria University Ellison Place Newcastle upon Tyne NE1 8ST UK

## Abstract

Strategies for tuning the optical properties of organic chromophores generally focus on shifting the edges of the spectrum: this might be red-shifting the longest absorbance band to improve solar absorbance, or blue-shifting of the highest energy emission band towards deep blue emission. In contrast, strategies to enhance molar absorptivity and control excited state rate constants are less obvious, with intermolecular excitons such as *J*-aggregates providing arguably the most powerful approach. Here, a homologous series of π-extended triptycenes is presented which reveal opportunities to control both aspects. These molecules have electronic spectra consisting of two distinct regimes, a low energy intramolecular charge transfer and a mid-spectral progression which has characteristics similar to that of a *J*-aggregate in several respects. This reveals that a homoconjugated framework can be utilised rationally to separate and independently control distinct regions of the electronic structure of the molecule, here leading to controllably amplified mid-spectrum absorbance intensities and high fluorescence quantum yields.

## Abstract 2

Interplay between distinct intramolecular exciton and charge transfer states in all-ring-fused triptycene chromophores leads to high photoluminescence quantum yields, and provides a general explanation for LUMO-homoconjugation induced enhancements.

## Graphical abstract

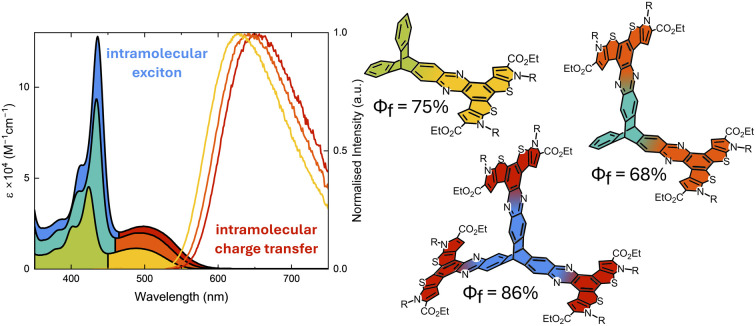

## Introduction

Covalently-linked chromophore assemblies with three-dimensional (3D) branched topologies are a promising class of molecular materials for organic energy conversion technologies.^1^ This is especially apparent in the optoelectronic properties of systems where the chromophores are held in sufficiently close proximity to facilitate interchromophore interactions.^2–9^ Benefits of such architectures may include improved photoabsorbance cross-sections, thin film morphologies and interfacing which can improve performance in organic photovoltaics (OPVs)^10–16^ or exert control over the excited state behaviour of emitters for organic light-emitting devices (OLEDs)^17–23^ and related technologies.

The trefoil molecule triptycene has a robust 3D carbon framework from which to construct such systems by appending chromophores onto the three fins. It also provides a distinctive route to encourage electronic communication between the appended chromophores through homoconjugation.^24^ Homoconjugation is the through-space orbital overlap of two π-systems separated by a single non-conjugated atom or group, in iptycenes these are the bridgehead sp^3^ carbon atoms.^25,26^

In triptycene-based intramolecular charge transfer (ICT) emitters, positive influences on photophysical properties have been observed. These include very large enhancements in molar absorptivities (*ε*) and oscillator strengths (*f*_osc_), higher photoluminescence quantum yields (*Φ*) and acceleration of rate constants facilitated by increased spin–orbit coupling (SOC). Observations made of results to date suggest that if large positive influences are to be observed then it is imperative that the lowest unoccupied molecular orbital (LUMO) be localised over the homoconjugated iptycene core.^20,24,27^

**Chart 1:**
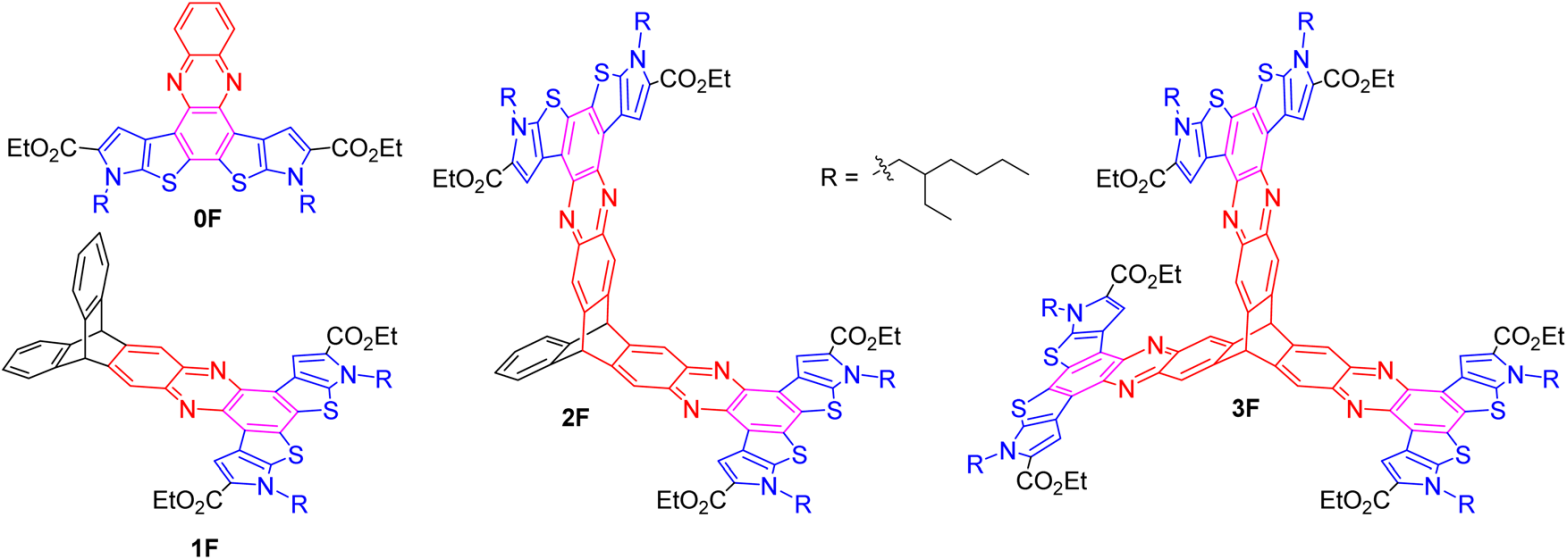
Molecular structures of compounds 0F–3F (R = 2-ethylhexyl). Donor and acceptor regions are indicated in blue and red respectively. The junction aryl ring between the donor and acceptor components is indicated in magenta and is designated as Ar^out^ in the discussion.

Here we present a homologous series of all-ring-fused ICT chromophores 1F–3F which feature triptycene cores and π-extended, T-shaped fins. The non-iptycene 0F has also been synthesised for comparison (Chart 1). This systematic family of molecules further informs how LUMO-homoconjugation can be utilised in multifunctional chromophore design, and also provide a powerful platform to finally explain why localising the LUMO over the core of the 3D scaffold can have such large positive effects on so many photophysical parameters in the first place.

In these new materials, the influence of homoconjugation is not identified in the low energy ICT band of the UV/vis spectra as may be expected from previous studies. Instead, it presents itself as an amplification of the molar absorbance of a progression of bands in the middle of the spectrum. These mid-spectral features of 1F–3F display characteristics of exciton formation in a fashion which is similar in many respects to that of a classic *J*-aggregate. In fact, the properties of 1F–3F agree very well with those of the *J*-aggregate-like model proposed by Pochas *et al.*^28^ in a study which evaluated the properties of branched molecules featuring multiple pendant perylene diimide chromophores initially synthesised by Langhals *et al.*^29–31^

Supramolecular polymers have demonstrated hybrid behaviour between Frenkel exciton and intermolecular charge transfer states, leading to distinctive optical properties^32,33^ and utility in photocatalysis.^34^ Here, 1F–3F further provide valuable new additions to the family of single-molecule materials, most prevalently squaraine derivatives,^35–37^ which show interaction between intramolecular charge transfer and exciton states, and also complement non-ICT chromophores with 3D structures^7,38^ including those which have shown promise towards singlet fission.^39–44^

The key finding here from a chromophore design standpoint is that a homoconjugated framework may be employed to separate and control distinct excitonic and ICT regimes in the UV/vis spectrum of the molecule. This provides an empowered approach to tuning the overall absorbance profile. The ability to control mid-spectral features in particular complements the absorbance-edge tuning strategies which are much more well-established.^45–47^ Our new molecules also display very high fluorescence quantum yields in spite of significant SOC.

## Results and discussion

### Molecular design and synthesis

The molecular design was inspired by archetypical non-fullerene acceptors (NFAs) such as Y6 ^48^ and related quinoxaline-based structures^49,50^ which have intensely absorbing ICT bands at long wavelengths but comparatively weak absorbance in the mid-range. The ladder-type structure employed here utilises cross-conjugated thieno[2,3-*b*]pyrolle heterocycles and results in the outermost triply-fused aryl ring of each fin of the triptycene (Ar^out^ which is coloured magenta in Chart 1) acting as a shared junction between the electron-rich ladder regions and the electron-poor N-heterocyclic core. Ar^out^ therefore has a major contributing role in both the highest occupied molecular orbital (HOMO) and the LUMO.

**Scheme 1:**
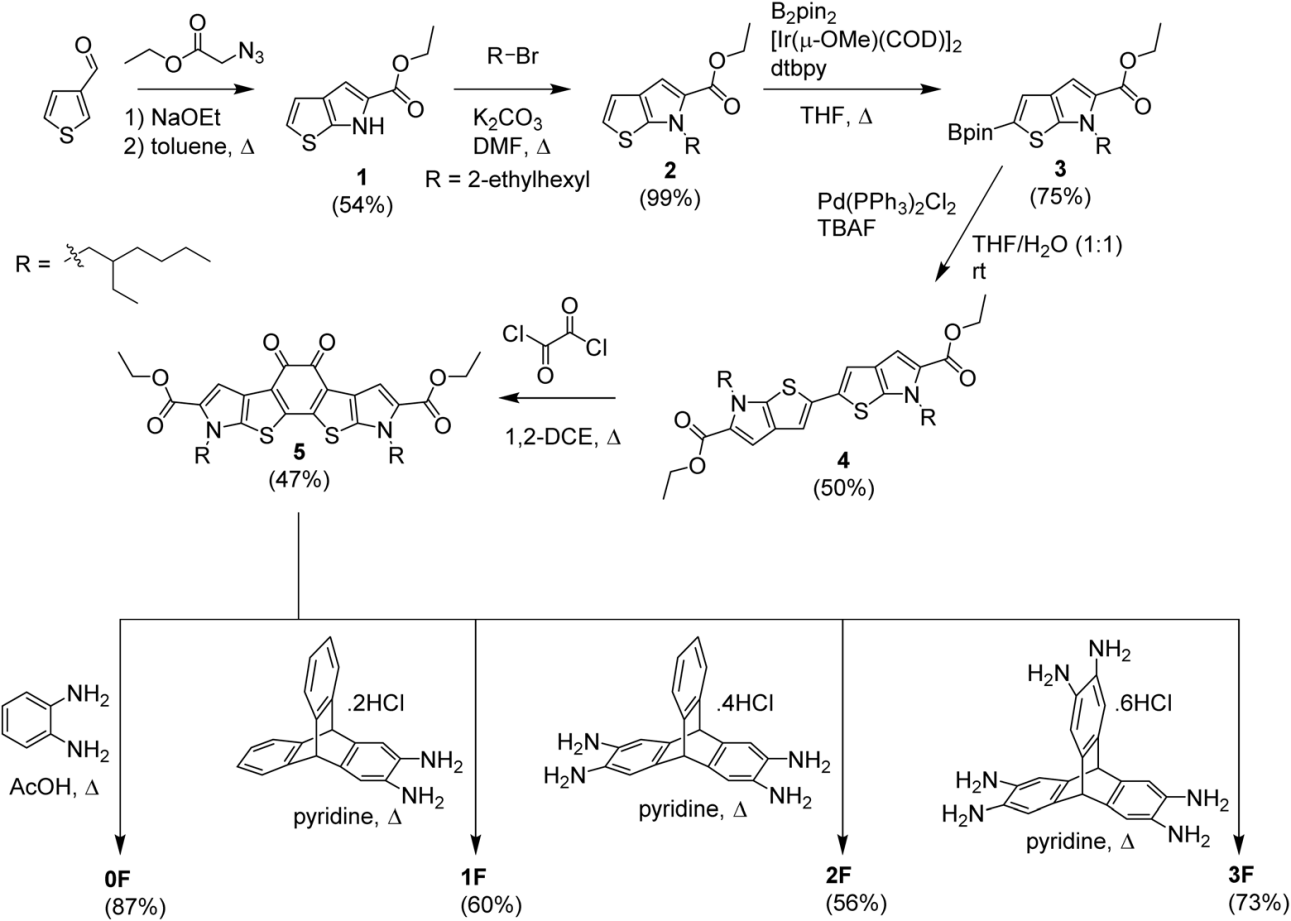
Synthesis of molecules 0F–3F.

The synthesis of 0F–3F is shown in Scheme 1. Experimental procedures are in the SI. Thieno[2,3-*b*]pyrolle-5-ethylcarboxylate 1 was synthesised from ethyl azidoacetate and thiophene-3-carbaldehyde in the presence of NaOEt,^51^ followed by alkylation with 2-ethylhexylbromide in the presence of K_2_CO_3_ to form 2. An iridium catalysed CH-activated cross-coupling^52^ with B_2_pin_2_ generated boronate 3 which was then homocoupled using Pd(PPh_3_)_2_Cl_2_ in the presence of fluoride^53^ to form 4. Finally, direct acylation using oxalyl chloride produced the desired all ring-fused *o*-quinone 5 in modest yield. Condensation reactions to produce phenazines were then completed with 1,2-diaminobenzene and the hydrochloride salts of 2,3-diamino-,^54^ 2,3,5,6-tetraamino-^55^ and 2,3,5,6,11,12-hexaaminotriptycene^54^ to produce 0F, 1F, 2F and 3F respectively.

### X-ray crystallography

Single crystals suitable for structural determination using X-ray crystallography were obtained for 0F and 1F (crystal and experimental details can be found in Table S1). Despite repeated attempts, single crystals of 2F and 3F have not yet been obtained.

**Fig. 1:**
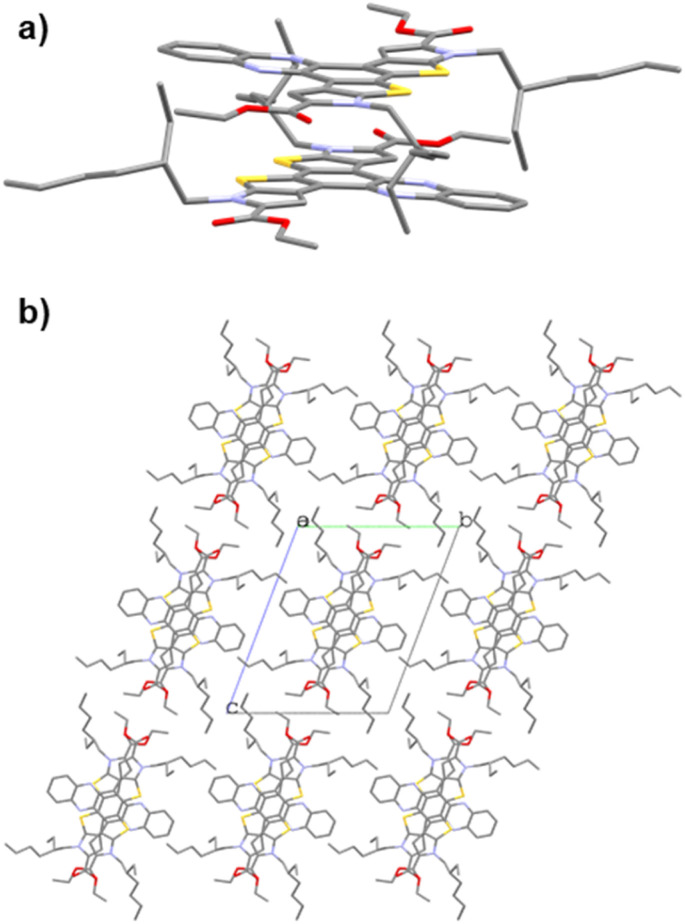
X-ray crystallography determined molecular structure for 0F showing (a) a π-stacked dimer and (b) the view down the *a*-axis. Disorder in the alkyl chains has been omitted for clarity.

In the crystal structure of 0F (Fig. 1) the molecules form columns in the *a*-axis direction. The columns are supported by fairly tight π-stacking interactions between the planar cores of the molecules (interplane distance 3.386 Å) resulting in a head-to-tail alignment between adjacent molecules. The columns are then arranged with interdigitation of the aliphatic groups. This interdigitation is the cause of some disorder in the chains which is not shown in Fig. 1.

**Fig. 2:**
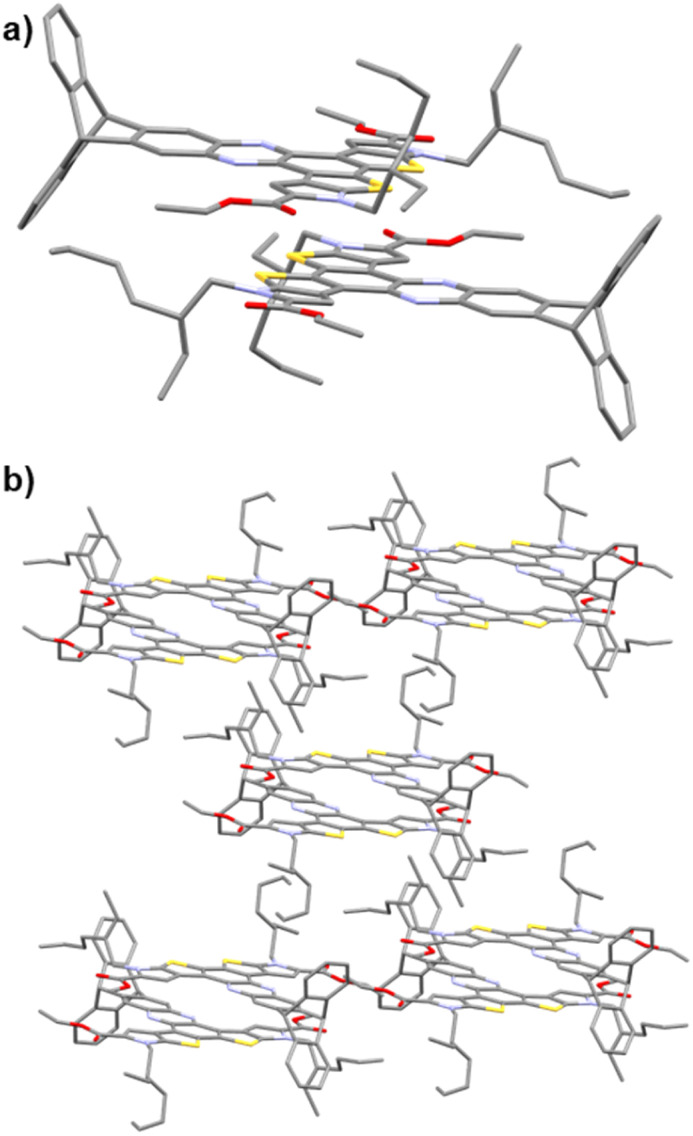
X-ray crystallography determined molecular structures for 1F showing (a) a π-stacked dimer and (b) the packing arrangement of the dimers.

The packing of 1F (Fig. 2) has the triptycene moieties oriented towards the alkyl chain-filled regions. This results in molecules crystallising as discrete head-to-tail π-dimers. To accommodate the bulk of the triptycene moiety, the molecules in each dimer stack less directly on top of each other than in 0F. The π-stacking distance between the 1F molecules remains comparable to that of 0F with an interplane distance of 3.410 Å.

### Steady-state calculations

Density functional theory (DFT) optimised ground state geometries were calculated for 0F–3F using ORCA v5 ^56–58^ with the B3LYP functional, def2-TZVP basis set and def2/J auxiliary basis set.^59,60^ Methyl groups were employed as substitutes for the 2-ethylhexyl chains and all geometries were true minima based on no imaginary frequencies being observed. Additional energy level values and diagrams of relevance to the following discussion can be found in the SI.

**Fig. 3:**
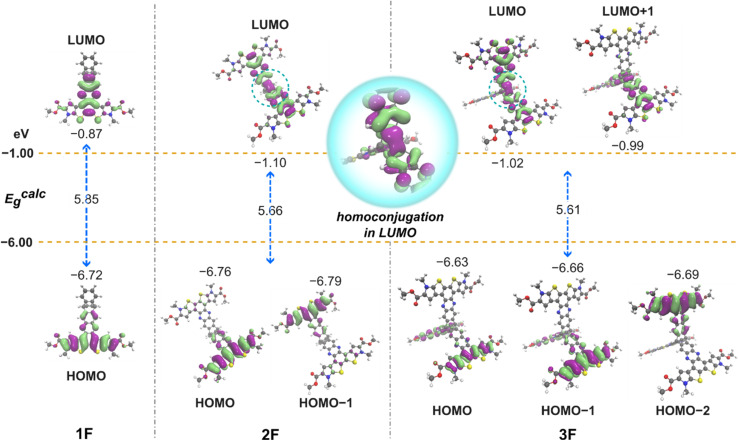
Calculated (B3LYP/def2-TZVP) frontier molecular orbital distributions and energies in eV for 1F–3F.

As anticipated, both the HOMO and LUMO have some density over the Ar^out^ ring for all four molecules. 0F and 1F (1F shown in Fig. 3, for 0F see Fig. S1) have similar profiles with the HOMO localised over the cross-conjugated ladder system and the LUMO over the phenazine ring system. The slightly higher calculated HOMO and LUMO energies, and wider HOMO–LUMO gap (*E*^calc^_g_) for 1F is consistent with observations made of diazaiptycenes in contrast with planar diazaacene congeners.^61^

The influence of increased dimensionality on the frontier orbital energy landscape becomes apparent in 2F where the HOMO and HOMO−1 are now near-degenerate. The HOMO of 2F is positioned over one functionalised fin and the HOMO−1 over the other. In contrast with 0F and 1F the LUMO in 2F is spread across the entire long-axis of the molecule, straddling the homoconjugated core and leading to an orbital distribution which is reminiscent of fully conjugated benzannulated linear tetraazaacenes, albeit disrupted at the core.^62^ In 3F the presence of a third chromophore fin and the influence of the rigid, highly symmetric structure now results in degeneracy of the LUMO and LUMO+1 with each orbital covering two of the three fins. Ushiroguchi *et al.* prepared a triptycene which might be described as a tris(phenazine) fused barrelene and is therefore a good model of the core acceptor framework of 3F. The LUMO manifold of this molecule (calculated at the B3LYP/6-31G* level) bears appropriate similarities to that of 3F (and also 2F) in terms of both the orbital distributions and energies.^63^ Such a distribution of the LUMO suggests that constructive interplay between the fins in 2F and 3F might be expected. Conversely the optoelectronic properties of 0F and 1F should be somewhat similar to each other.

### Cyclic voltammetry

Cyclic voltammetry of 0F–3F (Fig. S3 and Table S3 with additional discussion in SI) correlated well with the DFT where all of the molecules have similar first oxidation and reduction potentials. The influence of the multiple ring systems is apparent in the reductions of 1F–3F where the reduction scan is seen to consist of one reversible wave in 1F then two and three overlapping waves for 2F and 3F respectively.

### Fourier-transform Raman spectroscopy

The 3D distribution of the LUMO over adjacent fins of the triptycene core suggests that we can expect influences on the excited state properties arising from through-space effects. FT-Raman spectroscopy was therefore performed on the solid powder samples of 0F–3F. FT-Raman is a vibrational spectroscopy technique that is very sensitive to molecular structure and conformation, so very small changes in geometry are detectable. Furthermore, because the selection rules of Raman spectroscopy require a change in electron polarizability, it also provides valuable insight into the nature of π-electron distribution in conjugated molecules,^64,65^ making it well suited to understand how the homoconjugated triptycene framework interacts with the rest of the molecule.

**Fig. 4:**
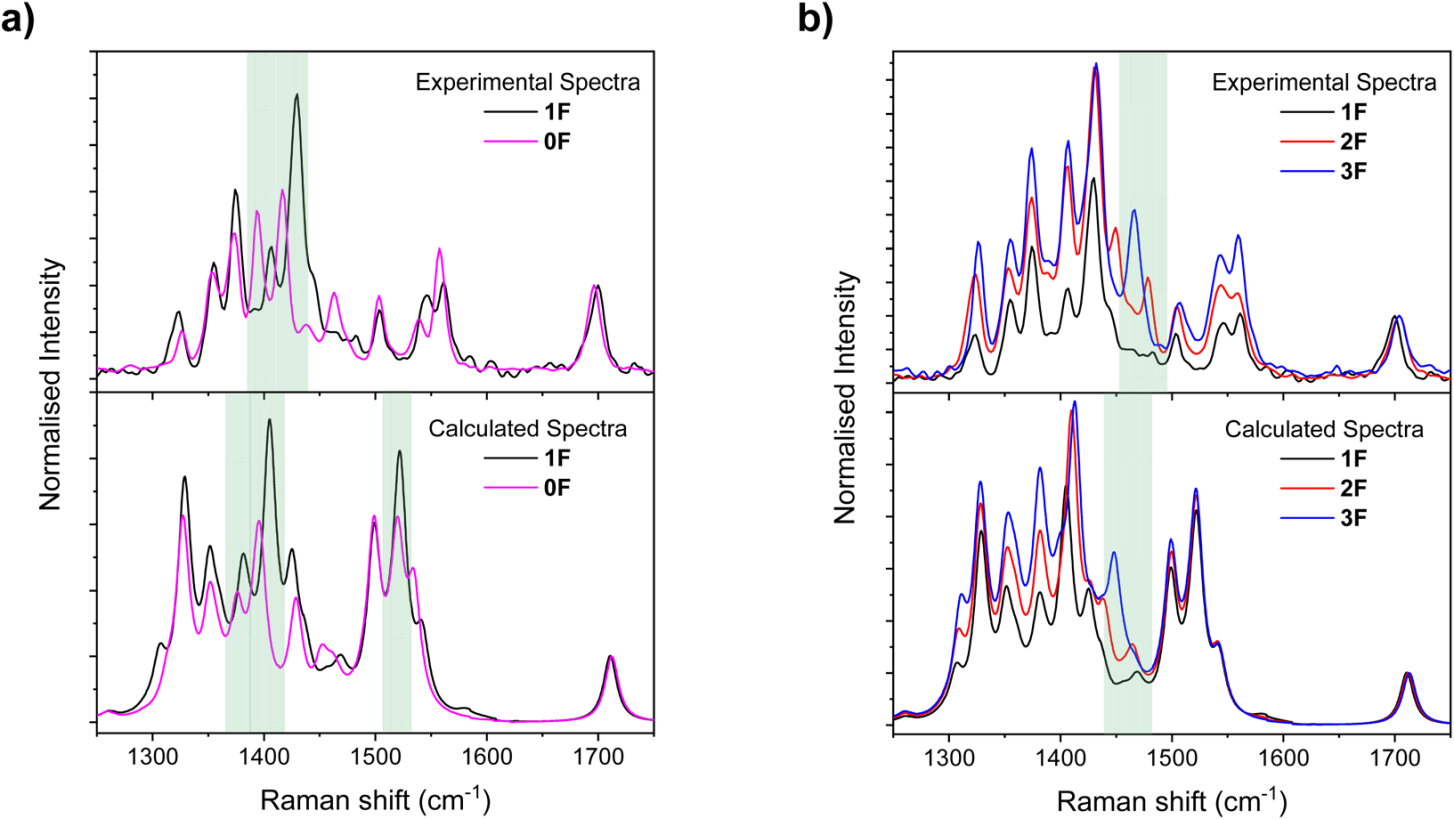
FT-Raman spectra (powder, 1064 nm) and calculated Raman spectra (B3LYP/6-31G(d), frequency scaling factor 0.9613) for: (a) 0F and 1F. The highlighted regions indicate the vibrational modes that have upshifted from 0F to 1F; (b) 1F, 2F, and 3F. The highlighted region indicates the triptycene-localised vibrational mode that progressively downshifts and increases in intensity from 1F to 3F. Data is normalised to the C=O stretching mode at ∼1700 cm^−1^ in both graphs.

Since the Raman spectra of large polyatomic molecules are often complex, we employed computational chemistry to enable normal mode analysis. The calculated Raman spectra (B3LYP/6-31G(d)) are compared to the FT-Raman spectra (absence of resonance conditions) in Fig. 4 and S4. All spectra have been normalised to the C=O stretching mode at ≈1700 cm^−1^, because this bond contributes minimally to the frontier molecular orbitals. For the purposes of the following discussion, the outermost aryl ring of the phenazine system will be designated as Ar^out^ (as described in the introduction) and the internal aryl ring (shared with the triptycene core in 1F–3F) as Ar^in^.

The first interesting observation from the Raman data comes from a comparison between 0F and 1F (Fig. 4(a)). The most intense band in the experimental spectrum for 0F at 1417 cm^−1^ is a C=C stretching mode fully-delocalised over the thienopyrroles and the Ar^out^ ring. The same mode is upshifted in 1F to 1429 cm^−1^. Similarly, another delocalised C=C stretching mode is upshifted from 1393 cm^−1^ in 0F to 1407 cm^−1^ in 1F, and a thienopyrrole-localised C=C mode from 1539 cm^−1^ to 1546 cm^−1^. These upshifts are also reflected in the calculated Raman spectra. The upshifts in the C=C stretching modes indicate that addition of the triptycene unit in 1F has increased the force constant of the C=C bonds in the rest of the molecule. The observation that the fully delocalised modes have a larger upshift than the thienopyrrole-localised C=C mode suggest that it is regions of the phenazine heterocycle that possess the greatest change in bond force constant. Using the calculated bond lengths, a simple HOMA (Harmonic Oscillator Model of Aromaticity) analysis^66,67^ demonstrates a large reduction in the aromaticity of Ar^in^, with the HOMA value decreasing from 0.67 in 0F to 0.52 in 1F (a HOMA value of 1 represents a purely aromatic system, like benzene). This reduction in aromaticity (and subsequent upshifts in the C=C stretching modes) in 1F arises from a subtle distortion of the Ar^in^ ring because of the adjacent triptycene core. The reduced aromaticity may cause less efficient π-conjugation and thus less intramolecular coupling between the triptycene core and the rest of the molecule in 1F.

Next, we assess the changes in the Raman spectra as further fins are added. The comparison of 1F, 2F, and 3F are presented in Fig. 4(b). Most vibrational modes show very little shift through the series. However, there are some very large differences in the experimental Raman spectra between 1445 and 1465 cm^−1^. The weak mode at 1483 cm^−1^ in 1F progressively downshifts to 1479 cm^−1^ then 1466 cm^−1^ for 2F and 3F respectively. Furthermore, the intensity of this mode increases significantly through the series. Importantly, both the increases in intensity and downshifts are replicated in the calculated Raman spectra. This vibrational mode is localised primarily over the triptycene (Fig. S4). The increase in relative intensity of this triptycene mode as the number of fins increases suggests a shift in π-electron density onto the triptycene. Furthermore, the observed downshift is consistent with enhanced π-electron delocalization and a softening of the bond force constants of the triptycene bonds' as the number of fins increases. Due to the lack of through-bond conjugation between fins, this downshift must be entirely facilitated by through-space homoconjugation.

In summary, the Raman data shows that while addition of the triptycene unit disrupts the aromaticity of the connecting quinoxaline unit in 1F, the addition of the second and third fin in 2F and 3F enables homoconjugation-induced coupling between the fins, and a shift in electron density from the peripheral thienopyrrole units to the fused triptycene core.

### Steady-state absorbance and emission

**Fig. 5:**
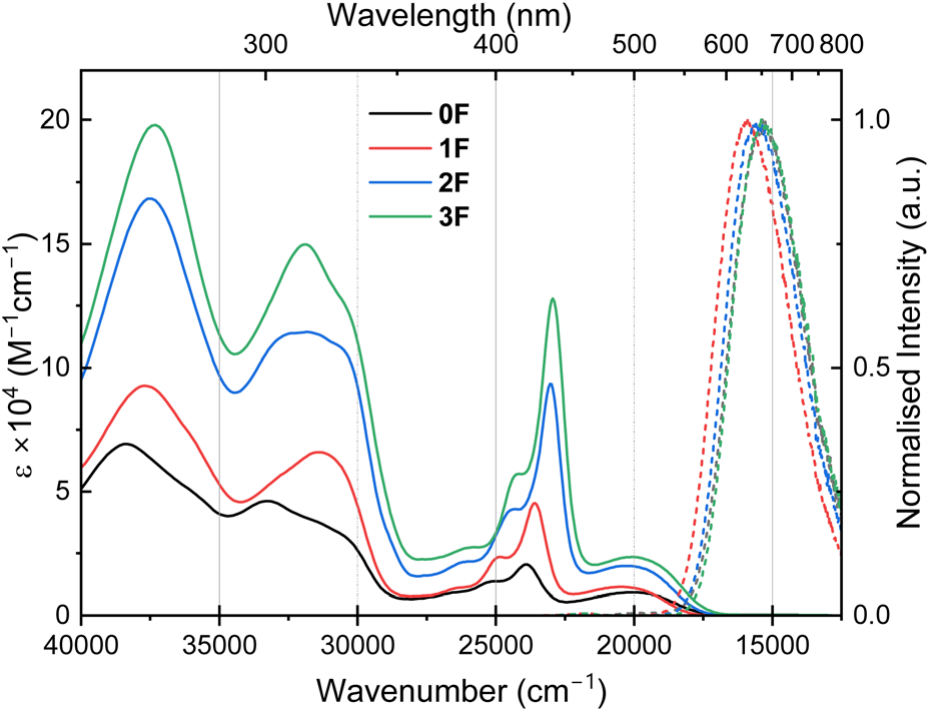
Steady-state UV/vis absorbance (solid lines) and emission (dashed lines, analyte molarity = 1 µM, *λ*_exc_ = 500 nm) for 0F–3F in CH_2_Cl_2_ solution.

The steady state absorbance and emission spectra were first measured in CH_2_Cl_2_ (Fig. 5 and Table 1). Molar absorbance coefficients were obtained using the gradient of Beer–Lambert plots (Fig. S5–S8). In contrast to the shifting profiles of the absorbance spectra (*vide infra*), the emission spectra are very similar across the series 0F–3F. The fluorescence spectra mirror the lowest energy band of the absorbance with no fine structure. Positive solvatochromism is observed with a red-shifting of the emission frequency (*ν*_em_) and increase in the Stokes shift as the solvent polarity increases (Fig. S9–S12). We can therefore conclude that the S_1_ → S_0_ transition and the longest wavelength absorbance are of ICT character.

**Table 1:** Steady-state absorbance and emission properties of 0F–3F in CH_2_Cl_2_ solution

	*ν*_MID_ (cm^−1^) [*ε*_MID_ × 10^3^ M^−1^ cm^−1^]			*ν*_ICT_ (cm^−1^) [*ε*_ICT_ × 10^3^ M^−1^ cm^−1^]	*ν*_em_ a (cm^−1^)	Stokes shift (cm^−1^) [eV]	*E* ^opt^_g_ b (eV)
	*ν*_MID1_	*ν*_MID2_	*ν*_MID3_				
0F	23 923 [20.5]	25 062 [13.8]	26 315 [9.3]	20 049 [9.3]	15 369	4680 [0.58]	2.18
1F	23 584 [45.2]	24 846 [23.5]	26 134 [11.3]	20 470 [11.5]	15 978	4492 [0.57]	2.23
2F	23 041 [93.5]	24 330 [42.9]	25 965 [21.6]	20 283 [19.8]	15.640	4643 [0.58]	2.18
3F	22 935 [127.9]	24 154 [57.1]	25 610 [27.4]	20 060 [23.5]	15 384	4676 [0.58]	2.15

a Analyte molarity = 1 µM, (*λ*_exc_ = 500 nm).

b Optical HOMO–LUMO gap calculated from the onset of absorbance according to: *E*^opt^_g_ = 1240.68 × (*ν*_onset_ × 10^−7^).

The limited change in the Stokes shifts of 0F–3F in any given solvent indicates that the extent of any structural rearrangement of the system in response to the new electronic configuration is similar. This is despite the large difference in effective volume of each molecule and can be rationalised by considering the overall rigidity of the chromophore structure. The influence of the internal molecular free-volume (IMFV) of the triptycene^68,69^ in precluding access of solvent molecules to the interior of the structure provides additional explanation in this respect.

Changes in the profile of the absorbance spectra of 0F–3F are much more compelling. The spectra are composed of three distinct regions, these are intense high energy π → π* transitions above 27 500 cm^−1^ followed by a systematic series of peaks (*ν*_MID_) in the middle of the spectrum with a longest wavelength absorbance (*ν*_MID1_) in the range of 23 000–24 000 cm^−1^. Finally, there is a broad ICT band (*ν*_ICT_) in the range of 20 000–20 500 cm^−1^. We note that 0F has both the shortest *ν*_mid_ and the longest *ν*_ICT_, indicative of slightly stronger dipolar character leading to an optical HOMO–LUMO gap (*E*^opt^_g_) which is comparable to that of 2F.

First, we compare 0F and 1F. The only structural difference between 0F and 1F is the presence of the homoconjugated dibenzobarrelene moiety fused onto the end of 1F. It would be reasonable to expect the spectra of these molecules to be extremely similar. Indeed the *ν*_ICT_ and *ν*_MID_ of 1F are only slightly blueshifted and redshifted respectively and *ε*_ICT_ in 1F is also comparable to that of 0F. However, a stark difference is observed in *ε*_MID_ which more than doubles in intensity from 20.5 × 10^3^ M^−1^ cm^−1^ (0F) to 45.2 × 10^3^ M^−1^ cm^−1^ (1F).

Upon comparing between the triptycene-based compounds 1F–3F more systematic variations in the peak positions and intensities can be discerned. *ν*_MID1_ redshifts by 543 cm^−1^ moving from 1F to 2F but then only 106 cm^−1^ from 2F to 3F. In contrast *ν*_ICT_ redshifts in a step-like fashion by approximately 200 cm^−1^ upon addition of each subsequent fin. Examining the peak intensities, *ε*_MID_ more than doubles moving from 1F (45.2 × 10^3^ M^−1^ cm^−1^) to 2F (93.5 × 10^3^ M^−1^ cm^−1^) followed by a smaller but still large, increase of 34.4 M^−1^ cm^−1^ in 3F (127.9 × 10^3^ M^−1^ cm^−1^). The intensity of the higher energy π → π* transitions also increase in a similar manner to the *ν*_MID_ bands.

To better understand the transitions involved at *ν*_MID_ and *ν*_ICT_ calculations were performed using DFT and linear response time-dependent DFT (LR-TDDFT) within the approximation of the optimally tuned LC-BLYP exchange and correlation functional. A def2-TZVP basis set was used throughout and the solvent assumed to be toluene.

**Fig. 6:**
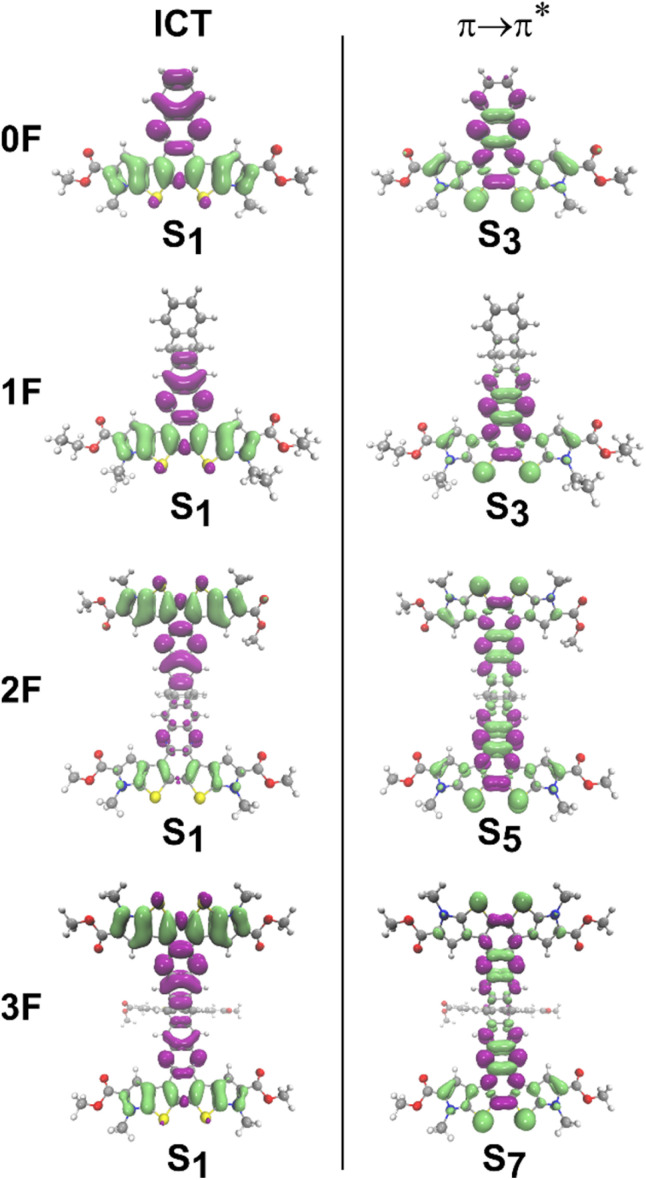
TDDFT calculated difference plots for the S_0_ → S_1_ transitions associated with *ν*_ICT_ and the transitions to higher singlet states associated with *ν*_MID_ (colours: green = electron, magenta = hole).

Difference plots (Fig. 6) confirmed that *ν*_ICT_ pertains to the singlet S_1_ → S_0_ transition from the donor ladder-region(s) into the acceptor phenazine(s). In 2F and 3F there is some MO density spread over two of the fins which suggests the possibility of multiple fins being excited directly into the ICT state simultaneously is unlikely. The calculations confirm that *ν*_MID_ is a π → π* transition occurring to progressively higher (but isoenergetic) singlet excited states as the number of functional fins increases. This is exemplified by S_3_ for both 0F and 1F, S_5_ for 2F and S_7_ for 3F. Large clusters of low energy triplet states are also observed, with the number of low-lying triplet states increasing with the number of fins on the triptycene (Fig. S2 and Table S2).

### Understanding homoconjugation-induced enhancements in optical properties of triptycenes

The overall change observed across the *ν*_MID_ progression of peaks is a stepwise but non-linear increase in *ε* upon addition of subsequent fins. This is similar to those observed for dye aggregates as described by the exciton model pioneered by Kasha.^70^ The electronic structure of an aggregate is dictated by the relative positions of its composite molecules. The model has been extended successfully to several multi-chromophore systems and even conjugated polymers.^2^ In its earliest iterations, it was used to explain the spectroscopic properties of homoconjugated molecules diphenylmethane and triphenylmethane and also much more rigid V-shaped molecules such as Tröger's base.^70^ In the context of triptycenes, exciton coupling has previously been invoked to explain aspects of the optical properties of *E*/*Z* photoisomerising azobenzene substituted triptycenes.^71^

The two classes of aggregate considered most frequently are intermolecular *J*- and *H*-aggregates between entirely (or largely) planar molecules such as perylene diimides (PDIs)^8,72–74^ and merocyanines.^75^ Upon examination of initial experimental studies by Langhals and co-workers on star-like multichromophore systems,^29–31,76^ Pochas *et al.* established that a symmetrical trimeric complex of PDIs held in a trefoil configuration has its own distinct exciton properties classed as neither a *J*- nor *H*-aggregate.^28^ This trefoil structure has a topology similar to that of triptycene so provides an excellent basis by which to understand the variations observed in the *ν*_MID_ portion of the UV/vis spectra between 1F–3F.

The exciton model posits that the transition dipole moment (*M*) in Debye (D) of the aggregate should scale according to square root of the number of monomers (*N*). Normally in the study of aggregate dyes, the long wavelength edge of the absorbance spectra can conveniently be used to extract *M*^Abs^. However, for the molecules presented here, the low energy features of the UV/vis are entirely ICT in nature and any exciton-induced enhancements are being observed in the middle of the spectrum.

**Fig. 7:**
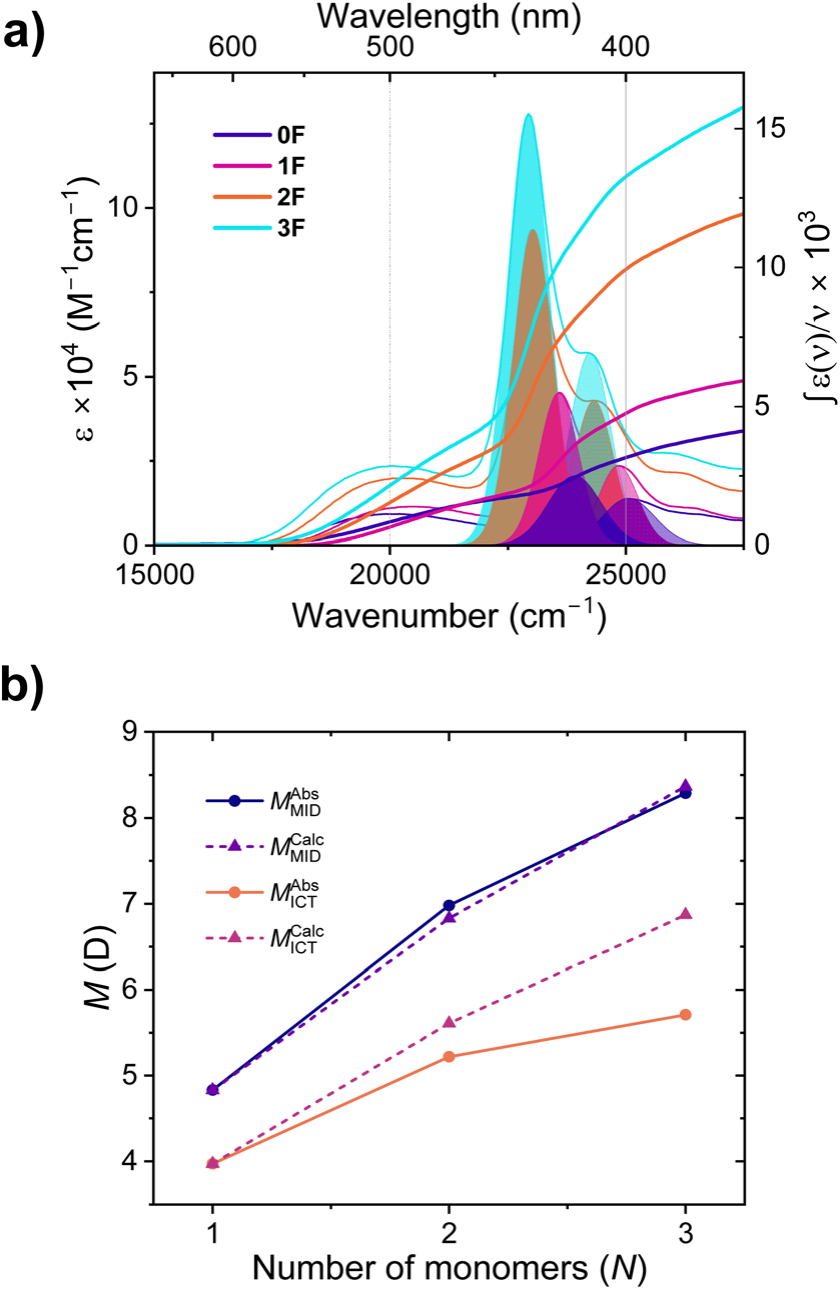
(a) UV/vis absorbance spectrum (left-hand axis) of 0F–3F in CH_2_Cl_2_ solution overlaid with the integral of *ε*/*ν* (right hand axis) which was then used to calculate the experimental transition dipole moments *M*^Abs^_ICT_ and *M*^Abs^_MID_ and also showing the Gaussian peak fittings (shaded peaks) used to calculate *S*_AN_. (b) A close agreement is observed for *ν*_MID_ between the experimentally determined transition dipole moments compared to those predicted from the exciton model.

To overcome this, the entire spectrum was integrated (Fig. 7(a)) and the integrands of both the highest intensity peak of *ν*_MID1_ and the entire ICT band *ν*_ICT_ were used in eqn (1) (where *n* is the refractive index, *n* = 1.424 for CH_2_Cl_2_ (ref. 77) and *ν* is the wavenumber in cm^−1^) to calculate the transition dipole moment for the absorbance bands *ν*_MID1_ and *ν*_ICT_ to obtain *M*^Abs^_MID_ and *M*^Abs^_ICT_ respectively (Table 2).^78^ The lowest energy points on either side of the peaks in question were taken as the limits of integration.1
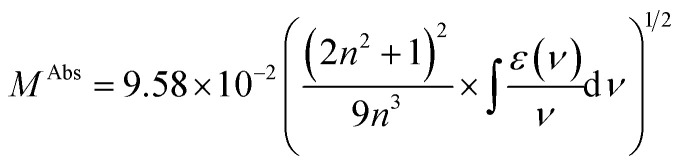


**Table 2:** Experimental transition dipole moments (*M*^Abs^_MID_ and *M*^Abs^_ICT_) for 0F–3F, the *S*_AN_ and *R*_abs_ values for 1F–3F and the calculated transition dipole moments (*M*^Calc^_MID_ and *M*^Calc^_ICT_) for 1F–3F

	*N*	Experimental values				Calculated values	
		*M* ^Abs^_MID_(D)^a^ [*f*_osc_]^b^	*M* ^Abs^_ICT_(D)^a^ [*f*_osc_]^b^	*S*_AN_	*R*_abs_	*M* ^Calc^_MID_(D)^c^	*M* ^Calc^_ICT_(D)^d^
0F	—	3.50 [0.14]	3.93 [0.15]	—	—	—	—
1F	1	4.83 [0.26]	3.97 [0.15]	1.00	1.92	4.83	3.97
2F	2	6.98 [0.53]	5.22 [0.26]	1.05	2.18	6.83	5.61
3F	3	8.29 [0.74]	5.71 [0.31]	0.99	2.23	8.37	6.87

a Calculated according to eqn (1).

b Calculated according to eqn (2).

c Calculated as the product of √*N* (where *N* is the number of monomers) and *M*^Abs^_MID_ of 1F as the monomer. *d* Calculated as the product of √*N* (where *N* is the number of monomers) and *M*^Abs^_ICT_ of 1F as the monomer.

d An updated Table 2 was attached in response to Q4.

Again, we first compare 0F and 1F. Their values of *M*^Abs^_ICT_ are found to be very similar but *M*^Abs^_MID_ are significantly different. That there is a difference at all further demonstrates that the triptycene-framework is not an innocent bystander in the optoelectronic properties of these materials. From this we can draw an initial important conclusion which is that 1F is a more appropriate molecule to consider as the monomer (*N* = 1) for comparison with the dimeric and trimeric “aggregates” 2F (*N* = 2) and 3F (*N* = 3). This observation also suggests that the preparation of 1F-like triptycene-fused molecules will be much more informative than non-triptycene 0F-like homologues as model compounds for any 2F- or 3F-like systems.

The experimental values of *M*^Abs^_MID_ and *M*^Abs^_ICT_ obtained for 1F were then scaled by √*N* to obtain calculated values *M*^Calc^_MID_ and *M*^Calc^_ICT_ for 1F–3F (Fig. 7(b) and Table 3). The experimental and calculated values for *M*_MID_ are in excellent agreement while those for *M*_ICT_ are not. The absence of √*N* scaling for *µ*_CT_ suggests that exciton interactions are only occurring upon population of the higher excited states represented by *ν*_MID_. Trends in the variation of *M*_ICT_ for 1F–3F arise from the incremental increase in acceptor strength as further pyrazine rings are fused onto the triptycene.

**Table 3:** Photophysical properties of 0F–3F in dilute toluene solution

	*E*_S1_ (eV)	*ϕ*_f_ (%)	*τ*_f_ (ns)	*k*_r_ (10^7^ s^−1^)	*k*_nr_ (10^7^ s^−1^)	*τ*_T_ (µs)	*M* ^em^_ICT_(D)^a^
0F	2.25	66 ± 4	18.0	3.7 ± 0.2	1.9 ± 0.2	740 ± 140	2.74
1F	2.32	75 ± 10	18.0	4.2 ± 0.6	1.4 ± 0.6	880 ± 140	2.78
2F	2.27	68 ± 12	19.7	3.5 ± 0.7	1.6 ± 0.6	570 ± 40	2.63
3F	2.23	86 ± 12	20.6	4.2 ± 0.6	0.7 ± 0.6	790 ± 90	2.94

a Calculated using eqn (3).

Oscillator strengths (*f*_osc_) for the *ν*_MID_ and *ν*_ICT_ transitions were subsequently calculated using eqn (2) and were included in Table 1.^79^

2 *f*_ocs_ = 4.702 × 10^−7^*ν*|*M*^Abs^|^2^

The variation in *f*_osc_ with respect to *N* is also consistent with Pochas' model. The *f*_osc_ is enhanced relative to that of the monomer in each case, but the extent of enhancement decreases as *N* increases. For a true *J*-aggregate the extent of enhancement should increase with *N*. It is also observed that the areas of Gaussian peaks fitted to the *ν*_MID1_ for 2F and 3F scale to a decreasing number of *N* times the monomer area as *N* increases. This is expressed as the ratio *S*_AN_ and is equal to 1.05 and 0.99 for 2F and 3F respectively. The *ν*_MID1_ peak redshifts with increasing *N* and the ratio of *f*_osc_ of the *ν*_MID1_ and *ν*_MID2_ bands (*R*_abs_) increases with *N*.

Overall, these observations demonstrate generality to the exciton model proposed for a trefoil system, which agrees very well with the *ν*_MID_ region for 1F–3F. Any deviations from this model are minor and due to structural differences imposed by the triptycene core when compared to the PDI model systems. These include rigid 120° angles between the planes of the chromophores, and the fact that any twisting between the chromophores is no longer possible. This finally provides some explanation for super-summative enhancements which have previously been observed in triptycene based materials, and reveals a new approach to tuning the mid-spectral profile of functional chromophores.

### Time-resolved photophysics

Finally, we turn our attention to understanding the excited state properties through time-resolved methods. Due to the low boiling point of CH_2_Cl_2_, and requirement to exclude oxygen *via* freeze–pump–thaw methods, toluene was adopted as an appropriate solvent from which to obtain the time-resolved measurements.

The fluorescence photoluminescence quantum yield (*Φ*_f_), fluorescence lifetime (*τ*_fl_), radiative decay rates (*k*_r_) and non-radiative decay rates (*k*_nr_) are shown in Table 3. *Φ*_f_ was high for all of the compounds, most notably for the largest molecule 3F with a value of 86 ± 12% which is comparable with some of the most strongly emitting superradiant *J*-aggregates.^80–83^ The relative differences with 1F and 2F indicate that the core of the high-symmetry structure 3F is well-shielded from the external solution thereby circumventing intermolecular loss pathways. The planar structure of 0F permits more direct comparison with other dithienophenazines. In general, chromophores based around this ring-system display low *Φ*_f_ (typically 0.5–10%)^84–86^ in solution with a handful of examples having *Φ*_f_ >10% (ref. 87 and 88) so 0F is notably luminescent in any case.

To complement the analysis of the UV/vis spectra (*vide supra*) transition dipole moments of the ICT emission band (*M*^em^_ICT_) were calculated using *k*_r_ and the emission wavenumber *ν*_em_ according to eqn (3) (ref. 89) (note that this equation requires use of the Planck constant in *h* = 6.626 × 10^−27^ erg s and upon solving for *M* a conversion factor of 10^18^ must be applied to convert from statC.cm to Debye, for toluene *n* = 1.497 (ref. 76)). This gave similar results across the series and had values consistent with ICT emission which again supports the idea of a common radiative deactivation pathway for 0F–3F.3
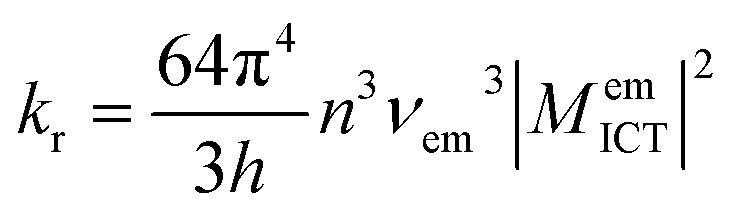


The singlet lifetimes of 0F and 1F are identical but *τ*_fl_ then gets incrementally longer moving to 2F and 3F. This may seem surprising as the radiative lifetime of the aggregate should decrease with increasing *N*.^2,80,81,90^ We rationalise this by considering that, as the radiative step is occurring through the ICT state S_1_, increasingly fast internal conversion may well be occurring from the *ν*_MID_ exciton manifold into S_1_ but the overall rate of emission is then effectively throttled by the *k*_r_ of the ICT state. The identical lifetimes of 0F and 1F support this hypothesis. The extracted *k*_r_ values are therefore also rather similar across the series 0F–3F in the range of 3–5 × 10^7^ s^−1^. The *k*_nr_ values are quite similar for 0F–2F but that of 3F appears to be meaningfully slower. Since high *k*_nr_ is detrimental to OPV performance,^91,92^ this suggests that further development of this design concept may lead to promising NFA materials.

Finally, transient absorbance spectroscopy (TAS) was employed to identify triplet formation in 0F–3F with a view to explaining the dense manifolds of low-lying triplet states and significant spin–orbit coupling (SOC) induced intersystem crossing (ISC) which have previously been observed in ICT iptycenes.

**Fig. 8:**
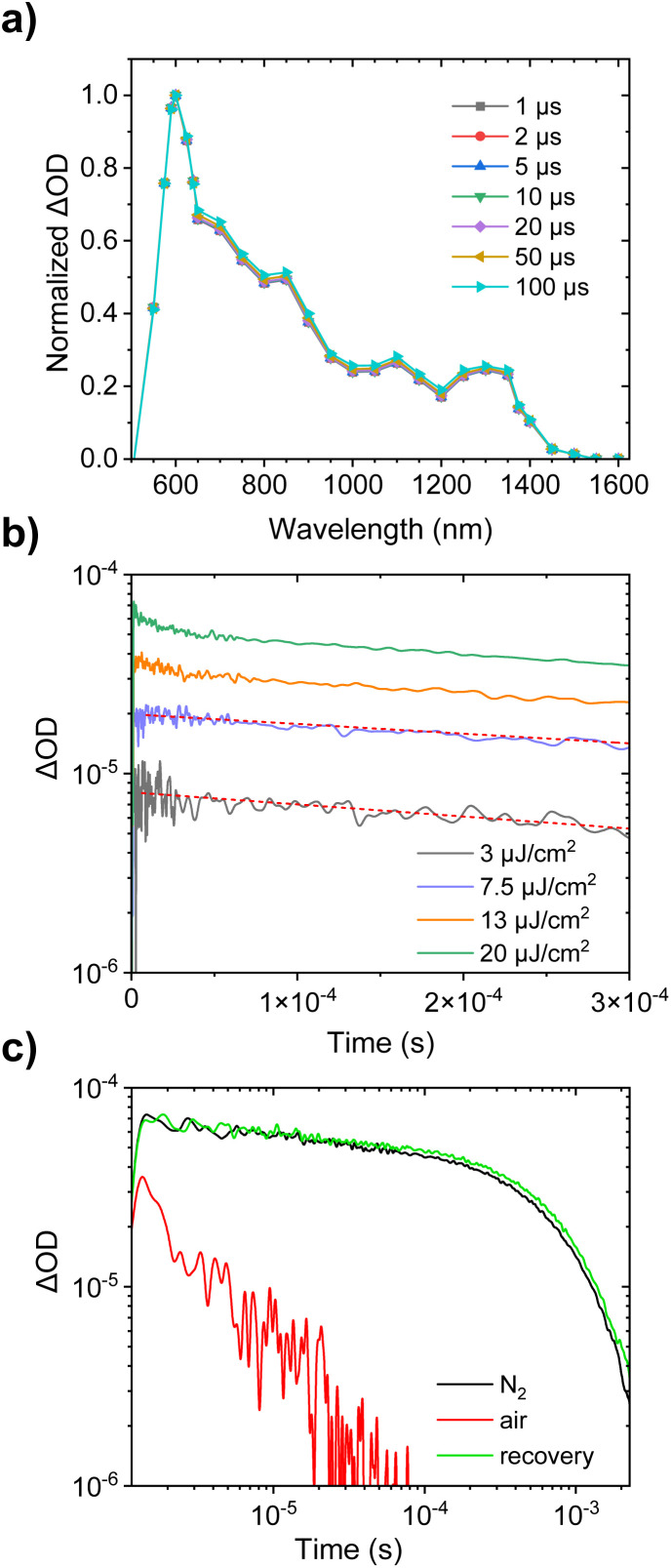
(a) Normalised TA spectra of 3F (toluene) solution, excited at 415 nm, 20 µJ cm^−2^, (b) energy dependence of 3F (toluene) solution, excited at 415 nm, probed at 700 nm (c) Reversible oxygen quenching of the long-lived decay component of 3F.

Unfortunately, due to the intense emission of the molecules which persist for longer than the interval between TA pump pulses, potentially crucial insight into the triplet formation could not be obtained from ps-TAS measurements. However, as the *τ*_fl_ of the materials are on the order of tens of ns, such intense emission did not present an issue in obtaining µs-TAS. All four molecules show similar µs-TA spectra: a strong band at 600 nm with vibronic structure and a weaker band at 1300 nm, as exemplified for 3F in Fig. 8 (the relevant plots for 0F–2F can be found in Fig. S13–S15). The 600 nm band is very close to the negative ground state bleach (GSB) region which may obscure its true position and intensity. Note that the µs-TA spectra excited at 415 nm (*ν*_MID_) and 500 nm (*ν*_ICT_) exhibit similar shapes and intensities. There is no significant spectral evolution in any of the samples, which strongly suggests that both 600 nm and 1300 nm bands can be attributed to only one type of transient species. For all four molecules, the kinetics at low fluences (3–7 µJ cm^−2^) are monoexponential, which is characteristic of triplet excitons on these timescales. Second-order processes (such as exciton–exciton annihilation) at early times are apparent at higher fluences. Oxygen-dependent measurements revealed strong but reversible oxygen quenching of the TA signal, both in terms of intensity and lifetime. This confirms the presence of triplets in all of the solutions.

The 7.5 µJ cm^−2^ data was used to compare the decay kinetics between the different samples, as it exhibits the best signal-to-noise ratio without showing any second order effects. The triplet lifetimes were estimated (Table 3) and are quite similar across the series (Fig. S16). This suggests that similar triplet decay pathways are present in each molecule, consistent with their similar energetics and similar fluorescence properties. While the TAS suggests some triplet populations forming under excitation at 415 nm as supported by the TDDFT, the rapid *k*_r_ and high *Φ*_f_ of the molecules suggests that fast internal conversion to S_1_ followed by fluorescence is the predominant relaxation pathway. The ISC occurring into triplet states, especially from the higher singlet states, will be a minor pathway.

Concerning the origin of the SOC enhancements which are facilitating the ISC observed in ICT-triptycenes, the spin–orbit, charge-transfer intersystem crossing (SOCT-ISC)^77,93–95^ mechanism has previously been invoked to explain similar observations in related V-shaped molecules,^96^ and slip-stacked dimers of PDIs which also demonstrate excitonic effects.^97^ Also, the fin structure is built around phenazine which itself is an effective triplet sensitiser^98^ arising from mixing of the S_1_ n → π* and T_1_ π → π* states in accordance with El-Sayed's rule.^99,100^ It is likely that a combination of both factors are at play in these molecules.

## Conclusions

To conclude, we have presented a systematic series of triptycene-based homoconjugated chromophores 0F–3F which show an interplay between a low energy ICT transition and a sequence of π → π* transitions which are located in the middle of the absorption spectrum. The variations in intensity and position of the mid-spectrum are described well as an intramolecular exciton state. This has finally provided an explanation for the enhancements in various photophysical properties observed in LUMO-homoconjugated chromophores. It seems likely that this is a general phenomenon for this class of material.

These new molecules are also highly fluorescent with 3F having a near-unity photoluminescence quantum yield within experimental error. The results obtained suggest that intramolecular loss mechanisms through the triplet manifold in 3F are circumvented by very rapid internal conversion from higher singlet states to S_1_ prior to radiative decay through the ICT channel. Collisional deactivation is also decreased by its bulky, well-shielded structure leading to very efficient luminescence overall. This provides an important contrast with existing studies concerning the modulation of ICT between distinct donor and acceptor fins.^17,101–105^

The key findings of separation between the excitonic and ICT regimes reveals new structure-property relationships to be explored. These features should be independently tunable so the ICT band can be shifted or rendered more intense using the typical band-edge tuning strategies, while the exciton progression can be similarly tuned through modifications at the triptycene core. Benefits that might be realised by harnessing the distinct photophysical behaviour of exciton and ICT states simultaneously in a single molecule include circumventing non-radiative decay efficiency losses in low energy gap emitters for OLEDs and bioimaging,^106–108^ the ability to maximise the efficiency of broadband absorbance while avoiding triplet-mediated loss mechanisms in organic solar cell materials,^109^ and straightforward colour tuning and dissymmetry factor enhancement in chiroptical materials,^32^ rendering this an empowered approach to new functional chromophore design.

## Author contributions

I. A. W. conceptualised the research. S. W. synthesised the molecules and completed the electrochemistry and steady-state absorbance spectroscopy. H. I. G. and T. M. C. performed the Raman and time-resolved spectroscopy. G. S. N., E. M. D. & S. J. C. completed the X-ray crystallography. T. J. P. conducted the computational analysis. M. K. E. completed photoluminescence and additional steady-state absorbance spectroscopy. I. A. W. and T. M. C. wrote the manuscript. All authors contributed to discussing the results obtained and commenting on the manuscript.

## Conflicts of interest

There are no conflicts to declare.

## Supplementary Material

SC-OLF-D6SC01073C-s001

SC-OLF-D6SC01073C-s002

## Data Availability

CCDC 2495341 (0F) and 2502203 (1F) contain the supplementary crystallographic data for this paper.^111*a*,*b*^ The data required to form the conclusions of this study are present in the paper itself, or are included in the supplementary information (SI). Additional data can be provided by the corresponding author(s) upon request. Supplementary information is available. See DOI: https://doi.org/10.1039/d6sc01073c.

## References

[cit1] Kanibolotsky A. L., Perepichka I. F., Skabara P. J. (2010). Chem. Soc. Rev..

[cit2] Hestand N. J., Spano F. C. (2018). Chem. Rev..

[cit3] Parkinson P., Kondratuk D. V., Menelaou C., Gong J. Q., Anderson H. L., Herz L. M. (2014). J. Phys. Chem. Lett..

[cit4] Brixner T., Hildner R., Köhler J., Lambert C., Würthner F. (2017). Adv. Energy Mater..

[cit5] Scholes G. D., Ghiggino K. P. (2002). J. Phys. Chem..

[cit6] Zhang G., Lami V., Rominger F., Vaynzof Y., Mastalerz M. (2016). Angew. Chem., Int. Ed..

[cit7] Lv L., Sun W., Jia Z., Zhang G., Wang F., Tan Z. a., Zhang L. (2020). Mater. Chem. Front..

[cit8] Ramanan C., Kim C. H., Marks T. J., Wasielewski M. R. (2014). J. Phys. Chem. C.

[cit9] Yoo H., Furumaki S., Yang J., Lee J. E., Chung H., Oba T., Kobayashi H., Rybtchinski B., Wilson T. M., Wasielewski M. R., Vacha M., Kim D. (2012). J. Phys. Chem. B.

[cit10] Wu Q., Zhao D., Yang J., Sharapov V., Cai Z., Li L., Zhang N., Neshchadin A., Chen W., Yu L. (2017). Chem. Mater..

[cit11] Menke E. H., Lami V., Vaynzof Y., Mastalerz M. (2016). Chem. Commun..

[cit12] Peurifoy S. R., Castro E., Liu F., Zhu X. Y., Ng F., Jockusch S., Steigerwald M. L., Echegoyen L., Nuckolls C., Sisto T. J. (2018). J. Am. Chem. Soc..

[cit13] Zhang J., Li Y., Huang J., Hu H., Zhang G., Ma T., Chow P. C. Y., Ade H., Pan D., Yan H. (2017). J. Am. Chem. Soc..

[cit14] Meng D., Fu H., Xiao C., Meng X., Winands T., Ma W., Wei W., Fan B., Huo L., Doltsinis N. L., Li Y., Sun Y., Wang Z. (2016). J. Am. Chem. Soc..

[cit15] Lee J. W., Park J. S., Jeon H., Lee S., Jeong D., Lee C., Kim Y. H., Kim B. J. (2024). Chem. Soc. Rev..

[cit16] Yu H., Arunagiri L., Zhang L., Huang J., Ma W., Zhang J., Yan H. (2020). J. Mater. Chem. A.

[cit17] Kawasumi K., Wu T., Zhu T., Chae H. S., Van Voorhis T., Baldo M. A., Swager T. M. (2015). J. Am. Chem. Soc..

[cit18] Kumar S., Franca L. G., Stavrou K., Crovini E., Cordes D. B., Slawin A. M. Z., Monkman A. P., Zysman-Colman E. (2021). J. Phys. Chem. Lett..

[cit19] Madhusudana Rao K., Ramaraghavulu R., Kolli D., Babu S. H., Vattikuti S. V. P. (2025). J. Mater. Chem. C.

[cit20] Montanaro S., Pander P., Mistry J.-R., Elsegood M. R. J., Teat S. J., Bond A. D., Wright I. A., Congrave D. G., Etherington M. K. (2022). J. Mater. Chem. C.

[cit21] Tang X., Cui L. S., Li H. C., Gillett A. J., Auras F., Qu Y. K., Zhong C., Jones S. T. E., Jiang Z. Q., Friend R. H., Liao L.-S. (2020). Nat. Mater..

[cit22] Spuling E., Sharma N., Samuel I. D. W., Zysman-Colman E., Bräse S. (2018). Chem. Commun..

[cit23] Yu M., Zhu X., Zeng J., Liu H., Huang R., Zhuang Z., Shen P., Zhao Z., Tang B. Z. (2021). J. Mater. Chem. C.

[cit24] Mistry J.-R., Montanaro S., Wright I. A. (2023). Mater. Adv..

[cit25] See: https://goldbook.iupac.org/terms/view/H02842

[cit26] Muller P. (1994). Pure Appl. Chem..

[cit27] Montanaro S., Congrave D. G., Etherington M. K., Wright I. A. (2019). J. Mater. Chem. C.

[cit28] Pochas C. M., Kistler K. A., Yamagata H., Matsika S., Spano F. C. (2013). J. Am. Chem. Soc..

[cit29] Langhals H., Gold J. (1996). J. Prakt. Chem./Chem.- Ztg..

[cit30] Langhals H., Wagner C., Ismael R. (2001). New J. Chem..

[cit31] Langhals H. (2005). Helv. Chim. Acta.

[cit32] Han J., Fujikawa S., Kimizuka N. (2024). Angew. Chem., Int. Ed..

[cit33] Wehner M., Rohr M. I. S., Buhler M., Stepanenko V., Wagner W., Würthner F. (2019). J. Am. Chem. Soc..

[cit34] Hestand N. J., Kazantsev R. V., Weingarten A. S., Palmer L. C., Stupp S. I., Spano F. C. (2016). J. Am. Chem. Soc..

[cit35] Maeda T., Nguyen T. V., Kuwano Y., Chen X., Miyanaga K., Nakazumi H. (2018). J. Phys. Chem. C.

[cit36] Lambert C., Hoche J., Schreck M. H., Holzapfel M., Schmiedel A., Selby J., Turkin A., Mitric R. (2021). J. Phys. Chem. A.

[cit37] Ceymann H., Balkenhohl M., Schmiedel A., Holzapfel M., Lambert C. (2016). Phys. Chem. Chem. Phys..

[cit38] Scholes G. D., Ghiggino K. P., Oliver A. M., Paddon-Row M. N. (2002). J. Am. Chem. Soc..

[cit39] Ahrens L., Wollscheid N., Han J., Kefer O., Rominger F., Roozbeh A., Freudenberg J., Dreuw A., Bunz U. H. F., Buckup T. (2021). J. Phys. Chem. B.

[cit40] Cook J. D., Carey T. J., Arias D. H., Johnson J. C., Damrauer N. H. (2017). J. Phys. Chem. A.

[cit41] Korovina N. V., Das S., Nett Z., Feng X., Joy J., Haiges R., Krylov A. I., Bradforth S. E., Thompson M. E. (2016). J. Am. Chem. Soc..

[cit42] Krishnapriya K. C., Musser A. J., Patil S. (2018). ACS Energy Lett..

[cit43] Kumarasamy E., Sanders S. N., Tayebjee M. J. Y., Asadpoordarvish A., Hele T. J. H., Fuemmeler E. G., Pun A. B., Yablon L. M., Low J. Z., Paley D. W., Dean J. C., Choi B., Scholes G. D., Steigerwald M. L., Ananth N., McCarney D. R., Sfeir M. Y., Campos L. M. (2017). J. Am. Chem. Soc..

[cit44] Rapp M. R., Weiß R., Wollny A. S., Guldi D. M., Bettinger H. F. (2024). Adv. Funct. Mater..

[cit45] Perepichka D. F., Bryce M. R. (2005). Angew. Chem., Int. Ed..

[cit46] Hashemi D., Ma X., Ansari R., Kim J. (2019). Phys. Chem. Chem. Phys..

[cit47] Dou L., Liu Y., Hong Z., Li G., Yang Y. (2015). Chem. Rev..

[cit48] Yuan J., Zhang Y., Zhou L., Zhang G., Yip H.-L., Lau T.-K., Lu X., Zhu C., Peng H., Johnson P. A., Leclerc M., Cao Y., Ulanski J., Li Y., Zou Y. (2019). Joule.

[cit49] Zhou Z., Liu W., Zhou G., Zhang M., Qian D., Zhang J., Chen S., Xu S., Yang C., Gao F., Zhu H., Liu F., Zhu X. (2020). Adv. Mater..

[cit50] Chen H., Liang H., Guo Z., Zhu Y., Zhang Z., Li Z., Cao X., Wang H., Feng W., Zou Y., Meng L., Xu X., Kan B., Li C., Yao Z., Wan X., Ma Z., Chen Y. (2022). Angew. Chem., Int. Ed..

[cit51] Vogt A., Henne F., Wetzel C., Mena-Osteritz E., Bauerle P. (2020). Beilstein J. Org. Chem..

[cit52] Robbins D. W., Hartwig J. F. (2012). Org. Lett..

[cit53] Finn M. G., Punna S., Díaz D. D. (2004). Synlett.

[cit54] Chong J. H., MacLachlan M. J. (2006). Inorg. Chem..

[cit55] White N. G., MacLachlan M. J. (2015). J. Org. Chem..

[cit56] Lehtola S., Steigemann C., Oliveira M. J. T., Marques M. A. L. (2018). SoftwareX.

[cit57] Neese F. (2022). Wiley Interdiscip. Rev.:Comput. Mol. Sci..

[cit58] Valeev E. F. , Libint, 2.8.0 edn, 2022

[cit59] Weigend F. (2006). Phys. Chem. Chem. Phys..

[cit60] Weigend F., Ahlrichs R. (2005). Phys. Chem. Chem. Phys..

[cit61] Biegger P., Stolz S., Intorp S. N., Zhang Y., Engelhart J. U., Rominger F., Hardcastle K. I., Lemmer U., Qian X., Hamburger M. (2015). J. Org. Chem..

[cit62] Muller M., Reiss H., Tverskoy O., Rominger F., Freudenberg J., Bunz U. H. F. (2018). Chem.–Eur. J..

[cit63] Ushiroguchi R., Shuku Y., Suizu R., Awaga K. (2020). Cryst. Growth Des..

[cit64] Pagano K., Kim J. G., Luke J., Tan E., Stewart K., Sazanovich I. V., Karras G., Gonev H. I., Marsh A. V., Kim N. Y., Kwon S., Kim Y. Y., Alonso M. I., Dörling B., Campoy-Quiles M., Parker A. W., Clarke T. M., Kim Y.-H. (2024). Nat. Commun..

[cit65] Wood S., Hollis J. R., Kim J.-S. (2017). J. Phys. D: Appl. Phys..

[cit66] Krygowski T. M. (1993). J. Chem. Inf. Comput. Sci..

[cit67] Marin-Beloqui J. M., Gomez S., Gonev H. I., Comi M., Al-Hashimi M. (2023). Chem. Sci..

[cit68] Hilton C. L., Jamison C. R., Zane H. K., King B. T. (2009). J. Org. Chem..

[cit69] Tsui N. T., Paraskos A. J., Torun L., Swager T. M., Thomas E. L. (2006). Macromolecules.

[cit70] Kasha M., Rawls H. R., Ashraf El-Bayoumi M. (1965). Pure Appl. Chem..

[cit71] Kunz A., Oberhof N., Scherz F., Martins L., Dreuw A., Wegner H. A. (2022). Chem.–Eur. J..

[cit72] Ahrens M. J., Sinks L. E., Rybtchinski B., Liu W., Jones B. A., Giaimo J. M., Gusev A. V., Goshe A. J., Tiede D. M. (2004). J. Am. Chem. Soc..

[cit73] Hecht M., Würthner F. (2021). Acc. Chem. Res..

[cit74] Rybtchinski B., Sinks L. E., Wasielewski M. R. (2004). J. Phys. Chem. A.

[cit75] Würthner F. (2016). Acc. Chem. Res..

[cit76] Langhals H., Jona W. (1998). Angew. Chem., Int. Ed..

[cit77] Physical Constants of Organic Compounds, in CRC Handbook of Chemistry and Physics, ed. J. R. Rumble, CRC Press/Taylor & Francis, Boca Raton, FL, 106th edn, 2025

[cit78] Schafer C., Ringstrom R., Hanrieder J., Rahm M., Albinsson B. (2024). Nat. Commun..

[cit79] Gilbert A. and Baggott J., Essentials of Molecular Photochemistry, Blackwell Scientific Publications, 1991

[cit80] Kaiser T. E., Wang H., Stepanenko V., Würthner F. (2007). Angew. Chem., Int. Ed..

[cit81] Kaiser T. E., Stepanenko V., Würthner F. (2009). J. Am. Chem. Soc..

[cit82] Piwonski H., Nozue S., Fujita H., Michinobu T., Habuchi S. (2021). Nano Lett..

[cit83] Barotov U., Arachchi D. H. T., Klein M. D., Zhang J., Sverko T., Bawendi M. G. (2023). Adv. Opt. Mater..

[cit84] Cardeynaels T., Paredis S., Danos A., Harrison A. (2021). Dyes Pigm..

[cit85] Gu P., He T., Wang Z., Wang S. (2024). Chem. Sci..

[cit86] Richard C. A., Pan Z., Hsu H. Y., Cekli S. (2014). ACS Appl. Mater. Interfaces.

[cit87] He Y., Okamoto N., Maeda T., Nakazumi H. (2017). J. Jpn. Soc. Colour Mater..

[cit88] Richard C. A., Pan Z., Parthasarathy A., Arroyave F. A. (2014). J. Mater. Chem. A.

[cit89] Birks J. B. , Photophysics of Aromatic Molecules, Wiley-Interscience, 1970

[cit90] Würthner F., Kaiser T. E., Saha-Möller C. R. (2011). Angew. Chem., Int. Ed..

[cit91] Liu Q., Vandewal K. (2023). Adv. Mater..

[cit92] Bi P., Zhang S., Chen Z., Xu Y. (2021). Joule.

[cit93] Dance Z. E., Mickley S. M., Wilson T. M. (2008). J. Phys. Chem. A.

[cit94] Dong Y., Sukhanov A. A., Zhao J. (2019). J. Phys. Chem. C.

[cit95] Hussain M., El-Zohry A. M., Hou Y. (2021). J. Phys. Chem. B.

[cit96] Medina Rivero S., Alonso-Navarro M. J., Tonnele C., Marin-Beloqui J. M. (2023). J. Am. Chem. Soc..

[cit97] Lefler K. M., Brown K. E., Salamant W. A. (2013). J. Phys. Chem. A.

[cit98] Hirata Y., Tanaka I. (1976). Chem. Phys. Lett..

[cit99] El-Sayed M. A. (1963). J. Chem. Phys..

[cit100] Baba M. (2011). J. Phys. Chem. A.

[cit101] Baumgärtner K., Hofmann M., Rominger F., Elbert S. M., Dreuw A., Mastalerz M. (2020). J. Org. Chem..

[cit102] Preda G., Mobili R., Ravelli D., Amendola V., Pasini D. (2024). J. Org. Chem..

[cit103] Lei P., Zhang S., Zhang N., Yin X., Wang N., Chen P. (2020). ACS Omega.

[cit104] Nakazawa T., Murata I. (1977). J. Am. Chem. Soc..

[cit105] Murata I. (1983). Pure Appl. Chem..

[cit106] Zhang Y., Zhang D., Huang T. (2021). Angew. Chem., Int. Ed..

[cit107] Friedman H. C., Cosco E. D., Atallah T. L. (2021). Chem.

[cit108] Tsai Y.-C., Chen Y.-C., Liu H.-F. (2025). J. Am. Chem. Soc..

[cit109] Lowrie W., Westbrook R. J. E., Guo J. (2023). J. Chem. Phys..

[cit110] Coles S. J. , Allan D. R. and Beavers C. M., Structure and Bonding, Springer, 2020, vol. 5, pp. 69–140

[cit111] (a) CCDC 2495341: Experimental Crystal Structure Determination, 2026, 10.5517/ccdc.csd.cc2prlwy

