# Supplementary material for: Homoconjugation-induced enhancements of photophysical properties in donor–acceptor triptycenes arise from interplay between intramolecular charge transfer and exciton states
Source: Chem Sci. 2026 Jun 8;17(30):14590–602. doi: 10.1039/d6sc01073c (PMC13280452; doi:10.1039/d6sc01073c)
Supplement: Supplementary file 1 [file SC-017-D6SC01073C-s001.pdf]

Supplementary Information (SI) for Chemical Science.
This journal is © The Royal Society of Chemistry 2026

Homoconjugation-Induced Enhancements of Photophysical Properties in Donor-Acceptor Triptycenes Arise from Interplay between Intramolecular Charge Transfer and Exciton States

Stefan Warrington,[a,b] Hristo Ivov Gonev,[c] Gary S. Nichol,[a] Eleanor M. Dodd,[d] Simon J. Coles,[d] Thomas J. Penfold,[e] Marc K. Etherington,[f] Tracey M. Clarke[c]* and Iain A. Wright[a]*

a. EaStCHEM, School of Chemistry, University of Edinburgh, Joseph Black Building, David Brewster Road, Edinburgh, EH9 3FJ, UK.
b. Department of Chemistry, Loughborough University, Epinal Way, Loughborough, Leicestershire, LE11 3TU, UK.
c. Department of Chemistry, University College London, Christopher Ingold Building, London, WC1H 0AJ, UK.
d. EPSRC Crystallographic Service, Department of Chemistry, University of Southampton, Highfield, Southampton, SO17 1BJ, UK.
e. Chemistry, School of Natural and Environmental Sciences, Newcastle University, Newcastle upon Tyne, NE1 7RU, UK.
f. School of Engineering, Physics and Mathematics, Northumbria University, Ellison Place, Newcastle upon Tyne, NE1 8ST UK.

Contents

**General Experimental**	**S3**
**Synthetic Procedures**	**S4**
**NMR Spectra of All New Compounds**	**S17**
**Mass Spectrometry of All New Compounds**	**S25**
**X-Ray Crystallography**	**S29**
**Computational**	**S30**
**Electrochemistry**	**S32**
**Vibrational Modes Analysis**	**S34**
**UV/Vis and Beer-Lambert Plots**	**S35**
**Solvatochromism**	**S39**
**Time-Resolved Photophysics**	**S41**
**References**	**S44**

General Experimental

Unless otherwise noted, all starting materials and reagents were purchased from commercial chemical suppliers Fluorochem, TCI, Merck and were used without further purification. Anhydrous solvents were purified with an 'Inert' solvent purification system (Model PS-MD-5/7). Thin layer chromatography (TLC) was performed on silica gel 60 F*254* (Supelco). Column chromatography was performed using silica gel (Fluorochem, 60A 40-63u).

Nuclear magnetic resonance (NMR) spectroscopy. NMR spectra were recorded at 298 K, unless otherwise stated, using a Bruker Ava 500 MHz spectrometer. ^1H and ^{13}C spectra were recorded at 400/500 MHz and 101/126 MHz respectively. Chemical shifts (δ) are reported in parts per million (ppm) relative to residual $CDCl_3$, and J values are given in Hertz (Hz). Abbreviations for multiplets are singlet (s), doublet (d), triplet (t), quartet (q), doublet of doublets (dd), doublet of triplets (dt) and multiplet (m).

Low Resolution Mass Spectrometry (LRMS). LRMS were carried out using a Waters RADIAN ASAP Direct Mass Detector.

High Resolution Mass Spectrometry (HRMS). HRMS were carried out using a Bruker Solarix XR (MALDI) and a Thermofisher LTQ Orbitrap XL (Nanospray Ionisation).

Melting points (Mp.). Melting points were obtained using a Stuart Scientific SMP3 Melting Point Apparatus and were not corrected.

Steady state absorption and emission spectroscopy. UV/Vis measurements were carried out on Shimadzu UV1800 and SolidSpec 3700i spectrophotometers and emission on a HORIBA Fluoromax 4 and Fluorolog-QM. For the time-correlated single photon counting (TCSPC) measurements, a 502 nm diode was used to excite the solutions, which were probed at the wavelength at which they exhibit maximum steady-state emission signal.

Cyclic voltammetry. All electrochemistry was recorded using a PGSTAT12 Autolab electrochemical workstation. DCM was used as a solvent with an analyte molarity of ca. 10^{-4} M in the presence of 10^{-1} M $(n\text{-}Bu_4N)(PF_6)$ as a supporting electrolyte. Solutions were degassed with Ar and experiments run under a blanket of Ar. A glassy carbon disk, Pt wire and Ag/Ag^+ ($AgNO_3$ in acetonitrile) were used as the working, counter, and reference electrodes respectively. All samples were referenced to an internal or external Fc/Fc^+ redox couple.

Synthetic Procedures

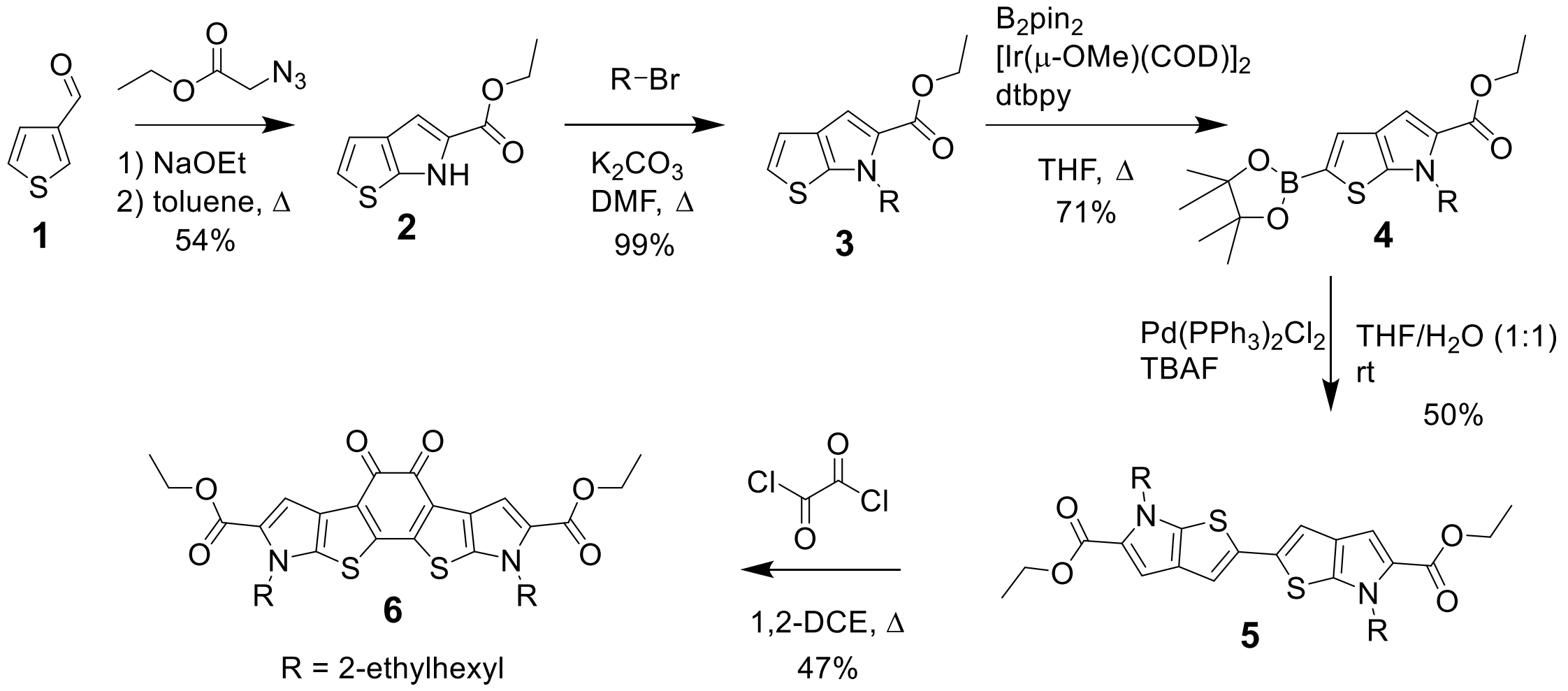

Scheme S1. Synthesis of diketone **6**.

Compound 2

Synthesised according to a modified version of a procedure by Vogt et al.[S1] Under a blanket of argon, a solution of NaOEt (2.95 g, 43.4 mmol) in EtOH (100 mL) was cooled to −20°C. To this stirred solution thiopene-3-carboxaldehyde (0.95 mL, 10.8 mmol) and ethyl azido acetate (5.0 mL, 43.4 mmol) were added simultaneously and dropwise over the course of 3 hours. After addition the mixture was left to stir at −20 °C for 1 hour then allowed to warm to room temperature overnight. The mixture was quenched with water (100 mL) and then extracted into diethyl ether (3 × 200 mL). The combined organic fractions were dried over $MgSO_4$ and concentrated under reduced pressure. The residue was then dissolved in toluene (150 mL) and refluxed for 2 hours with monitoring by TLC. Once the conversion was complete, the toluene was removed under reduced pressure and the residue dissolved in a small amount of dichloromethane (DCM) and passed through a short plug of silica gel (SiO_2) which was washed with further DCM. The solvent was removed and the crude product recrystallised from a hexane/DCM mixture to give **2** (1.3 g, 62%) as large colourless crystals.

NMR spectra obtained were in agreement with the literature.[S1]

^{1}H NMR (400 MHz, $CDCl_3$) δ 7.87 (ddd, *J* = 2.9, 1.1, 0.5 Hz, 1H), 7.49 (dd, *J* = 5.1, 1.2 Hz, 1H), 7.31 (dd, *J* = 5.1, 3.0 Hz, 1H), 6.96 (s, 1H), 4.36 (q, *J* = 7.1 Hz, 2H), 1.39 (t, *J* = 7.1 Hz, 3H).

^{13}C NMR (126 MHz, $CDCl_3$) δ 161.7, 138.2, 131.6, 128.2, 120.6, 117.9, 107.4, 60.7, 14.4.

LRMS (ASAP, *m/z*) 196.24 [M+H$^+$] requires 196.04 for [$C_9H_{10}NO_2S^+$].

Compound 3

Under a blanket of argon, **2** (4.47 g, 22.9 mmol) was dissolved in anhydrous DMF (40 mL). K_2CO_3 (6.33g, 45.8 mmol) was added followed by 2-ethylhexyl bromide (5.92 mL, 34.3 mmol) and the stirred mixture was heated to reflux overnight. The mixture was cooled to room temperature and water (200 mL) was added followed by diethyl ether (200 mL). The aqueous layer was removed and the organic layer was then successively washed with more water (5 × 200 mL) and finally brine (200 mL). The organic phase was dried over $MgSO_4$, drying agent filtered, and the mixture concentrated under reduced pressure. The purification was achieved by column chromatography (SiO_2; hexanes:ethyl acetate, 1:0 – 9:1) yielding **3** (7.01g, 99%) as a colourless oil.

^{1}H NMR (500 MHz, $CDCl_3$) δ 7.18 (s, 1H), 6.97 (d, *J* = 5.3 Hz, 1H), 6.89 (d, *J* = 5.4 Hz, 1H), 4.36 – 4.30 (m, 4H), 2.11 – 2.00 (m, 1H), 1.38 (t, *J* = 7.1 Hz, 3H), 1.36 – 1.24 (m, 8H), 0.88 (dd, *J* = 15.3, 7.4 Hz, 6H).

^{13}C NMR (126 MHz, $CDCl_3$) δ 161.4, 142.8, 128.1, 127.5, 120.0, 118.2, 109.5, 60.0, 52.9, 39.6, 30.4, 28.4, 23.8, 23.0, 14.4, 14.0, 10.6.

HRMS (MALDI, m/z) 308.1678 [M+H$^+$], requires 308.1679 for [$C_{17}H_{26}NO_2S^+$].

Compound 4

Under a blanket of argon, **3** (7.0 g, 22.8 mmol) and bis(pinacolato)diboron (11.6 g, 45.5 mmol) were dissolved in tetrahydrofuran (THF, 150 mL). The solution was degassed for one hour with argon. 4,4'-Di-*tert*-butyl-2,2'-bipyridine (dtbpy, 0.182 g, 0.680 mmol) and [Ir(μ-OMe)COD]$_2$ (0.453 g, 0.680 mmol) were added and the stirred mixture was heated to reflux overnight. The mixture was then cooled to room temperature and the solvent removed under reduced pressure. The residue was purified by column chromatography (SiO_2; hexanes:ethyl acetate; 1:0 increasing to 9:1) yielding **4** (7.0 g, 71%) as a colourless oil.

^{1}H NMR (400 MHz, $CDCl_3$) δ 7.53 (s, 1H), 7.19 (s, 1H), 4.35 – 4.28 (m, *J* = 17.7, 10.8, 5.7 Hz, 4H), 2.09 – 2.00 (m, 1H), 1.35 (s, 12H), 1.31 – 1.20 (m, 11H), 0.87 – 0.83 (m, 6H).

^{13}C NMR (101 MHz, $CDCl_3$) 161.5, 131.0, 130.3, 129.8, 109.8, 100.1, 84.2, 60.2, 53.0, 39.5, 30.6, 28.6, 24.9, 24.9, 23.9, 23.2, 14.5, 14.1, 10.7.

HRMS (MALDI, m/z) 456.2347 [M+Na$^+$], requires 456.2256 for [$C_{23}H_{36}BNO_4SNa^+$]

Compound 5

Under a blanket of argon, **4** (897 mg, 2.07 mmol) was dissolved in THF (20 mL) and water (5 mL) was added. The mixture was degassed for one hour by sparging with argon. $Pd(PPh_3)_2Cl_2$ (145 mg, 0.207 mmol) was added followed by the slow addition of a solution of 1.0 M tetrabutylammonium fluoride (TBAF) solution in THF (3.10 mL, 3.10 mmol) and the mixture was stirred at room temperature overnight. The resulting solution was passed through a pad of celite and then extracted into $CHCl_3$ (200 mL) and washed with water (100 mL). The organic portion was dried with $MgSO_4$, the drying agent removed by filtration and the solvent concentrated under reduced pressure. The residue was then purified using column chromatography (SiO_2; hexanes:DCM; 1:1) yielding **5** (544 mg, 85%) colourless oil.

^{1}H NMR (400 MHz, $CDCl_3$) δ 7.11 (s, 2H), 7.00 (s, 2H), 4.34 – 4.28 (m, 8H), 2.04 (dt, *J* = 13.1, 6.6 Hz, 2H), 1.37 (t, *J* = 7.1 Hz, 6H), 1.35 – 1.23 (m, 16H), 0.88 (m, 12H).

^{13}C NMR (101 MHz, $CDCl_3$) δ 161.2, 141.5, 133.1, 128.3, 127.4, 114.2, 109.9, 60.1, 53.1, 39.8, 30.6, 28.5, 23.9, 23.1, 14.6, 14.1, 10.7.

HRMS (MALDI, m/z) 612.3066 [M^+], requires 612.3050 for [$C_{34}H_{48}N_2O_4S_2^+$]

Compound 6

Under a blanket of argon, **5** (268 mg, 0.440 mmol) was dissolved in anhydrous 1,2-dichloroethane (DCE, 10 mL). Oxalyl chloride (0.060 mL, 0.660 mmol) was added, and the stirred mixture was heated to reflux overnight. The dark green reaction mixture was then cooled to room temperature and a small volume of water was added to quench. The mixture was diluted in chloroform (250 mL) and washed with water (2 × 150 mL) the organic fractions were dried over $MgSO_4$, the drying agent removed by filtration and the organic solvent concentrated under reduced pressure. The dark residue was then recrystallised from ethyl acetate yielding **6** (250 mg, 85%) as a dark green solid. M.p. 197–200°C.

^{1}HNMR (500 MHz, $CDCl_3$) δ 7.40 (s, 2H), 4.44 – 3.91 (m, 8H), 2.07 – 1.87 (m, 2H), 1.39 (t, *J* = 7.1 Hz, 6H), 1.36 – 1.19 (m, 12H), 0.93 – 0.80 (m, 16H).

^{13}C NMR (126 MHz, $CDCl_3$) 174.4, 160.8, 141.7, 139.7, 129.9, 126.4, 125.4, 110.6, 60.7, 53.1, 40.0, 30.6, 28.5, 23.9, 23.1, 14.5, 14.4, 14.1, 10.7.

HRMS (NSI, m/z) 667.2869 [$M+H^+$], requires 667.2870 for [$C_{36}H_{47}N_2O_6S_2^+$]

N.B.: Differences in the stereochemistry of the branched alkyl chains result in one of the ^{13}C resonances in this compound appearing as two extremely close peaks at 14.5 and 14.4 ppm.

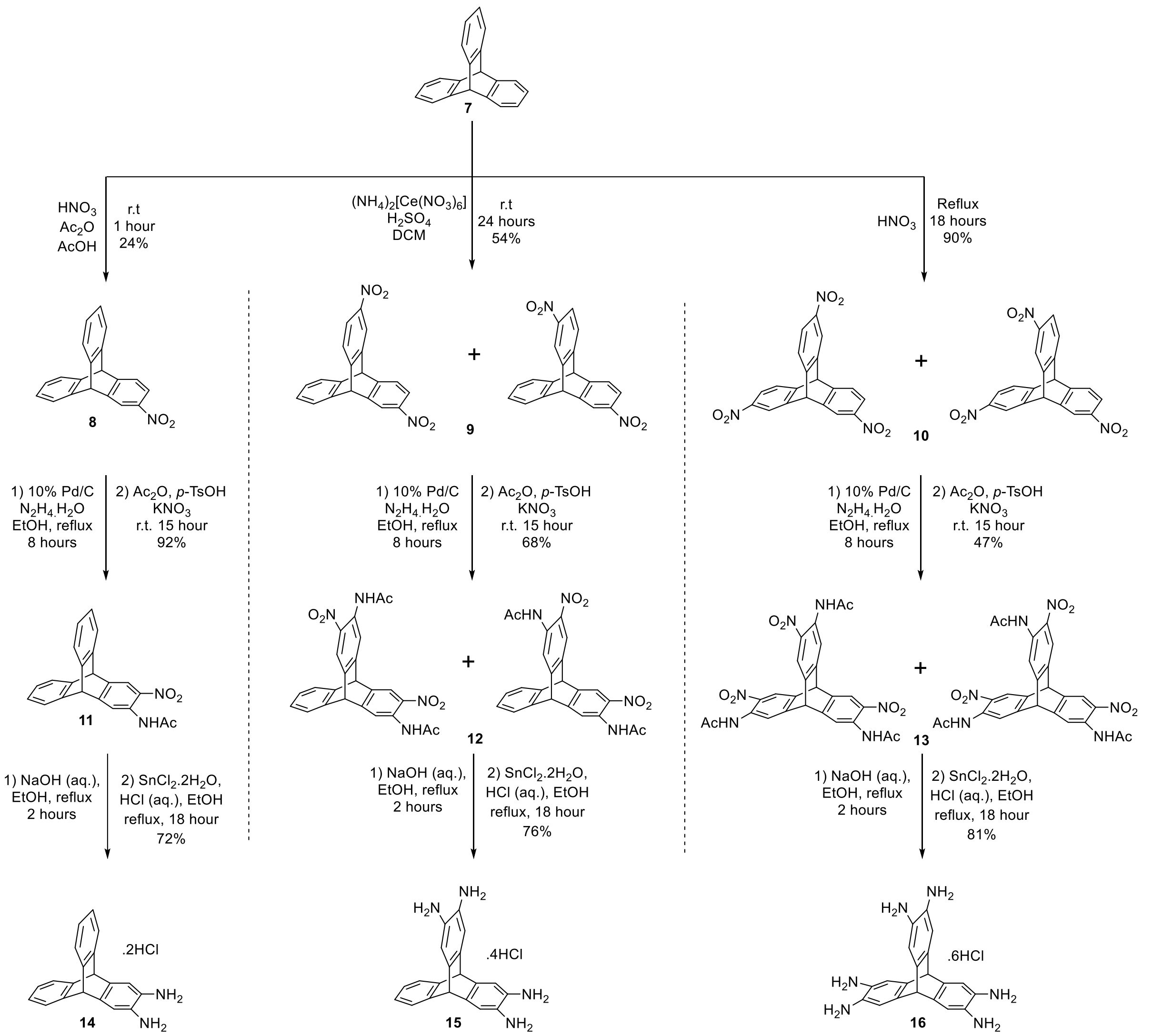

Scheme S2. Synthesis of amino triptycenes, compound **14**, **15** and **16**.

Compound 8

Synthesised according to the procedure of Chong et al.[S2] Under a blanket of argon, triptycene (2.70 g, 10.5 mmol) was dispersed in a combination of AcOH (100%, 50 mL), HNO_3 (69%, 1 mL) and Ac_2O (10 mL) and the mixture refluxed for one hour. The mixture was cooled to room temperature, then poured over ice forming a white cloudy precipitate. The precipitate was collected by vacuum filtration and washed with water until the washings were no longer acidic. The product was finally washed with a small volume of MeOH yielding **8** (0.750 g, 24%) as a white solid.

NMR spectra obtained were in agreement with the literature.[S2]

^{1}H NMR (400 MHz, $CDCl_3$) δ 8.22 (d, *J* = 2.2 Hz, 1H), 7.94 (dd, *J* = 8.1, 2.2 Hz, 1H), 7.50 (d, *J* = 8.1 Hz, 1H), 7.45 – 7.40 (m, 4H), 7.08 – 7.02 (m, 4H), 5.55 (s, 1H), 5.54 (s, 1H).

^{13}C NMR (126 MHz, $CDCl_3$) δ 152.6, 147.2, 145.5, 144.1, 143.6, 126.0, 125.9, 124.6, 124.2, 124.1, 121.6, 118.8, 54.1, 53.9.

LRMS (ASAP, *m/z*) 299.15 [M+] requires 299.09 for [$C_{20}H_{13}NO_2$].

Compound 9

Synthesised according to the procedure of White et al.[S3] Under a blanket of argon, triptycene (1.50 g, 5.89 mmol) was dissolved in DCM (25 mL). Ceric ammonium nitrate (6.46 g, 11.79 mmol) was added to the mixture followed by the dropwise addition of H_2SO_4 (1.10 mL, 21.2 mmol). After stirring at room temperature for 24 hours a turbid green suspension resulted, the mixture was then filtered and the solids were washed with DCM (2 × 100 mL). The organic filtrate was transferred to a separatory funnel and washed with aqueous K_2CO_3 (10 % solution, 100 mL) followed by brine (100 mL). The organic phase was dried using $MgSO_4$, the drying agent removed by filtration and the solvent removed under reduced pressure. Column chromatography (SiO_2; petroleum ether 40-60 °C:DCM; 2:1 increasing to 1:1) was used to isolate a mixture of the 2,6- and the 2,7-dinitrotriptycene isomers **9** (1.1 g, 54%) as a white solid. As both isomers are intermediates towards the desired tetraamine **15**, the mixture was used without further separation.

NMR spectra obtained were in agreement with the literature.[S4]

^{1}H NMR (500 MHz, $CDCl_3$) δ 8.28 – 8.26 (m, 2H), 8.01 – 7.97 (m, 2H), 7.58 – 7.54 (m, 2H), 7.49 – 7.44 (m, 2H), 7.12 – 7.09 (m, 2H), 5.67 (s,2H).

LRMS (ASAP, *m/z*) 345.24 [$M+H^+$] requires 345.09 for [$C_{20}H_{13}N_2O_4^+$].

Compound 10

Synthesised according to the procedure of Chong et al.[S2] Triptycene (1.00 g, 3.93 mmol) was dispersed in HNO_3 (69%, 20 mL) and refluxed overnight. After 18 hours the mixture was cooled to room temperature, added to a large excess of ice water resulting in a white precipitate forming. The precipitate was filtered and washed with copious water until the washing were no longer acidic. The precipitate was finally washed with minimal methanol furnishing a mixture of the 2,6,14- and the 2,7,14-trinitrotriptycene regioisomers **10** (1.64 g, 90%) as a white solid. As both isomers are intermediates towards the desired hexaamine **16**, the mixture was used without further separation.

NMR spectra obtained were in agreement with the literature.[S5]

^{1}H NMR (400 MHz, $CDCl_3$) δ 8.35 – 8.31 (m, 3H), 8.07 – 8.03 (m, 3H), 7.66 – 7.60 (m, 3H), 5.86 – 5.80 (m, 2H).

LRMS (ASAP, *m/z*) 389.27 [M^+] requires 389.06 for [$C_{20}H_{11}N_3O_6^+$].

General procedure for **11**, **12**, **13**

Step 1) Under a blanket of argon, nitrated triptycenes (1 eq. per NO_2 group) were dispersed in EtOH and the mixture cooled to 0 °C. To the cold solution, palladium 10% on charcoal (Pd/C) was added carefully followed by the dropwise addition of $N_2H_4.H_2O$. The mixture was then heated to reflux for 18 hours. The reaction was then cooled to room temperature and filtered over a pad of celite to remove the Pd/C. The celite washed with EtOH and the resulting filtrate solution was concentrated under reduced pressure.

Step 2) The residue was then suspended in Ac_2O and stirred for 30 minutes. To the stirring solution, *p*-toluene sulfonic acid (*p*-TsOH, 1.2 eq. per NO_2 group) and KNO_3 (1.1 eq. per NO_2 group) were added, and the reactions were allowed to stir at room temperature overnight. Once the reaction was complete the mixture was added to an excess of water and the solution stirrer for one hour until a precipitate formed. The resulting precipitates were filtered under *vacuo* and washed with water until neutral. The precipitates were dissolved in DCM and $MgSO_4$ added, the drying agent then removed by filtration. The resulting materials were purified, details given below, yielding **11** (0.290 g, 92%), **12** (1.20 g, 68%), **13** (1.10 g, 47%) as yellow/mustard solids.

Compound 11

The general procedure was followed using **8** (270 mg, 0.902 mmol), Pd/C (10% on charcoal, 20 mg), and $N_2H_4.H_2O$ (0.2 mL, 4.13 mmol) in EtOH (15 mL) in Step 1, then *p*-TsOH (190 mg, 0.992 mmol) and KNO_3 (95 mg, 0.947 mmol) in Ac_2O (10 mL) in Step 2. Purification achieved by adding a mixture of petroleum ether (boiling range 40-60 °C) and EtOH to the concentrated residue, leading to the formation of a bright yellow precipitate. The resulting solids were collected by vacuum filtration and washed with EtOH. Yielding **11** (0.290 g, 92 %) as a bright yellow solid.

NMR spectra obtained were in agreement with the literature.[S2]

^1H NMR (400 MHz, $CDCl_3$) δ 10.51 (s, 1H), 8.89 (s, 1H), 8.18 (s, 1H), 7.48 – 7.34 (m, 4H), 7.12 – 6.99 (m, 4H), 5.53 (s, 1H), 5.45 (s, 1H), 2.25 (s, 3H).

^{13}C NMR (101 MHz, $CDCl_3$) δ 169.2, 153.8, 144.0, 143.2, 140.3, 133.8, 132.7, 126.0, 126.0, 124.4, 123.9, 120.2, 117.1, 54.1, 53.0, 25.8.

LRMS (ASAP, *m/z*) 357.35 [$M+H^+$] requires 357.12 for [$C_{22}H_{17}N_2O_3^+$].

Compound 12

The general procedure was followed using **9** (1.5 g, 3.85 mmol), Pd/C (10% on charcoal,150 mg), and $N_2H_4.H_2O$ (1.5 mL, 30.9 mmol) in EtOH (50 mL) in Step 1, then *p*-TsOH (1.63 g, 8.47 mmol) and KNO_3 (817 mg, 8.08 mmol) in Ac_2O (50 mL) in Step 2. Purification was achieved using column chromatography (SiO_2; DCM:MeOH; 99:1 increasing to 96:4). Followed by trituration from DCM/MeOH yielding **12** (1.20 g, 68 %) as a mustard solid comprised of a mixture of the two possible isomers. As both isomers are intermediates towards the desired tetraamine **16**, the mixture was used without further separation.

NMR spectra obtained were in agreement with the literature.[S3]

^{1}H NMR (500 MHz, $CDCl_3$) δ 10.49 – 10.45 (m, 2H), 8.93 – 8.92 (m, 2H), 8.21 – 8.19 (m, 2H), 7.45 – 7.40 (m, 2H), 7.11 – 7.09 (m, 2H), 5.63 – 5.48 (m, 2H), 2.27 – 2.24 (m, 6H).

LRMS (ASAP, *m/z*) 459.40 [$M+H^+$] requires 459.13 for [$C_{24}H_{19}N_4O_6^+$].

Compound 13

The general procedure was followed using **10** (1.5 g, 3.83 mmol), Pd/C (10% on charcoal, 200 mg), and $N_2H_4.H_2O$ (1.16 mL, 22.96 mmol) in EtOH (30 mL) in Step 1, then *p*-TsOH (2.43 g, 12.63 mmol) and KNO_3 (1.24 g, 12.26 mmol) in Ac_2O (20 mL) in Step 2. Purification was achieved using column chromatography (SiO_2; DCM:MeOH; 99:1 increasing to 95:5). Followed by precipitation from DCM/MeOH yielding **13** (1.10 g, 47 %) as a mustard solid comprised of a mixture of the two possible isomers. As both isomers are intermediates towards the desired hexaamine **16**, the mixture was used without further separation.

NMR spectra obtained were in agreement with the literature.[S2,S6]

^{1}H NMR (400 MHz, $CDCl_3$) δ 10.49 – 10.43 (m, 2H), 8.99 – 8.96 (m, 2H), 8.28 – 8.23 (m, 2H), 5.74 – 5.52 (m, 2H), 1.55 (s, 6H).

LRMS (ASAP, *m/z*) 561.34 [$M+H^+$] requires 561.14 for [$C_{26}H_{21}N_6O_9^+$].

General procedure for **14**, **15**, **16**

Step 1) Under a blanket of argon, **11**, **12** or **13** was dispersed in a mixture of EtOH and water (1:0.1, v/v) and NaOH added (10 eq per acetamide). The reaction was heated to reflux for 2 hours then allowed to cool to room temperature and the solvent removed under reduced pressure. A small volume of water is added to the concentrated residue forming a solid product which was collected by vacuum filtration and washed with a small volume of water then allowed to dry under a stream of air and used immediately.

Step 2) Under an argon atmosphere, the solids were dissolved in a 2:1 mixture (v/v) of EtOH and concentrated HCl (37.5 % aqueous) and $SnCl_2.H_2O$ (20 eq) are added. The mixtures were then heated to reflux for at least 18 hours. Once complete the reactions were cooled to room temperature and the solvent removed under reduced pressure. Concentrated HCl was added to the residues and the suspension heated to reflux for approximately 15 minutes. The resulting hydrochloride salts were then filtered and washed with further small volumes of conc. HCl to isolate the products as off-white solids.

Compound 14

The general procedure was followed using **11** (110 mg, 0.31 mmol), NaOH (123 mg, 3.1 mmol), in EtOH (5.0 mL) and H_2O (0.5 mL) in Step 1, then $SnCl_2.H_2O$ (677 mg, 3.26 mmol) in EtOH (5 mL) and HCl (2.5 mL) working up with conc. HCl (5 mL) in Step 2 yielding **14** (110 mg, 72%),

NMR spectra obtained were in agreement with the literature.[S7]

^{1}H NMR (400 MHz, d6-DMSO) δ 7.43 – 7.38 (m, 4H), 7.06 (s, 2H), 7.01 – 6.96 (m, 4H), 5.51 (s, 2H).

^{13}C NMR (101 MHz, d6-DMSO) δ 145.2, 124.9, 123.4, 51.7.

LRMS (ASAP, *m/z*) 285.20 [M+H$^+$] requires 285.14 for [$C_{20}H_{17}N_2^+$].

Compound 15

The general procedure was followed using **12** (500 mg, 1.02 mmol), NaOH aq. (10%, 15 mL), in EtOH (1.5 mL) in Step 1, then $SnCl_2.H_2O$ (2.6 g, 13.9 mmol) in EtOH (10 mL) and HCl (5 mL) working up with conc. HCl (7 mL) in Step 2 yielding **15** (358 mg, 76%).

NMR spectra obtained were in agreement with the literature.[S3]

^{1}H NMR (500 MHz, d4-MeOD) δ 7.44 – 7.42 (m, 2H), 7.39 (s, 4H), 7.04 – 7.02 (m, 2H), 5.62 (s, 2H).

^{13}C NMR (126 MHz, d4-MeOD) 145.2, 126.8, 125.4, 124.9, 119.4 53.3.

LRMS (ESI, *m/z*) 315.16 [M+ H$^+$] requires 315.16 for [$C_{20}H_{19}N_4^+$].

Compound 16

The general procedure was followed using **13** (516 mg, 0.92 mmol), NaOH aq. (10%, 10 mL), in EtOH (10 mL) in Step 1, then $SnCl_2.H_2O$ (3.5 g, 16.9 mmol) in EtOH (10 mL) and HCl (5 mL) working up with conc. HCl (5 mL) in Step 2 yielding **16** (530 mg, 81%).

NMR spectra obtained were in agreement with the literature.[S2,S8]

^1H NMR (400 MHz, d6-DMSO) δ 7.03 (s, 6H), 5.30 (s, 2H)

^1H NMR (500 MHz, d4-MeOD) δ 7.26 (s, 6H), 5.47 (s, 2H).

^{13}C NMR (126 MHz, d4-MeOD) δ 143.4, 126.3, 118.3, 52.5.

LRMS (ESI, *m/z*) 344.17 [M^+] requires 344.17 for [$C_{20}H_{20}N_6^+$].

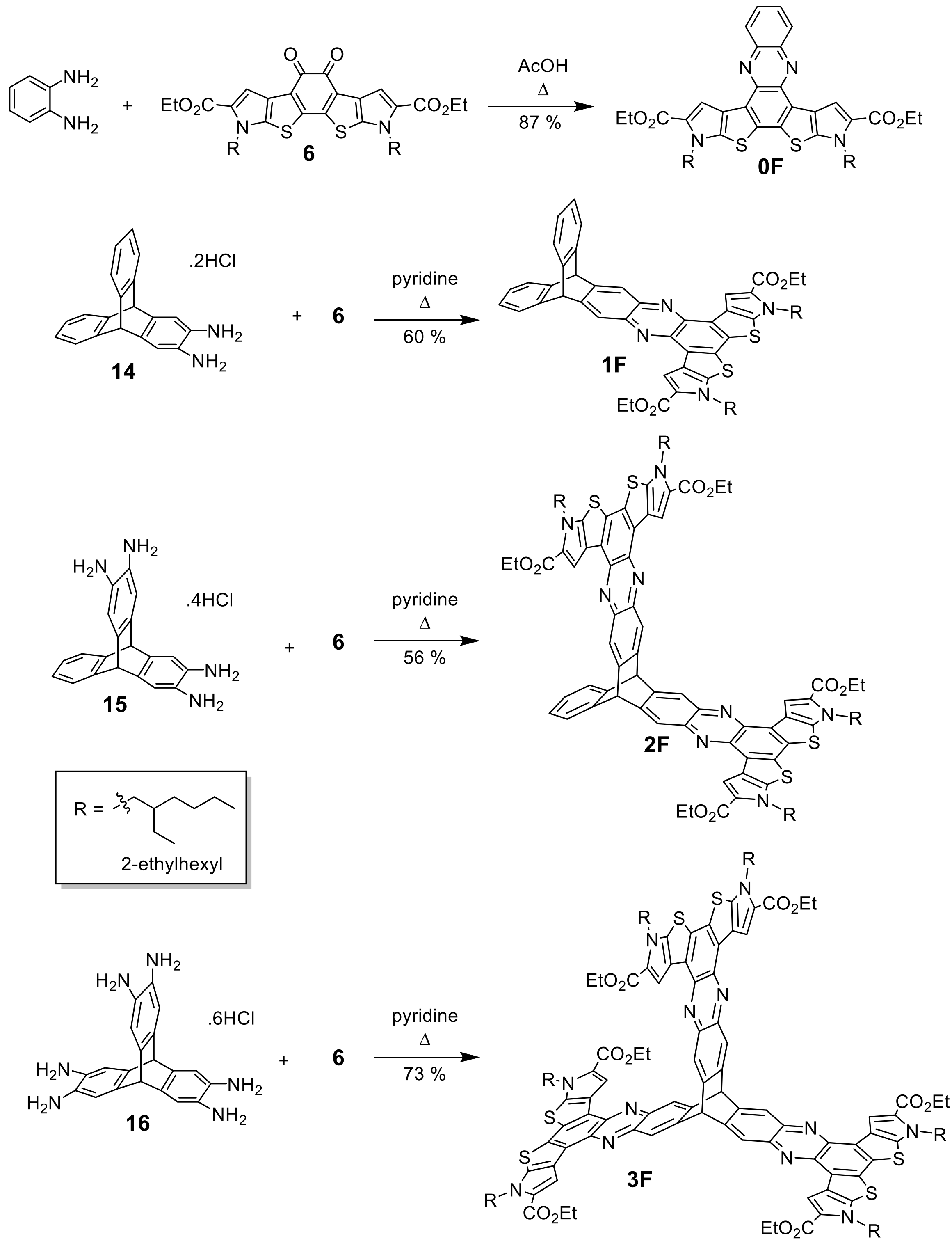

Scheme S3. Synthesis of compounds **0F**-**3F**.

0F

Under a blanket of argon, **6** (150 mg, 0.22 mmol) and *o*-phenylenediamine (100 mg, 0.92 mmol) were dispersed in AcOH (30 mL) and the mixture was degassed with argon for one hour then heated to reflux with stirring overnight, then cooled to room temperature. Once cool the red turbid solution was poured into a beaker of ice water (150 mL) generating a deep red precipitate. The mixture was stirred for 30 minutes and allowed to stand until all solids had settled. The bulk of the water was decanted, and the red solid was collected by filtration and washed with a small volume of methanol and subsequently dried under a stream of air. The product was then purified by column chromatography (SiO_2; $CHCl_3$) followed by precipitation from $CHCl_3$ with MeOH yielding **0F** (144 mg, 87%) as a dark red solid. M.p. 198–200°C.

Single crystals suitable for X-ray diffraction were grown using vapour diffusion of hexane into $CHCl_3$.

^{1}H NMR (400 MHz, $CDCl_3$) δ 8.42 – 8.31 (m, 2H), 7.97 (s, 2H), 7.88 – 7.82 (m, 2H), 4.48 – 4.35 (m, 8H), 2.14 – 2.00 (m, 2H), 1.50 (t, *J* = 7.1 Hz, 6H), 1.39 – 1.24 (m, 16H), 0.93 – 0.84 (m, 12H).

^{13}C NMR (101 MHz, $CDCl_3$) δ 161.3, 142.0, 139.6, 139.2, 133.4, 130.0, 129.4, 127.9, 125.5, 125.4, 112.6, 60.3, 53.0, 40.0, 30.7, 28.65, 28.62, 24.0, 23.1, 14.7, 14.1, 10.8.

HRMS (NSI, *m/z*) 739.3346 [M+H$^+$] (requires 739.3346 for $C_{42}H_{51}O_4N_4S_2^+$).

N.B.: Differences in the stereochemistry of the branched alkyl chains result in one of the ^{13}C resonances in this compound appearing as two extremely close peaks at 28.65 and 28.62 ppm

1F

Under a blanket of argon, **6** (150 mg, 0.22 mmol) and **14** (77 mg, 0.26 mmol) were dissolved in anhydrous pyridine (10 mL). The mixture was degassed by sparging with argon for 1 hour, then heated to reflux with stirring overnight. The red mixture was allowed to cool to room temperature and then poured directly into a beaker of stirring MeOH (150 mL). This resulted in the formation of an orange-red precipitate which was stirred for 30 minutes. The orange precipitate was then collected by filtration and washed with MeOH the dried under a stream of air. The purification of the material was achieved by column chromatography (SiO_2; $CHCl_3$) followed by precipitation from $CHCl_3$ with MeOH yielding **1F** (109 mg, 60%) as a bright orange solid. M.p. 265–267 °C.

Single dark red prism-shaped crystals were grown by slow evaporation of solution in a mixture of DCM and MeOH (approximate ratio 1:1 v/v).

^{1}H NMR (400 MHz, $CDCl_3$) δ 8.29 (s, 2H), 8.10 (s, 2H), 7.53 (dd, *J* = 5.4, 3.2 Hz, 4H), 7.12 (dd, *J* = 5.5, 3.1 Hz, 4H), 5.75 (s, 2H), 4.50 – 4.36 (m, 8H), 2.11 – 2.09 (m, 2H), 1.50 (t, *J* = 7.1 Hz, 6H), 1.40 – 1.24 (m, 16H), 0.93 – 0.84 (m, 12H).

^{13}C NMR (101 MHz, $CDCl_3$) δ 161.3, 145.8, 143.8, 141.3, 139.7, 138.9, 133.1, 127.9, 126.2, 125.7, 125.6, 124.2, 123.1, 112.8, 60.3, 53.8, 53.1, 40.0, 30.6, 28.6, 24.0, 23.1, 14.7, 14.1, 10.8.

HRMS (MALDI, *m/z*) 915.4001 [M+H$^+$] (requires 915.3972 for $C_{56}H_{59}O_4N_4S_2^+$).

2F

Under a blanket of argon, **6** (250 mg, 0.370 mmol) and **15** (78 mg, 0.17 mmol) were dissolved in anhydrous pyridine (20 mL). The mixture was degassed by sparging with argon for 1 hour, then heated to reflux with stirring overnight. The red mixture was allowed to cool to room temperature and then poured directly into a beaker of stirring MeOH (200 mL) generating a bright red-orange precipitate. The precipitate was then collected by filtration and washed with MeOH the dried under a stream of air. The purification of the material was achieved by column chromatography (SiO_2; $CHCl_3$) followed by precipitation from $CHCl_3$ with MeOH yielding **2F** (150 mg, 56%) as an orange-red solid. M.p. >300°C.

^{1}H NMR (400 MHz, $CDCl_3$) δ 8.46 (s, 4H), 8.09 (s, 4H), 7.78 – 7.64 (m, 2H), 7.29 – 7.20 (m, 2H), 6.11 (s, 2H), 4.49 – 4.29 (m, 16H), 2.07 (dt, *J* = 12.2, 6.1 Hz, 4H), 1.52 (t, *J* = 7.1 Hz, 12H), 1.41 – 1.17 (m, 32H), 0.98 – 0.76 (m, 24H).

^{13}C NMR (101 MHz, $CDCl_3$) δ 161.3, 144.4, 142.8, 141.4, 139.6, 139.1, 133.3, 127.9, 127.0, 125.6, 125.5, 124.7, 123.9, 112.7, 60.3, 53.6, 53.1, 40.0, 30.7, 28.6, 24.0, 23.2, 14.8, 14.1, 10.8.

HRMS (MALDI, m/z) 1575.6796 [M+H$^+$] requires 1575.6776 for [$C_{92}H_{103}N_8O_8S_4^+$].

3F

Under a blanket of argon, **6** (200 mg, 0.300 mmol) and **16** (70.0 mg, 0.090 mmol) were dissolved in anhydrous pyridine (30 mL). The mixture was degassed by sparging with argon for 1 hour, then heated to reflux with stirring overnight. The red mixture was allowed to cool to room temperature and then poured directly into a beaker of stirring MeOH (250 mL) generating a bright red-orange precipitate. The precipitate was then collected by filtration and washed with MeOH the dried under a stream of air. The purification of the material was achieved by

column chromatography (SiO_2; $CHCl_3$) followed by precipitation from $CHCl_3$ with MeOH yielding **3F** (149 mg, 73%) as a red solid. M.p. >300°C.

^{1}H NMR (500 MHz, $CDCl_3$) δ 8.68 (s, 6H), 8.21 (s, 6H), 6.46 (s, 2H), 4.50 – 4.46 (m, 24H), 2.18 – 2.10 (m, 6H), 1.53 (t, *J* = 7.1 Hz, 18H), 1.42 – 1.26 (m, 48H), 0.93 – 0.84 (m, 36H).

^{13}C NMR (126 MHz, $CDCl_3$) δ 161.1, 143.3, 141.6, 139.7, 139.3, 133.6, 128.0, 125.6, 125.6, 124.7, 112.8, 60.4, 53.1, 40.0, 30.7, 28.6, 23.9, 23.2, 14.8, 14.7 14.1, 10.8.

HRMS (MALDI, m/z) 2235.9727 [$M+H^+$] requires 2235.9580 for [$C_{128}H_{147}N_{12}O_{12}S_6^+$].

NMR Spectra

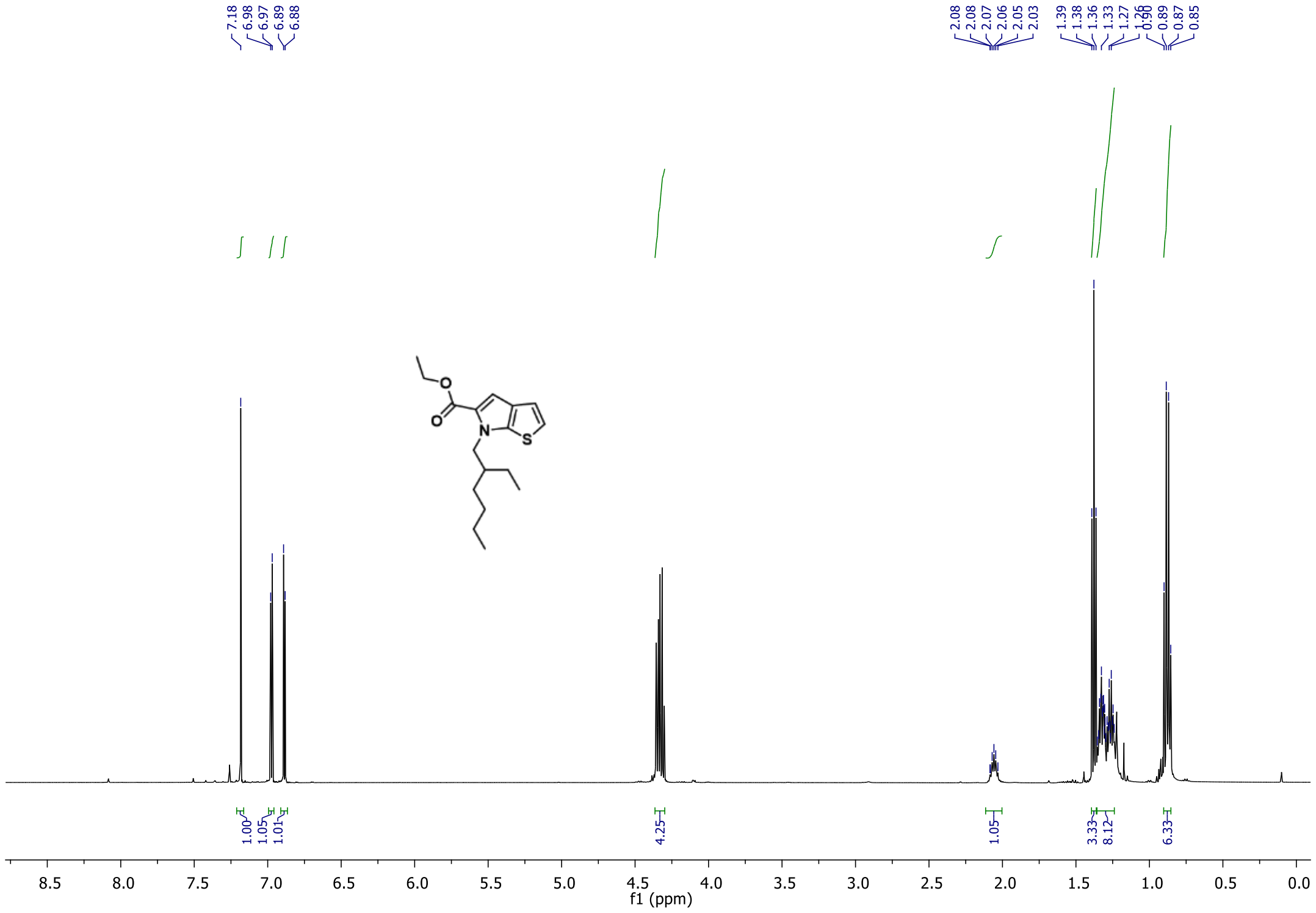

Spectrum S1. Compound **3** ^{1}H NMR spectrum.

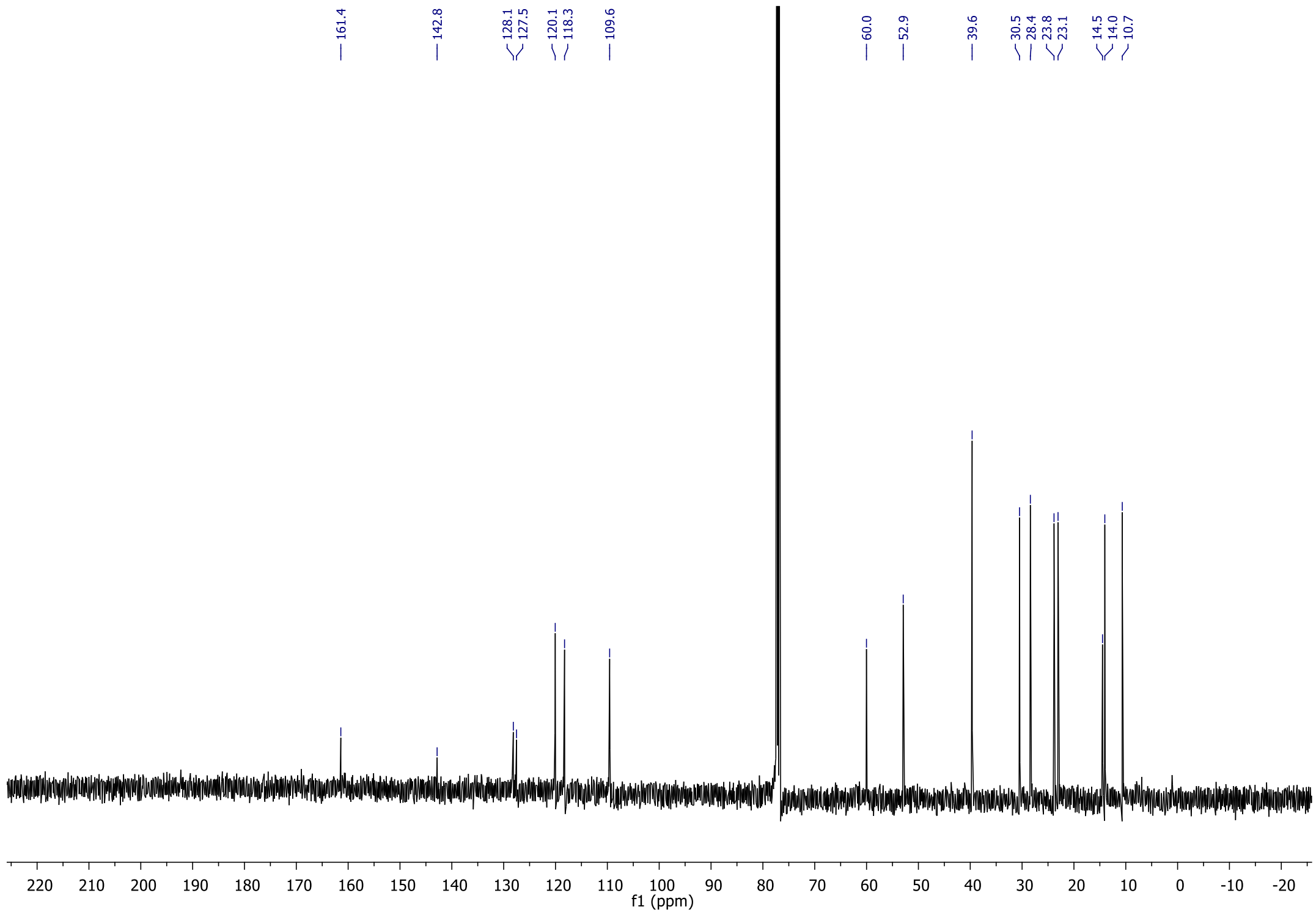

Spectrum S2. Compound **3** ^{13}C NMR spectrum.

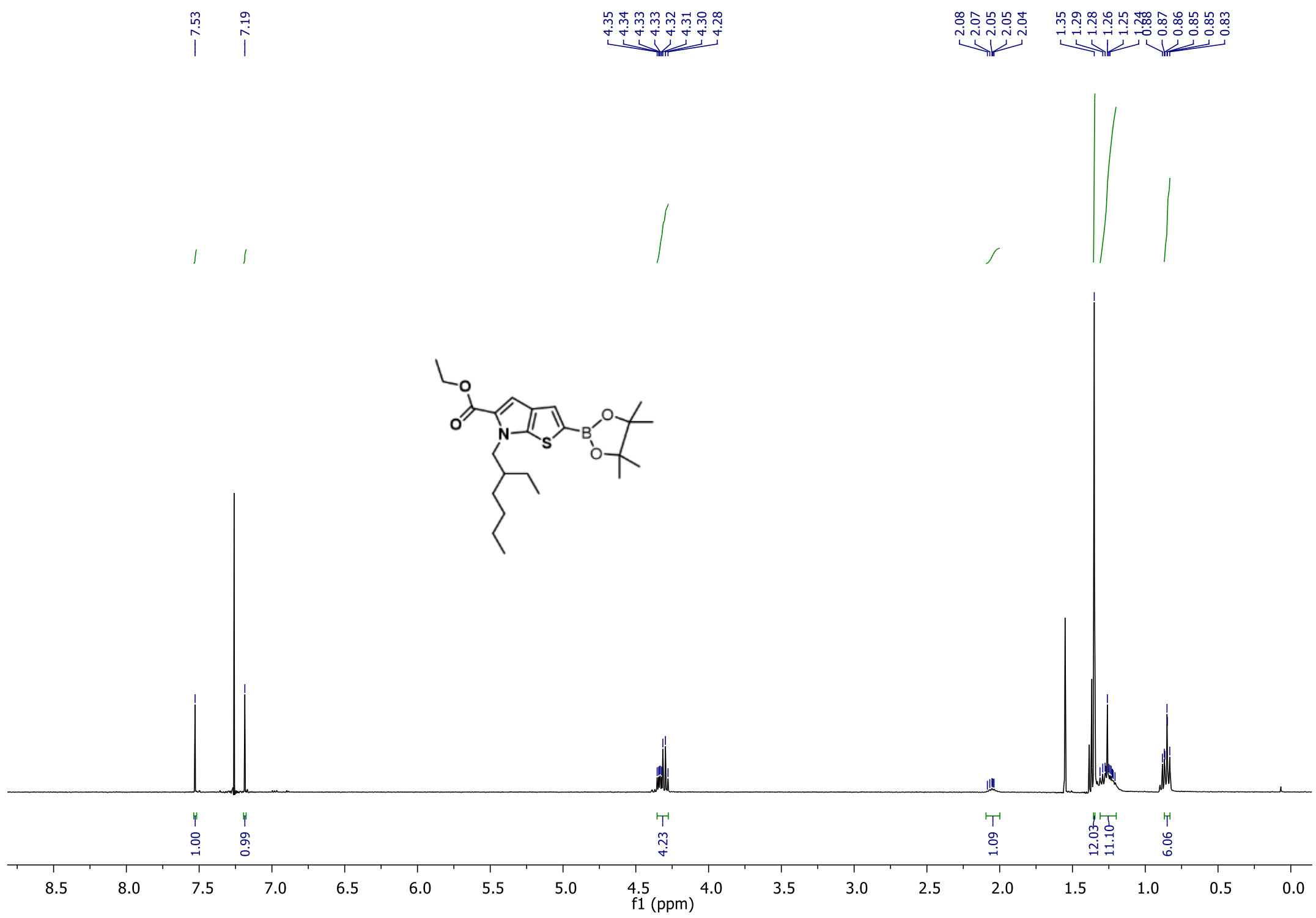

Spectrum S3. Compound **4** ^{1}H NMR spectrum.

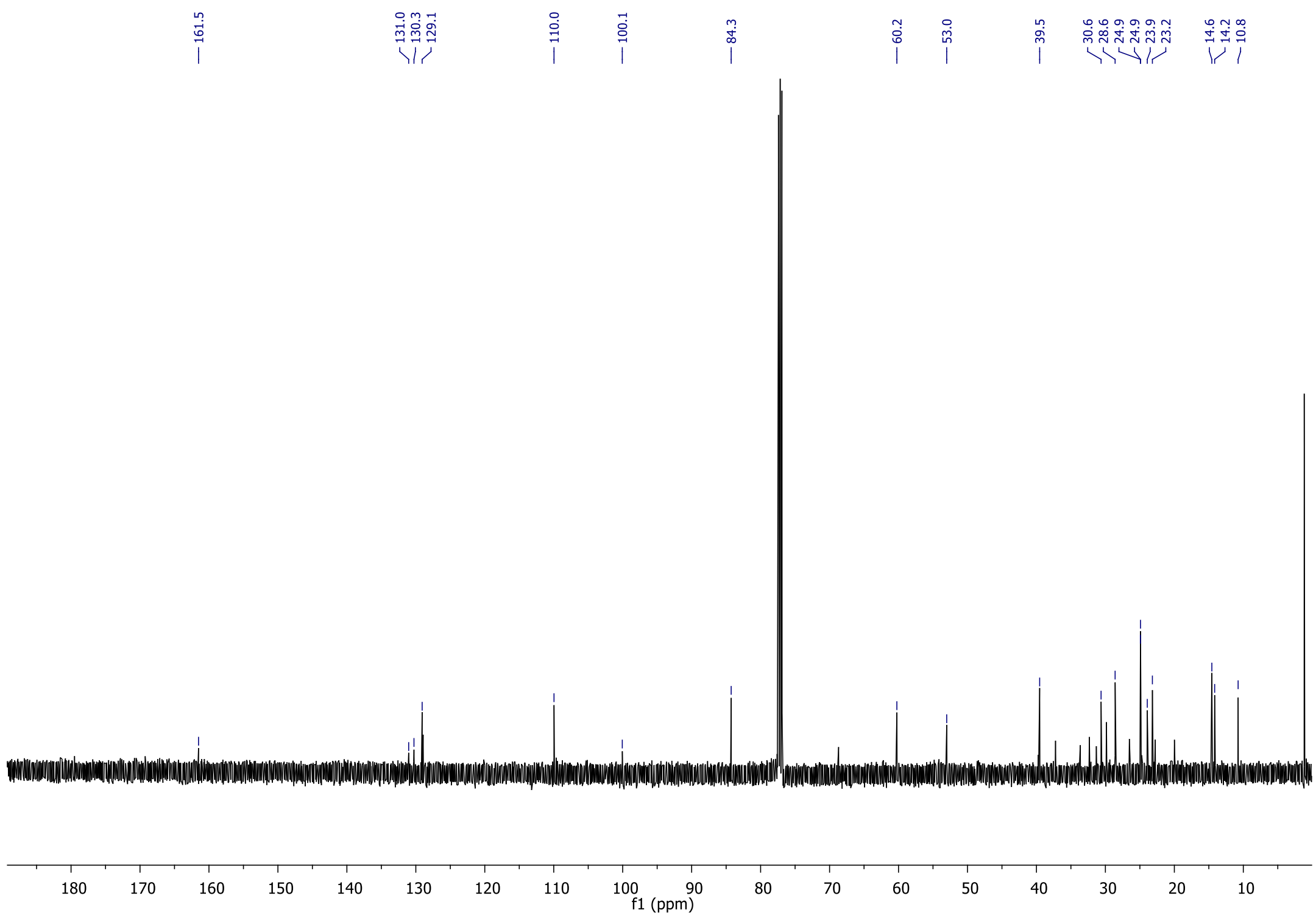

Spectrum S4. Compound **4** ^{13}C NMR spectrum.

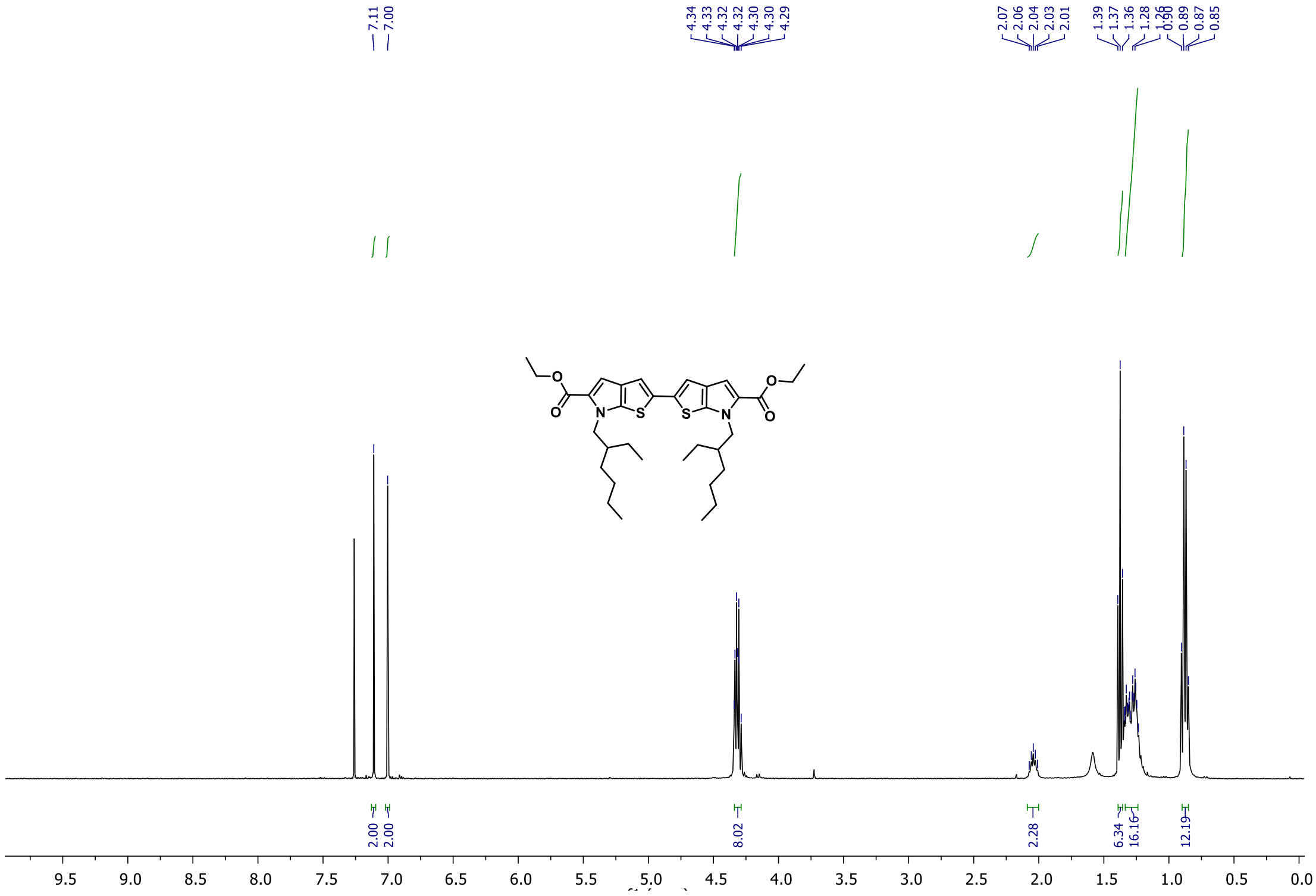

Spectrum S5. Compound **5** ^{1}H NMR spectrum.

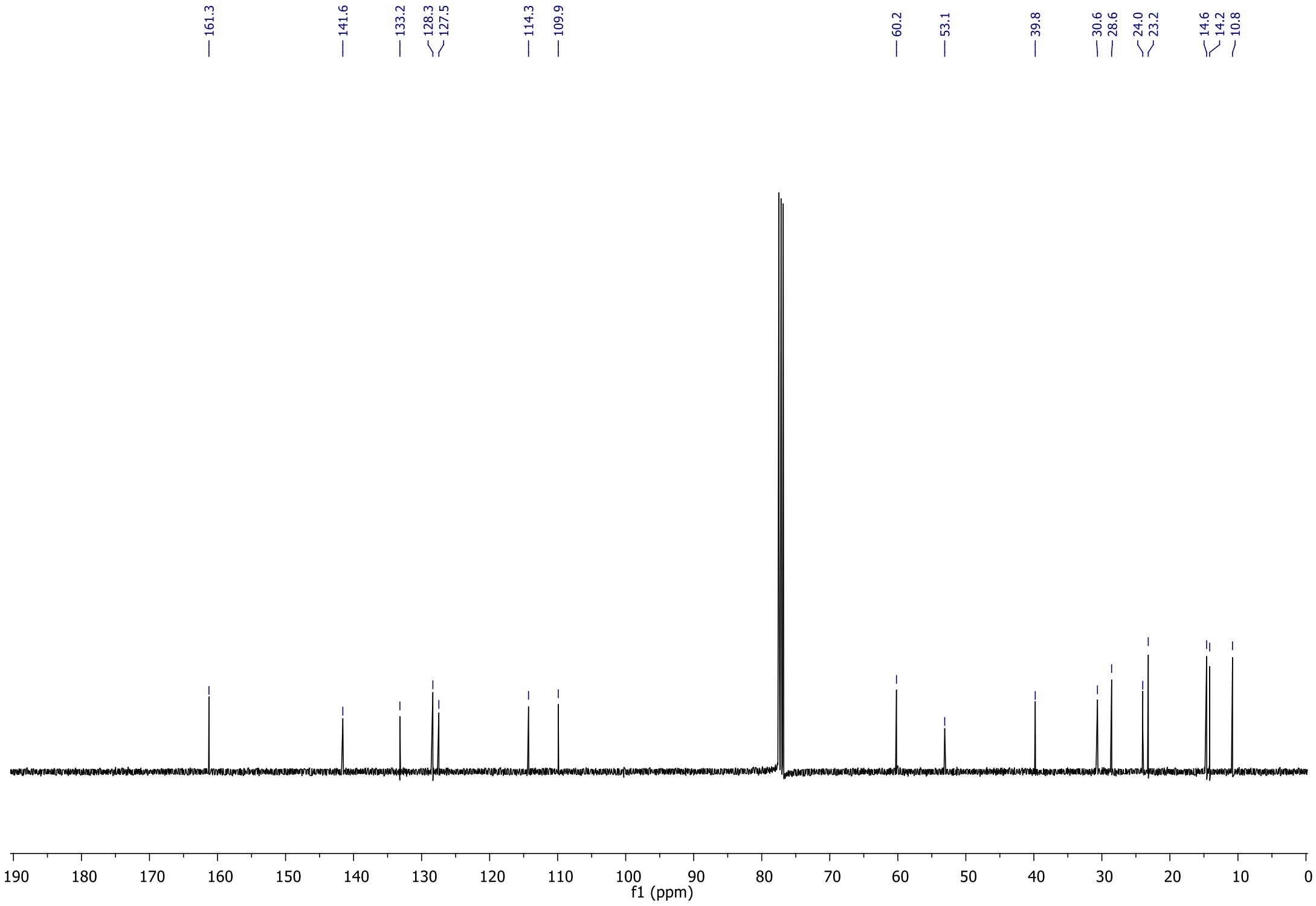

Spectrum S6. Compound **5** ^{13}C NMR spectrum.

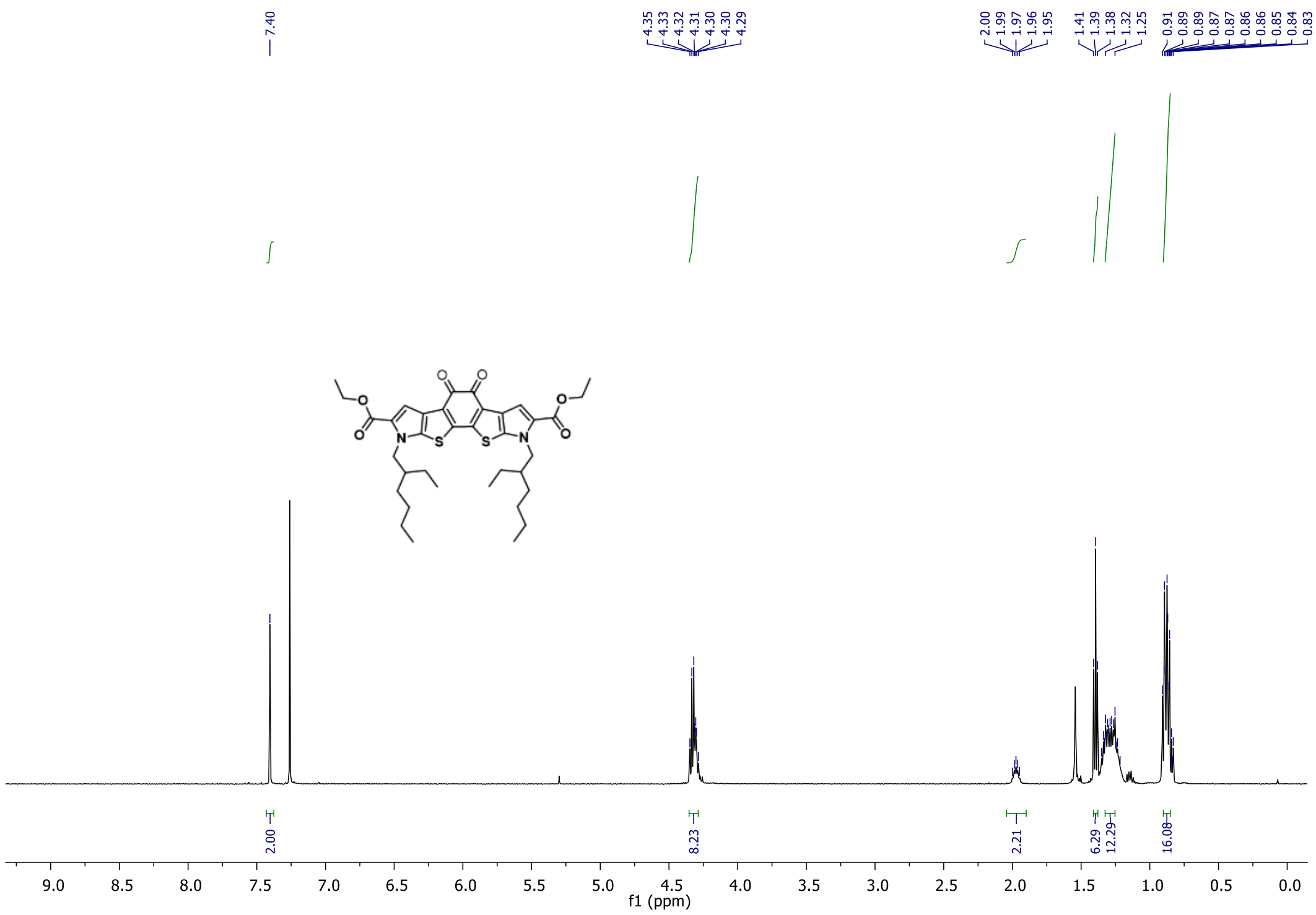

Spectrum S7. Compound **6** ^{1}H NMR spectrum.

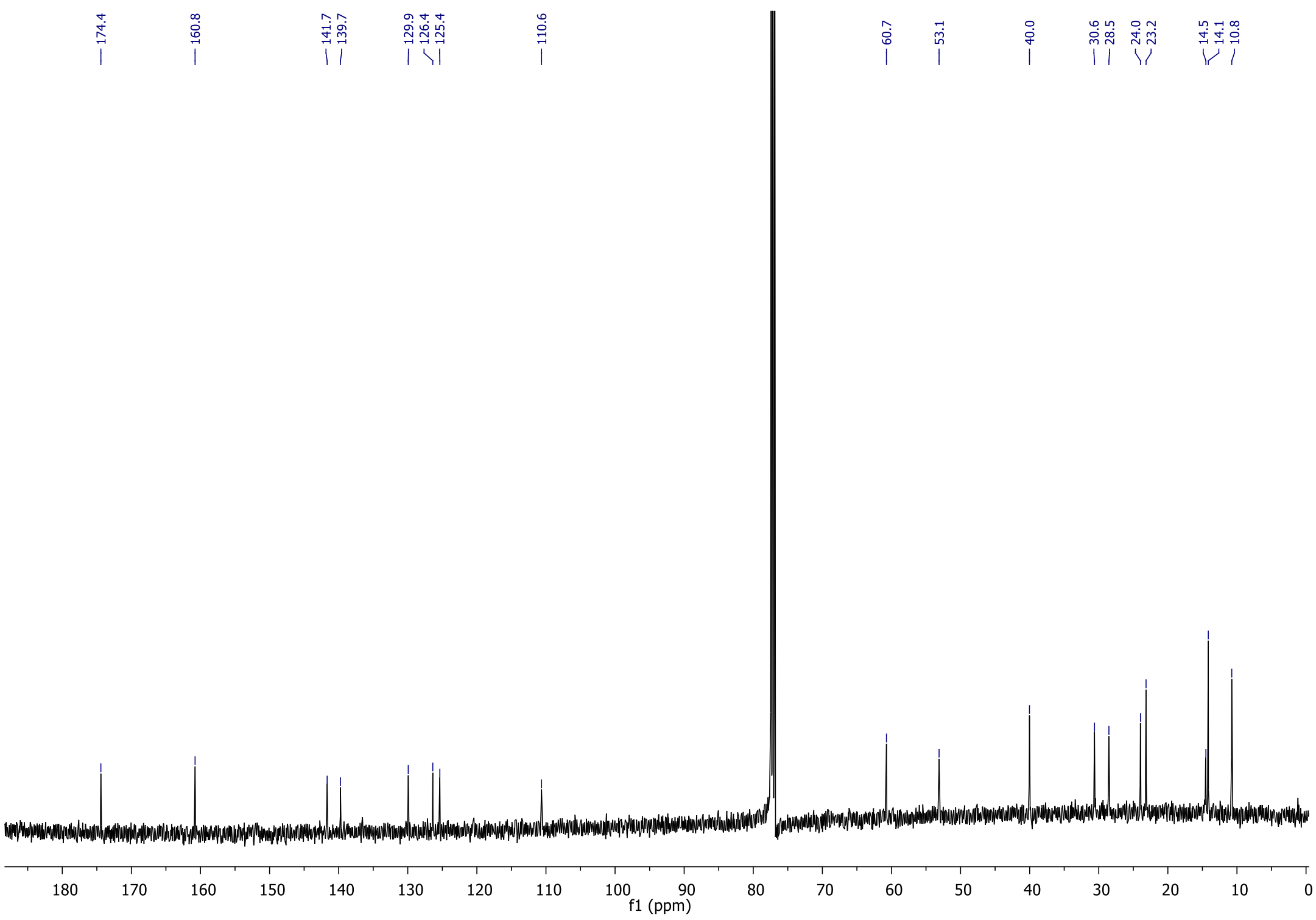

Spectrum S8. Compound **6** ^{13}C NMR spectrum.

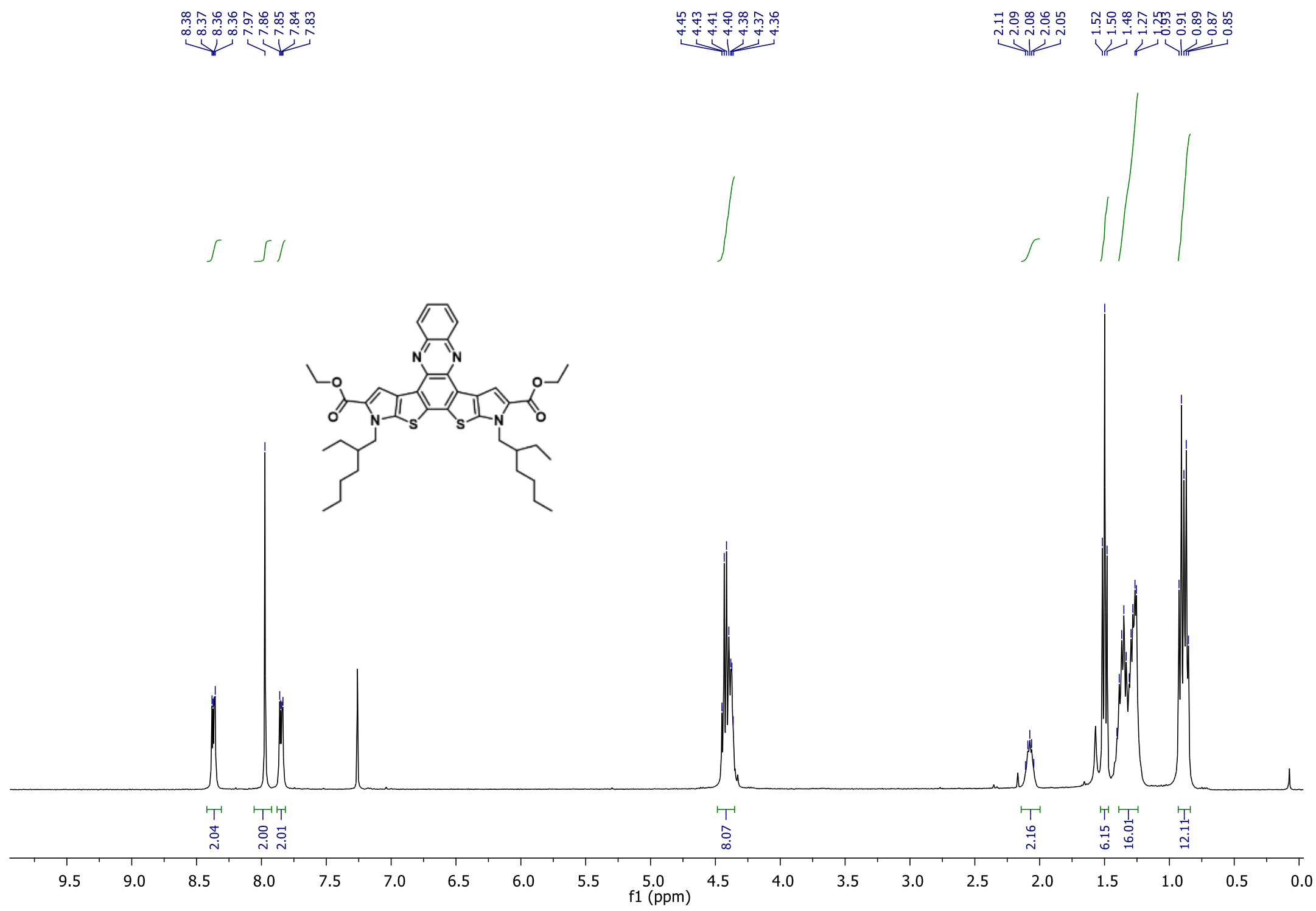

Spectrum S9. Compound **0F** ^{1}H NMR spectrum.

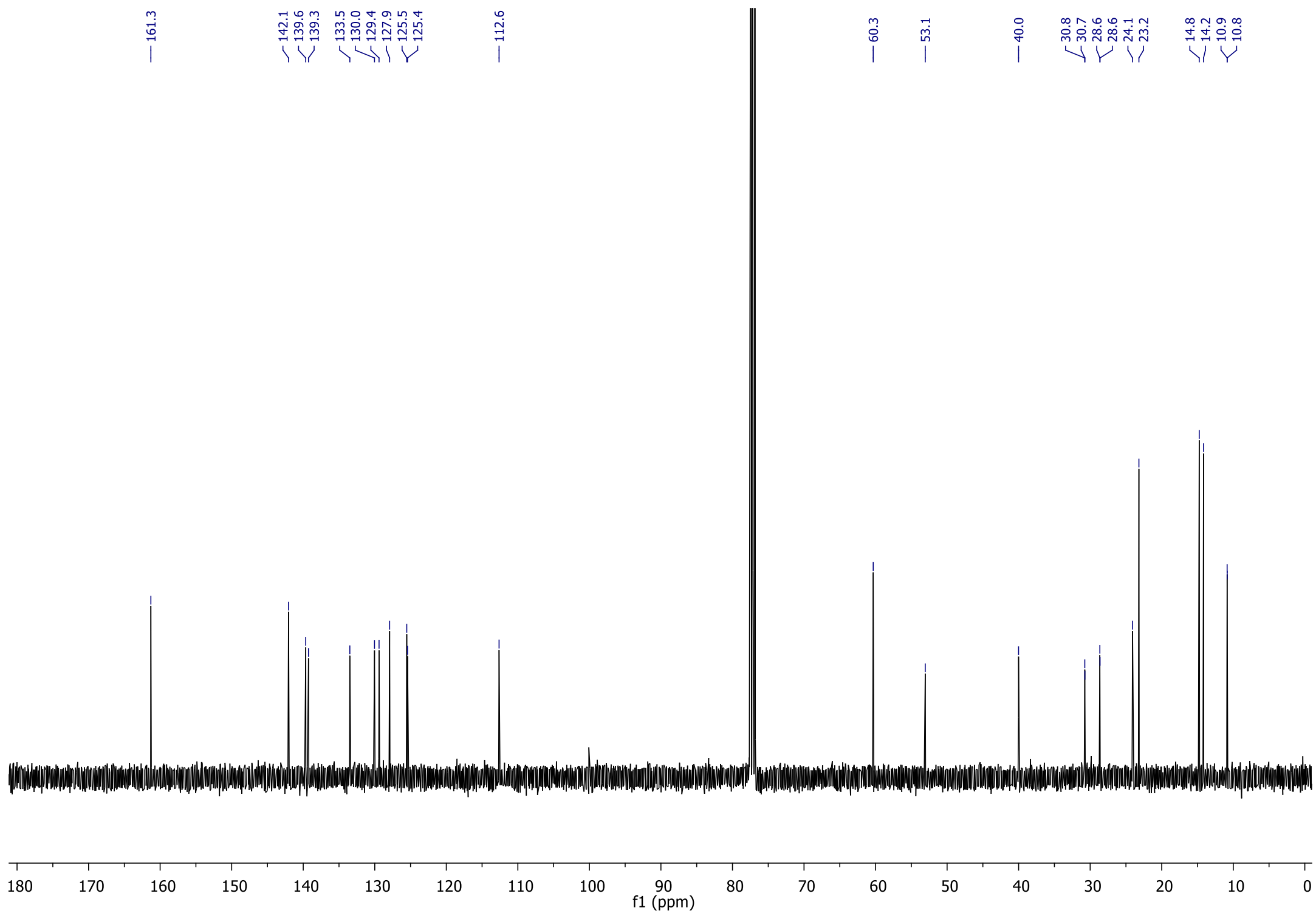

Spectrum S10. Compound **0F** ^{13}C NMR spectrum.

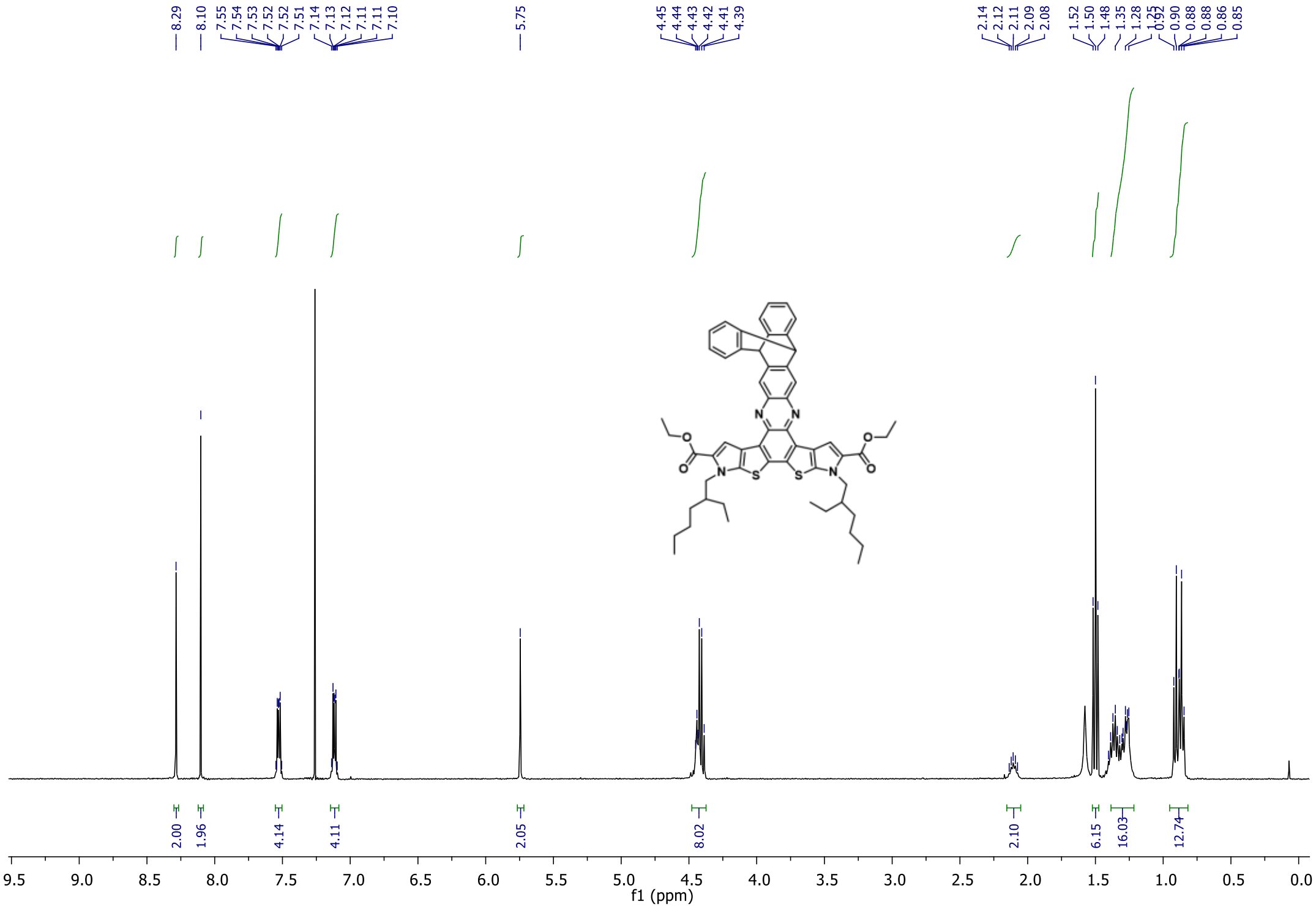

Spectrum S11. Compound **1F** ^{1}H NMR spectrum.

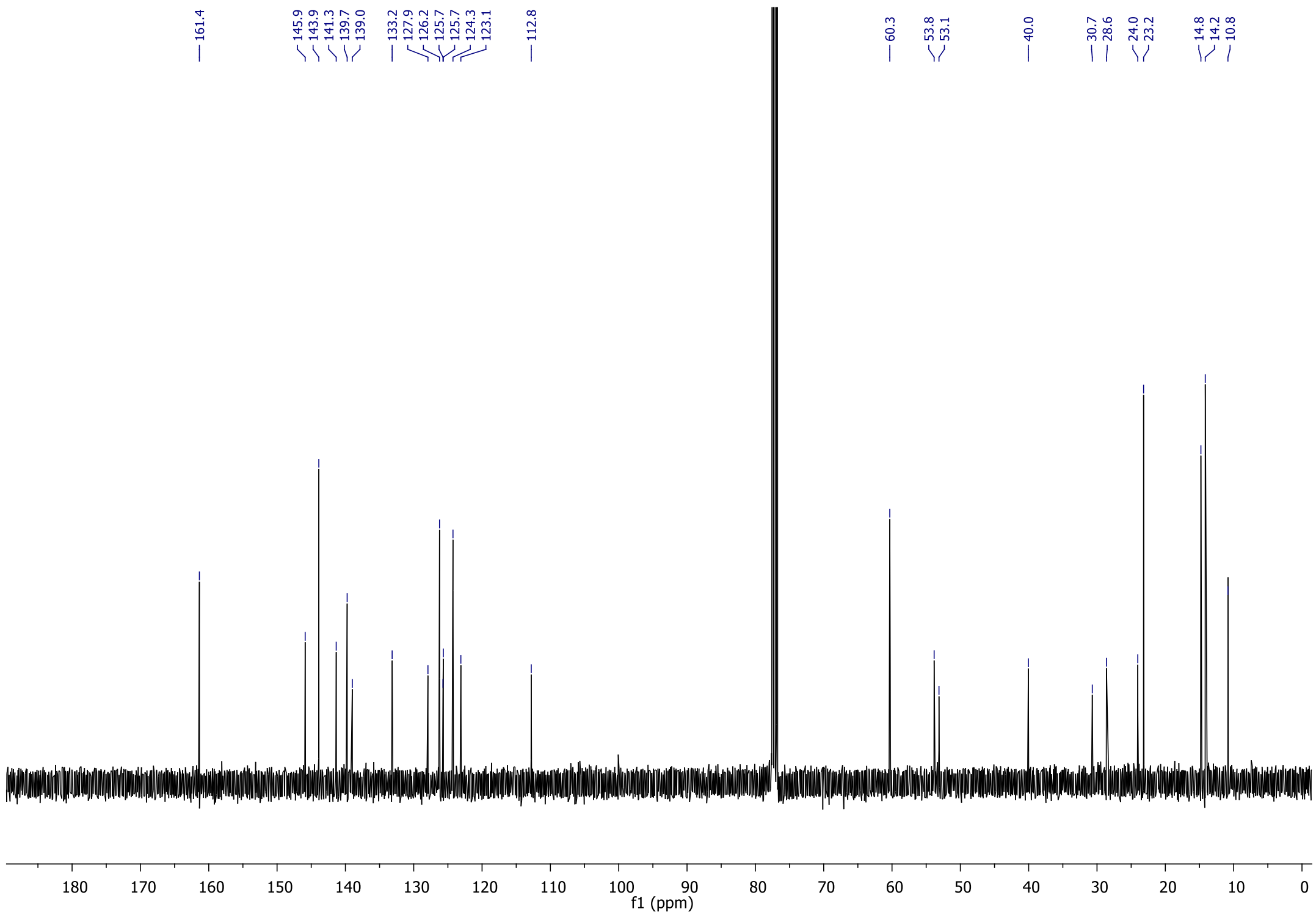

Spectrum S12. Compound **1F** ^{13}C NMR spectrum.

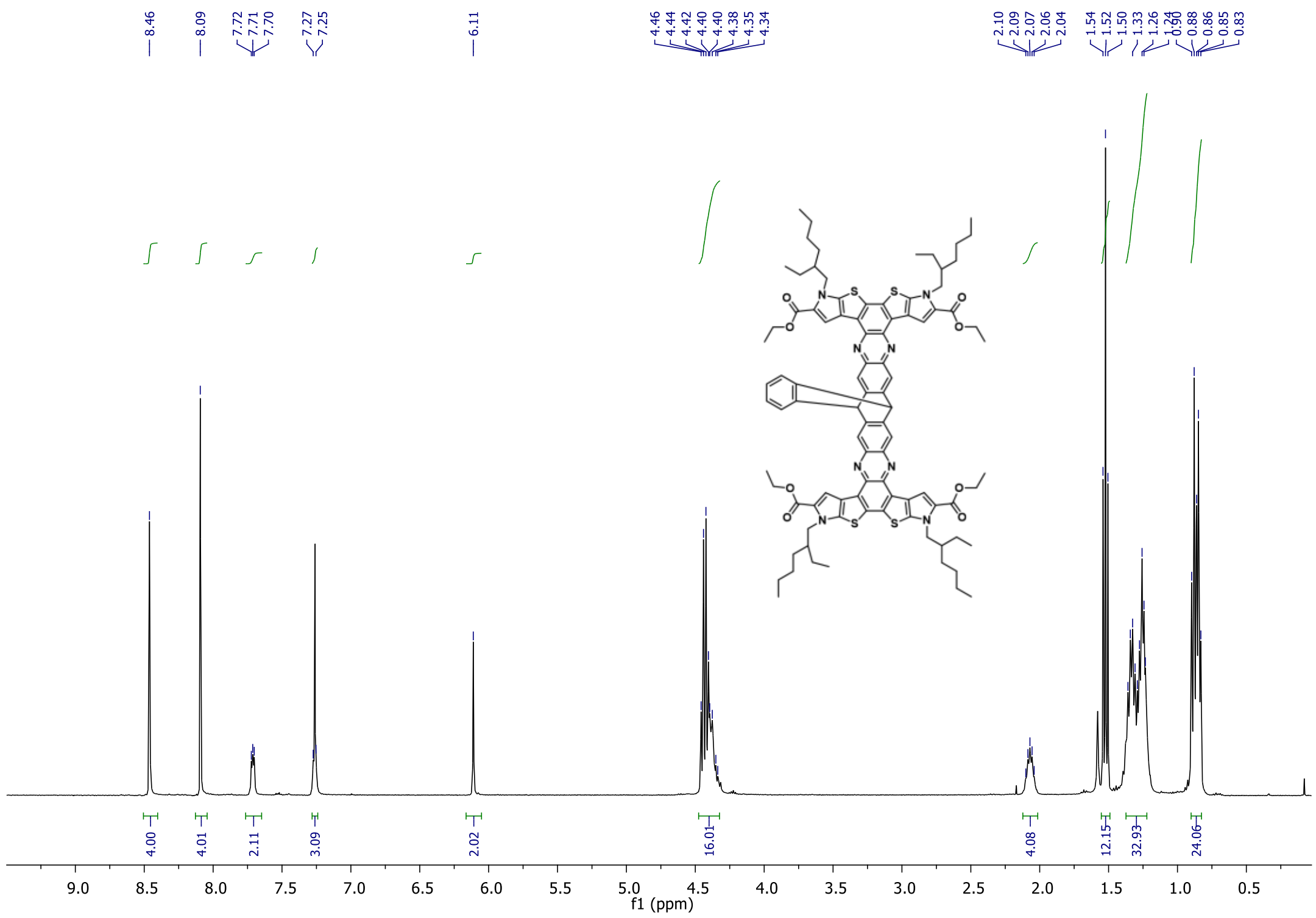

Spectrum S13. Compound **2F** ^{1}H NMR spectrum.

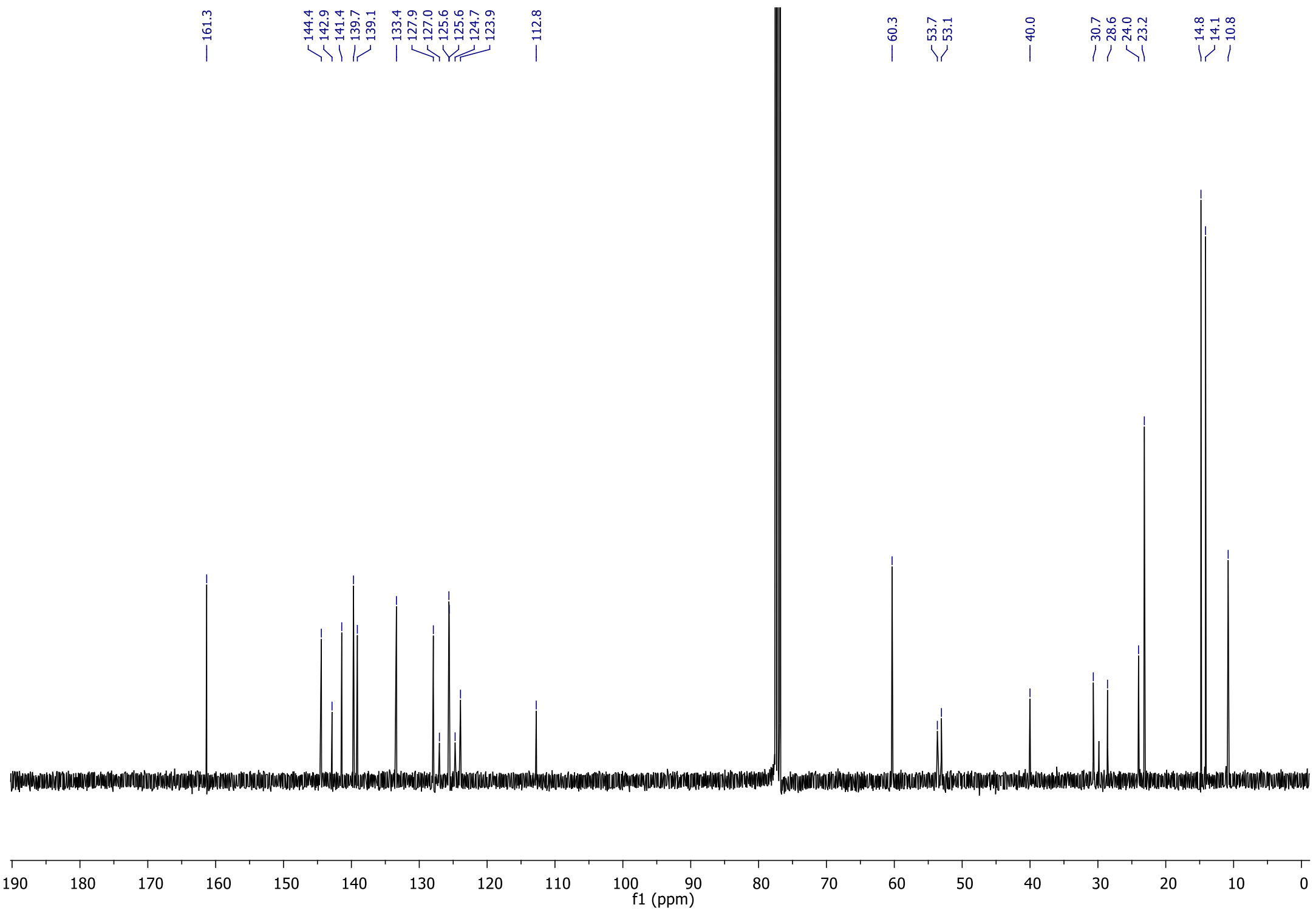

Spectrum S14. Compound **2F** ^{13}C NMR spectrum in $CDCl_3$.

llll

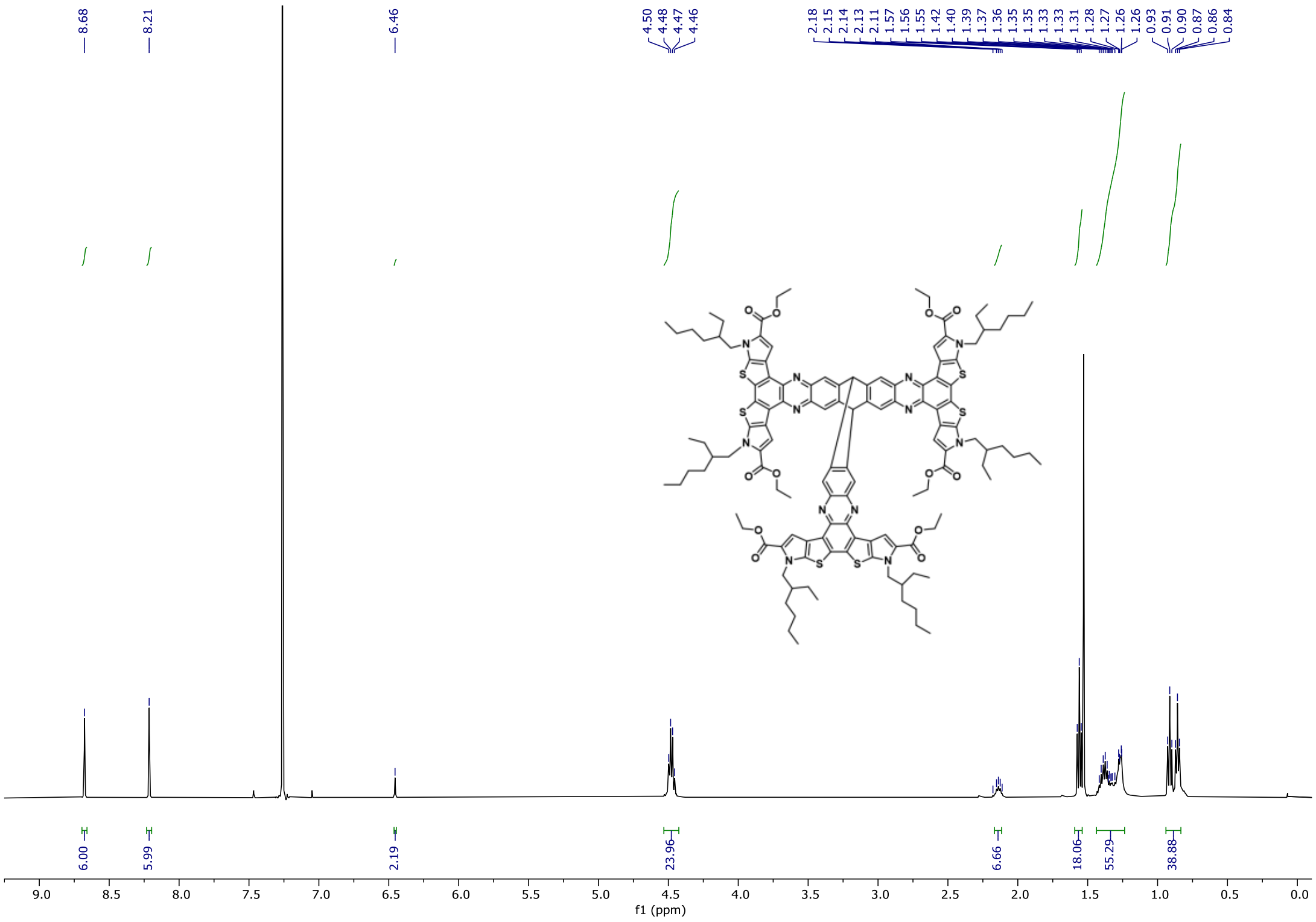

Spectrum S15. Compound **3F** ^{1}H NMR spectrum.

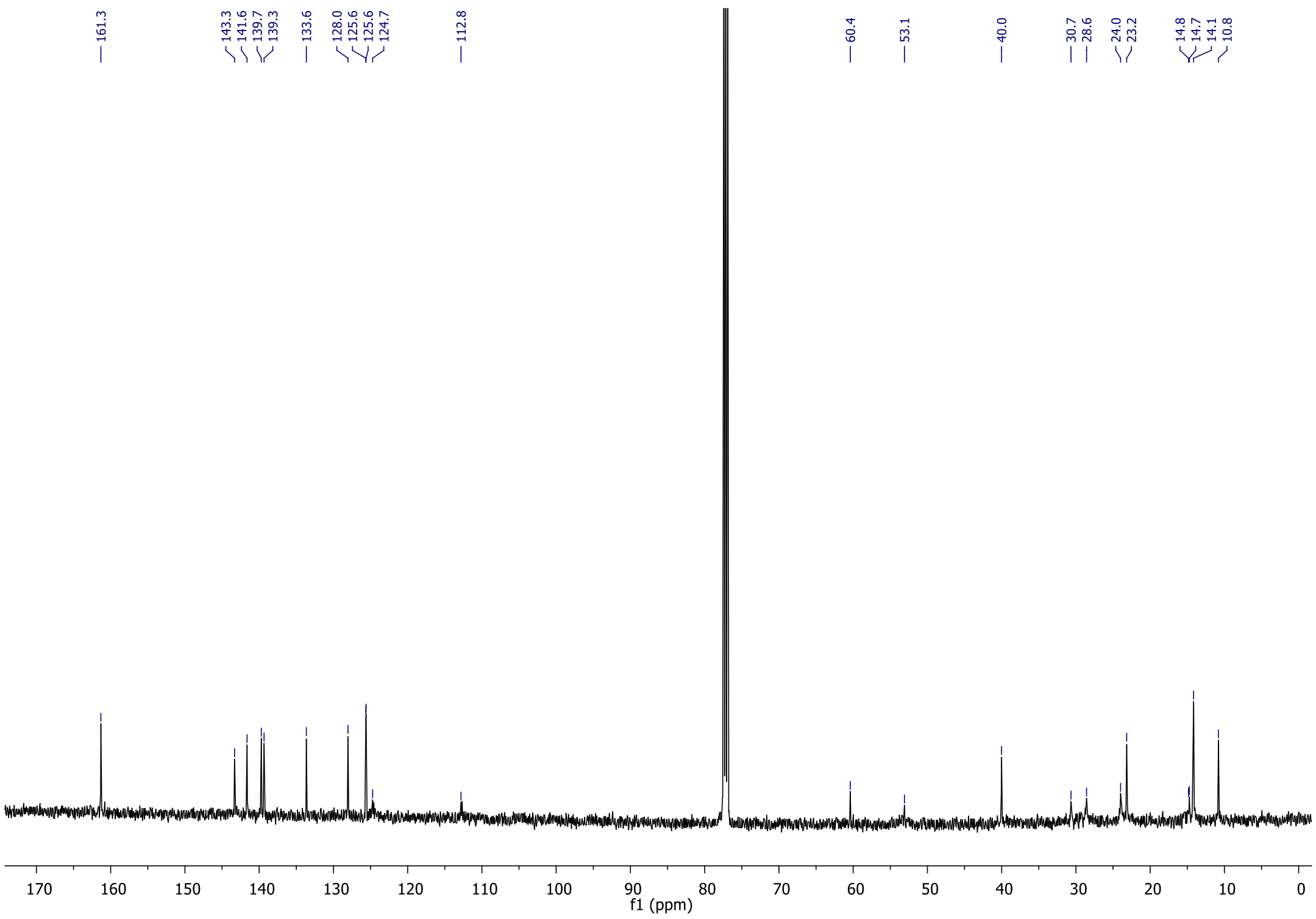

Spectrum S16. Compound **3F** ^{13}C NMR spectrum.

Mass Spectrometry

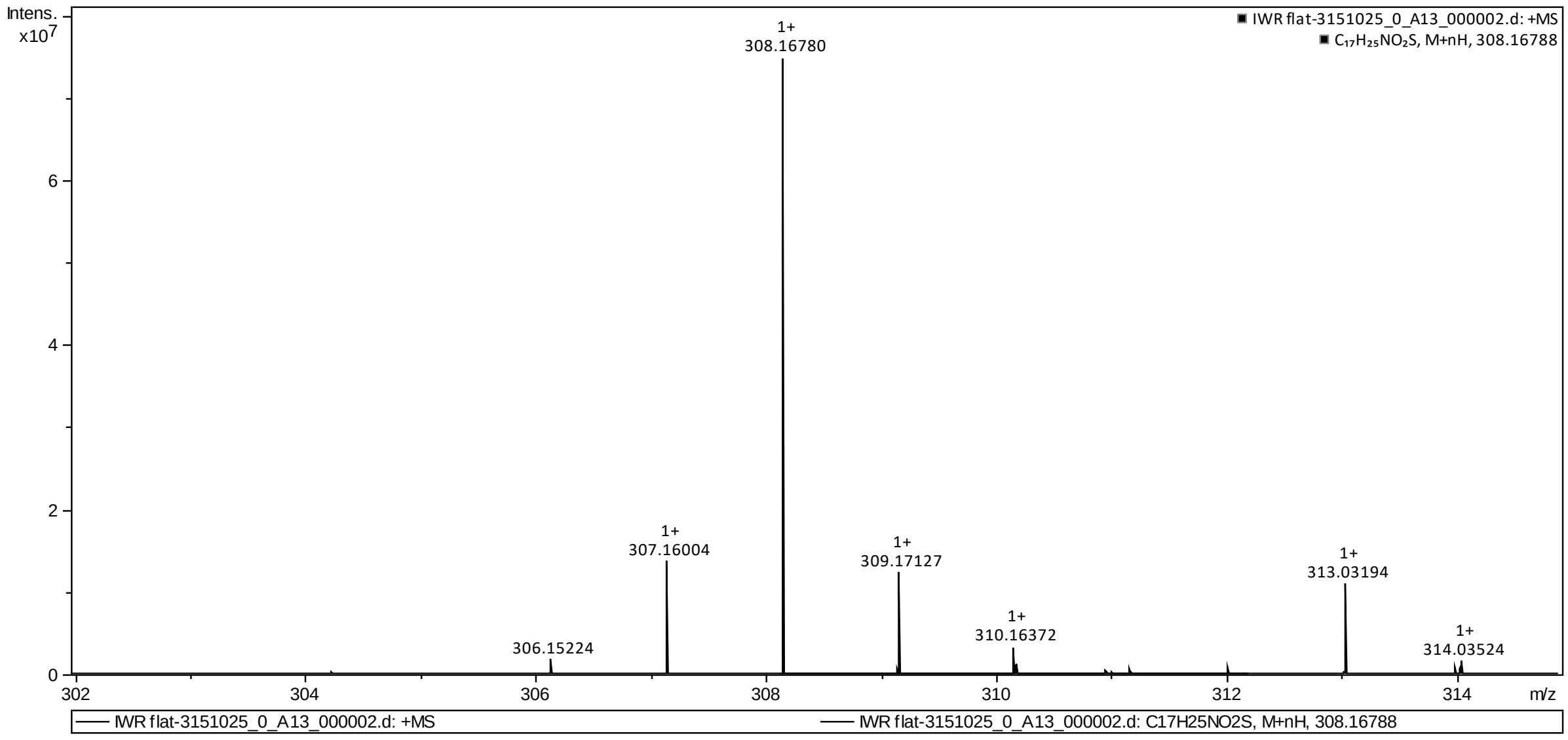

Spectrum S17. Compound **3** HRMS spectrum.

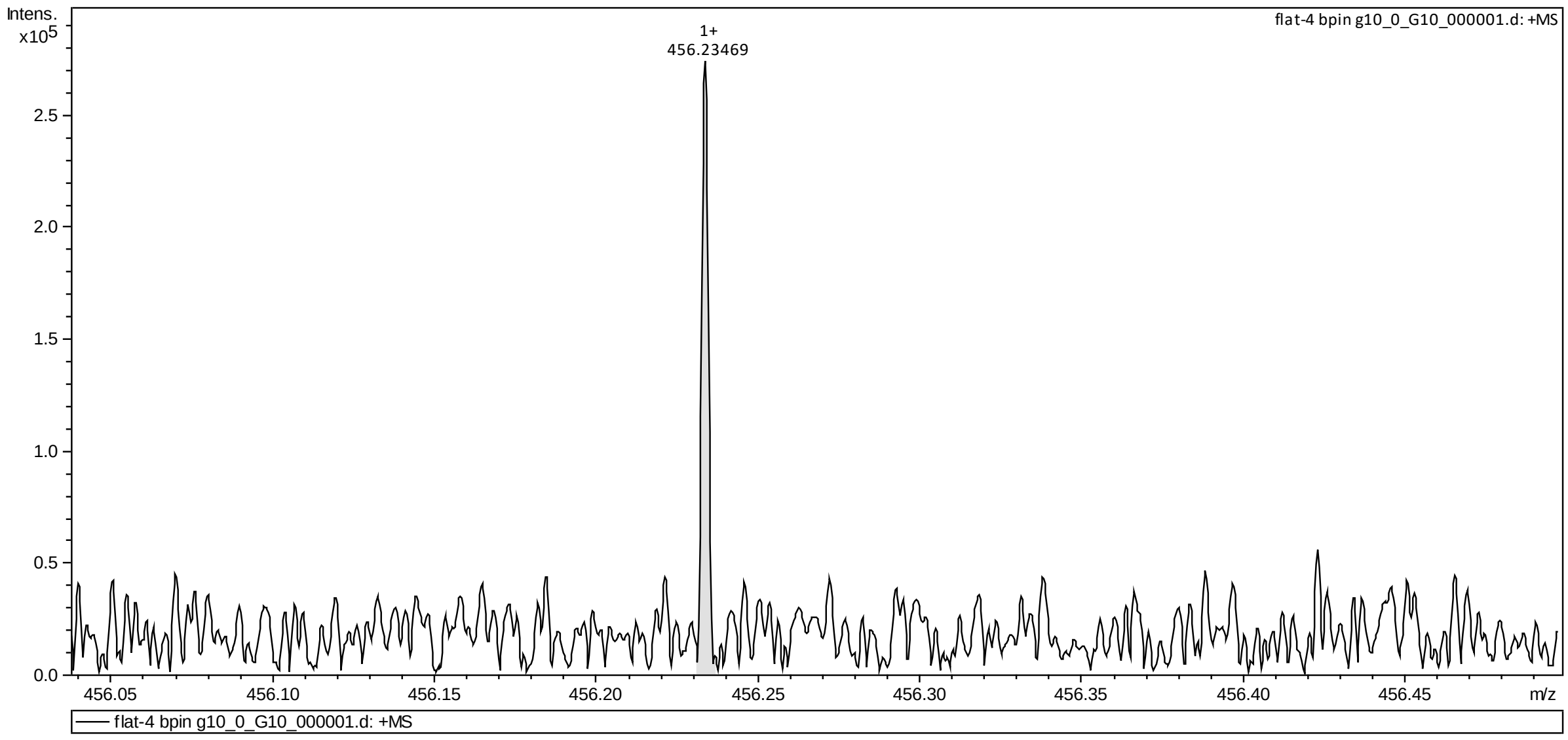

Spectrum S18. Compound **4** HRMS spectrum.

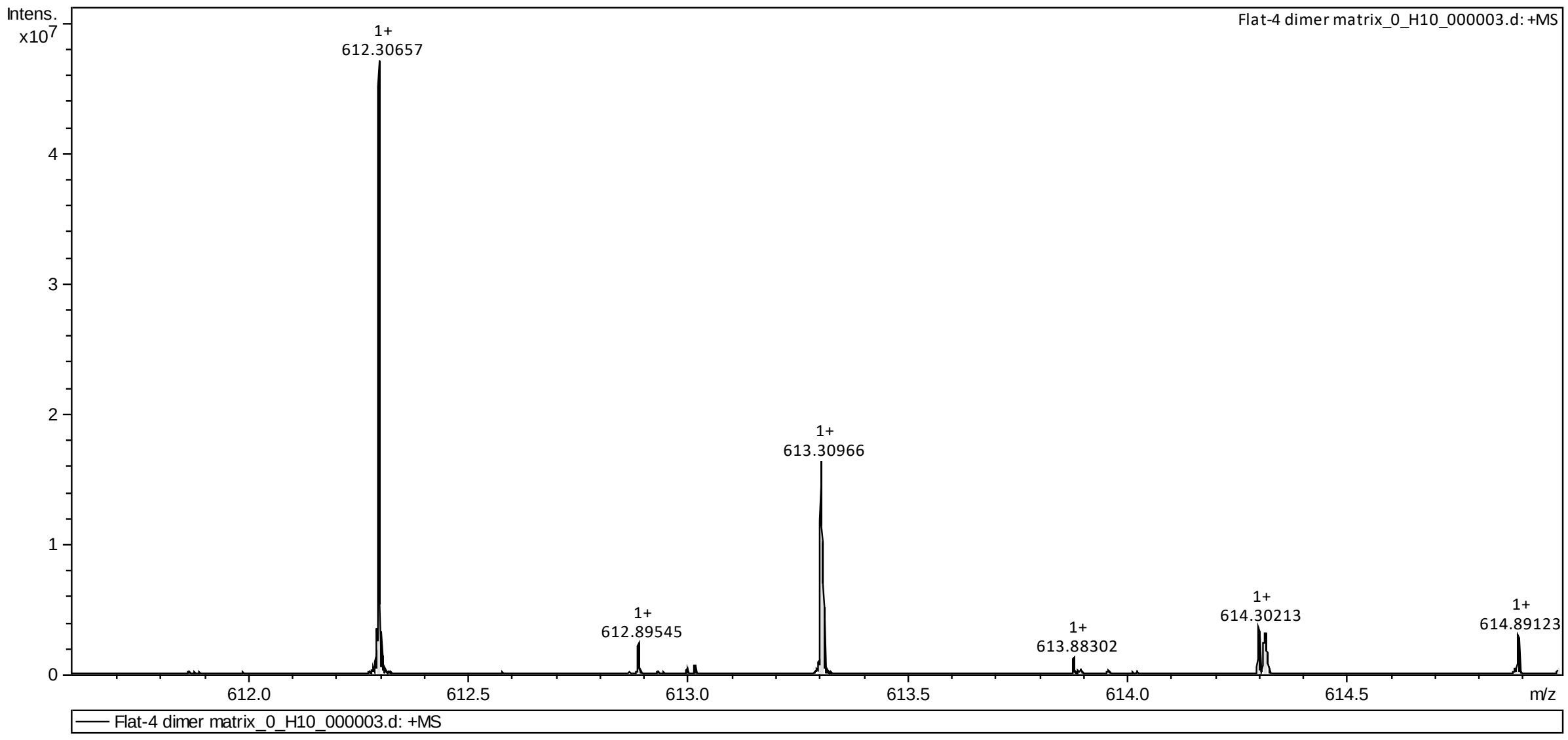

Spectrum S19. Compound **5** HRMS spectrum.

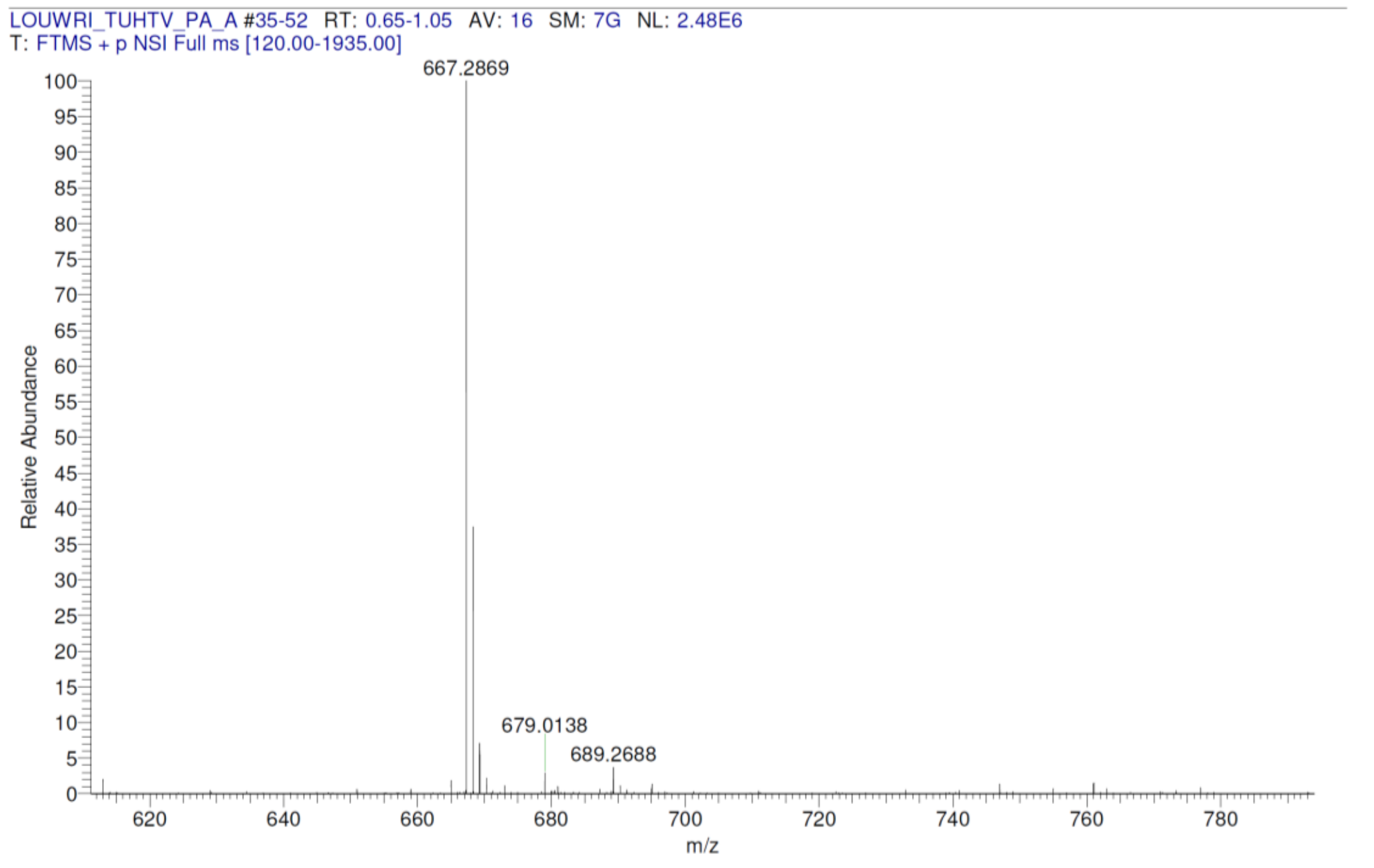

Spectrum S20. Compound **6** HRMS spectrum.

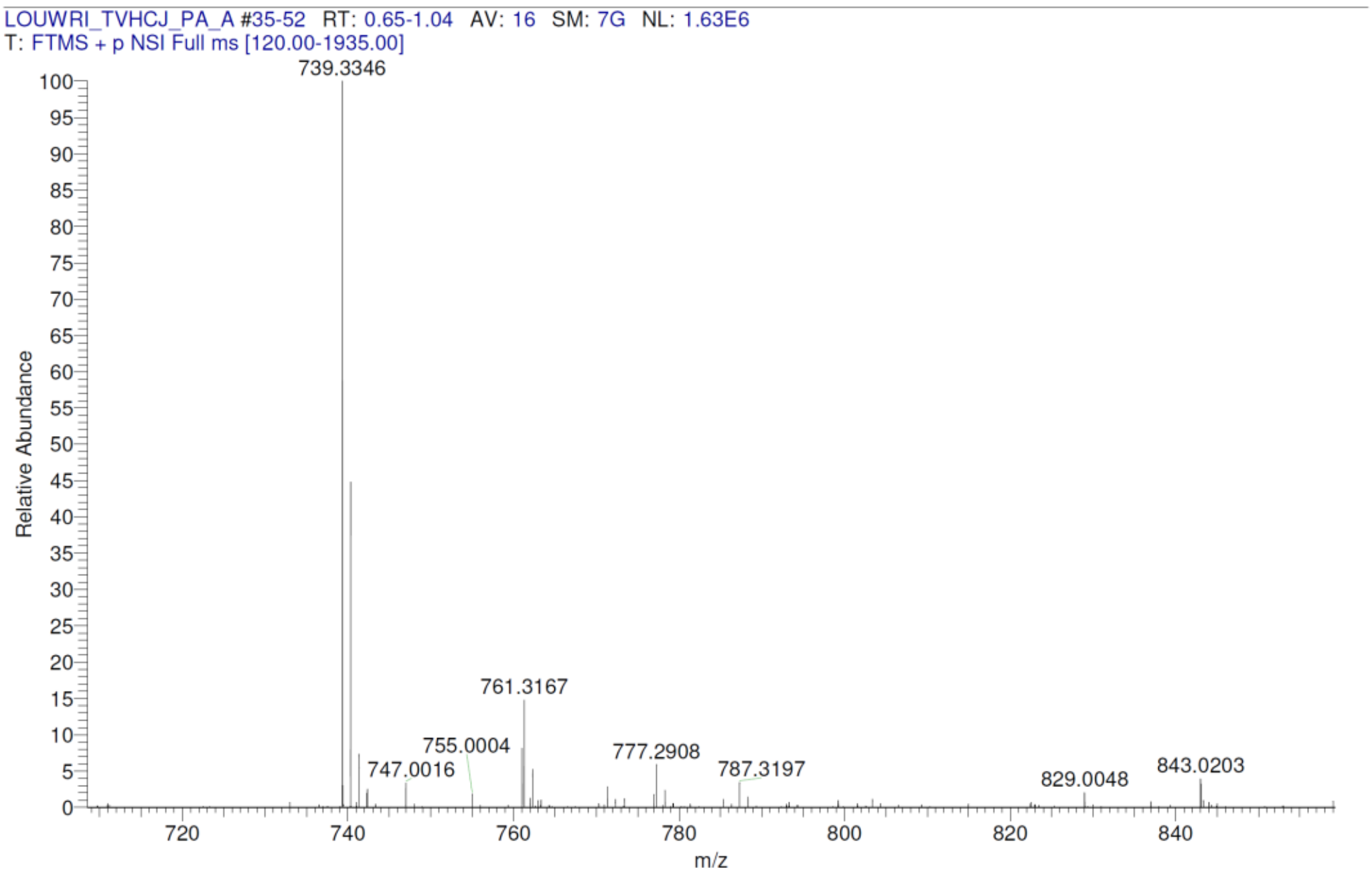

Spectrum S21. Compound **0F** HRMS spectrum.

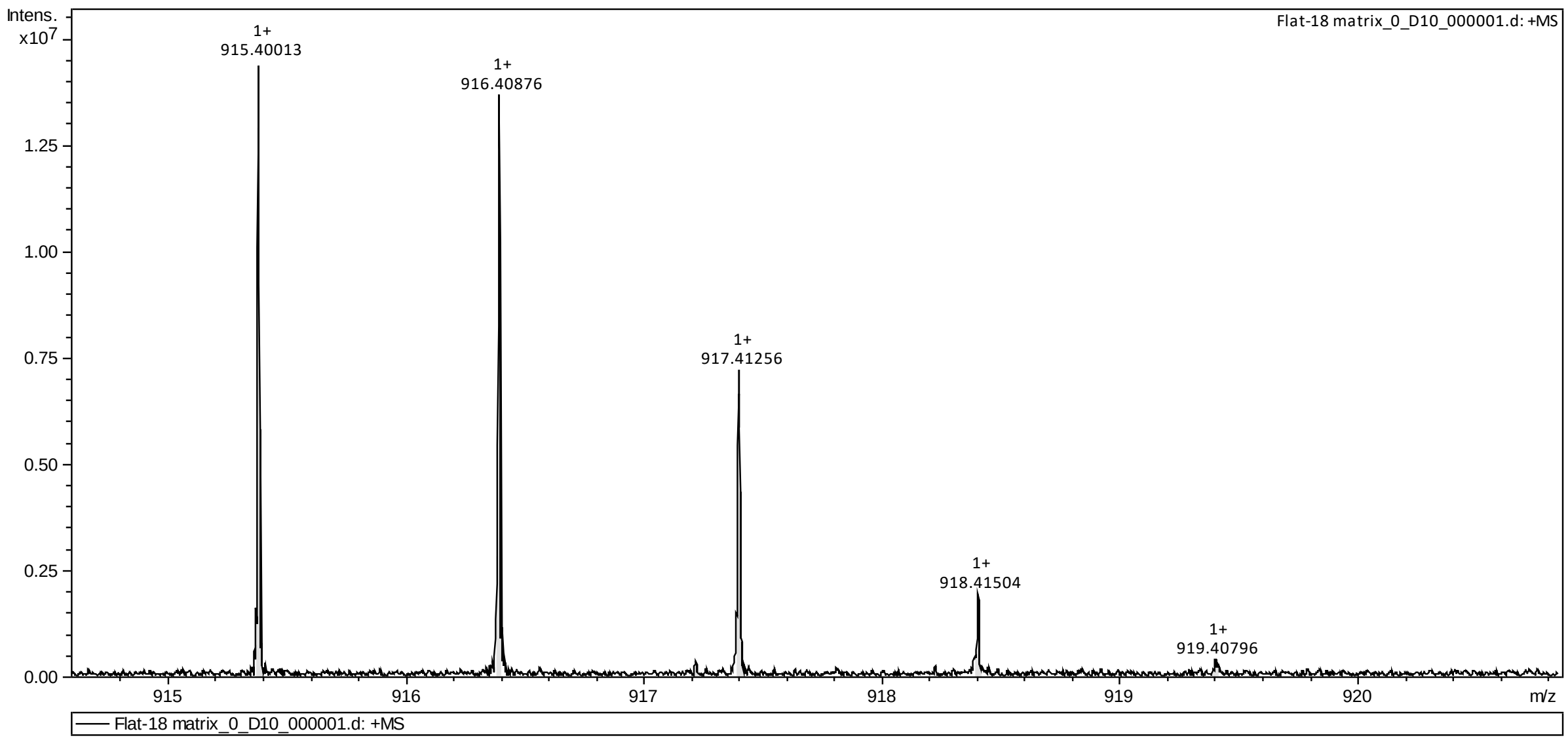

Spectrum S22. Compound **1F** HRMS spectrum.

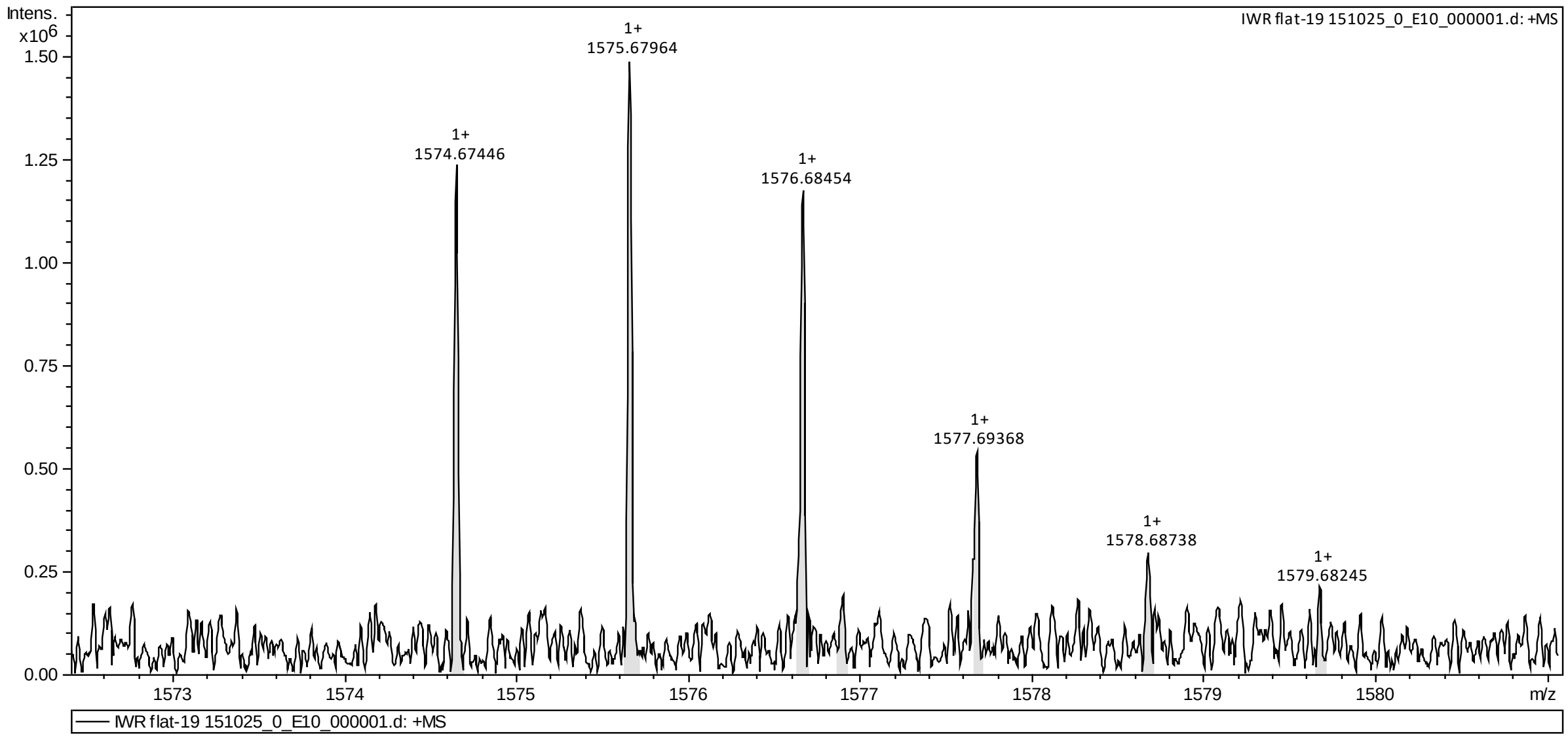

Spectrum S23. Compound **2F** HRMS spectrum.

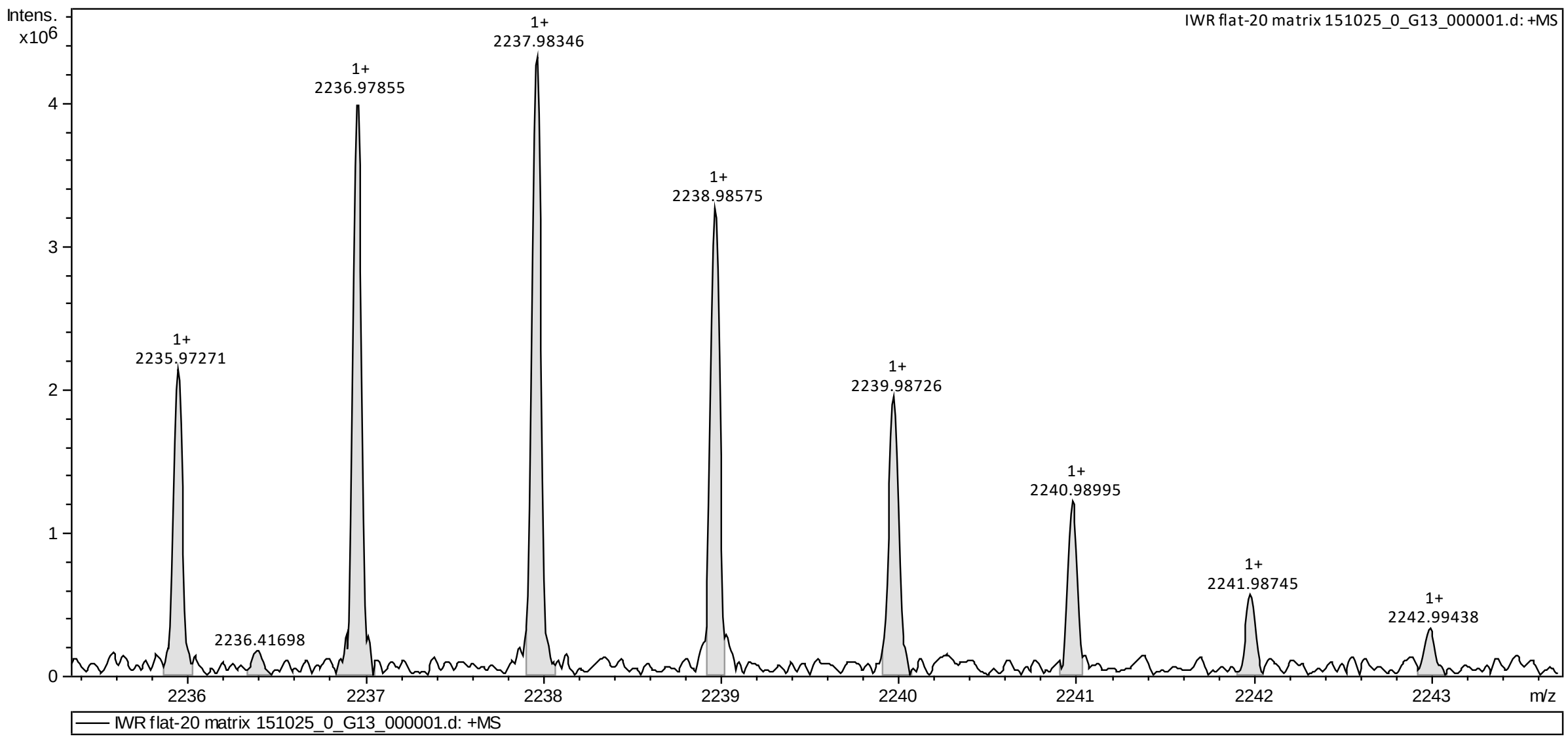

Spectrum S24. Compound **3F** HRMS spectrum.

X-Ray Crystallography

For **0F**, X-ray diffraction data were collected using a Rigaku 007HF diffractometer with HF Varimax confocal mirrors, an UG2 goniometer and HyPix 6000HE detector equipped with an Oxford Cryosystems low-temperature device, operating at T = 100(2) K. For **1F**, Data were collected using a Rigaku Oxford Diffraction SuperNova diffractometer equipped with an Oxford Cryosystems Cryostream 700+ low-temperature device operating at T = 100.0 K.

Table S1: Crystal data and experimental details.

**Compound**	**0F**	**1F**
CCDC dep.no	**2495341**	**2502203**
Formula	$C_{42}H_{49}N_4O_4S_2$	$C_{56}H_{57}N_4O_4S_2$
$D_{calc.}$/ g cm^{-3}	1.252	1.265
μ/mm^{-1}	1.600	1.410
Formula Weight	737.97	914.17
Colour	orange	dark red
Shape	block-shaped	prism-shaped
Size/mm^3	0.21×0.03×0.03	0.15×0.06×0.05
T/K	100(2)	100.0
Crystal System	triclinic	triclinic
Space Group	P-1	P-1
a/Å	7.17820(10)	12.6908(4)
b/Å	15.4628(2)	13.6471(6)
c/Å	18.9985(3)	14.8875(6)
α/°	110.3290(10)	98.276(4)
β/°	93.0260(10)	101.073(3)
γ/°	96.0140(10)	104.494(3)
V/Å^3	1957.49(5)	2399.33(17)
Z	2	2
Z'	1	1
Wavelength/Å	1.54184	1.54184
Radiation type	Cu K_{α}	Cu K_{α}
Θ_{min}/°	2.492	3.415
Θ_{max}/°	76.801	76.378
Measured Refl's.	64079	50470
Indep't Refl's	7681	9933
Refl's I≥2 σ(I)	6951	6801
R_{int}	0.0433	0.0732
Parameters	561	1179
Restraints	14	1693
Largest Peak	0.932	0.781
Deepest Hole	-0.804	-0.462
GooF	1.113	1.025
wR_2 (all data)	0.2080	0.2387
wR_2	0.2028	0.2009
R_1 (all data)	0.0785	0.1009
R_1	0.0720	0.0732

Computational

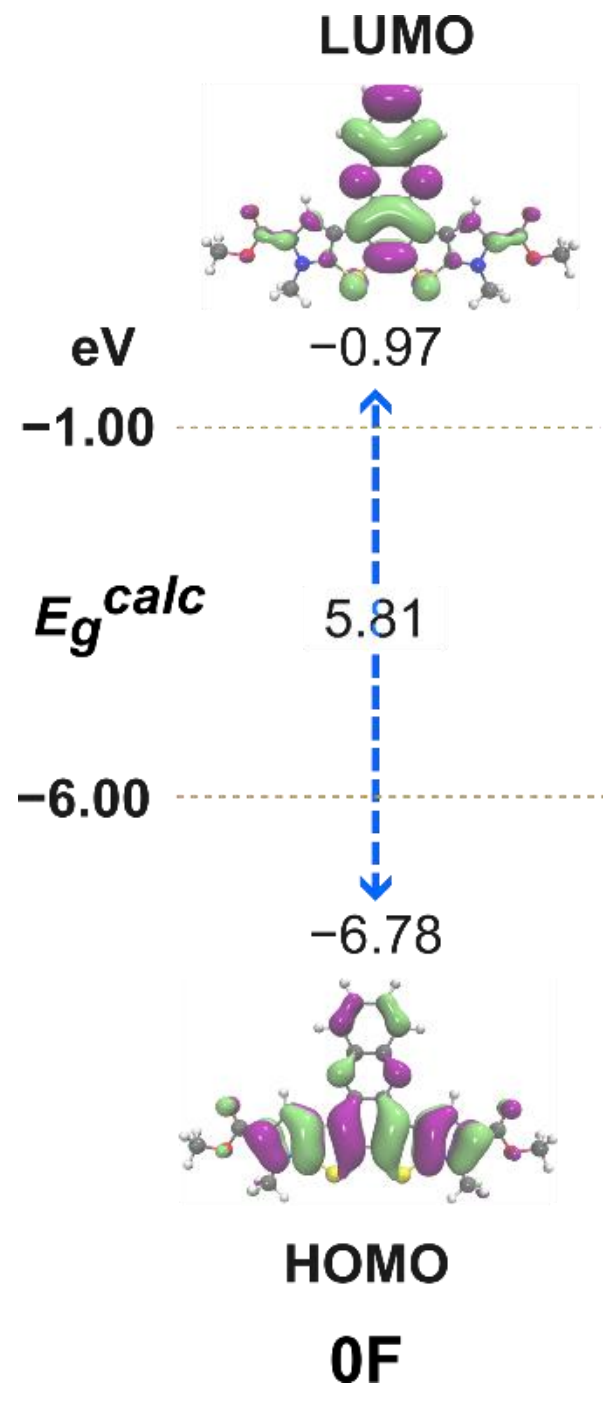

Figure S1 Calculated (B3LYP/def2-TZVP) frontier molecular orbital distributions and energies for **0F**.

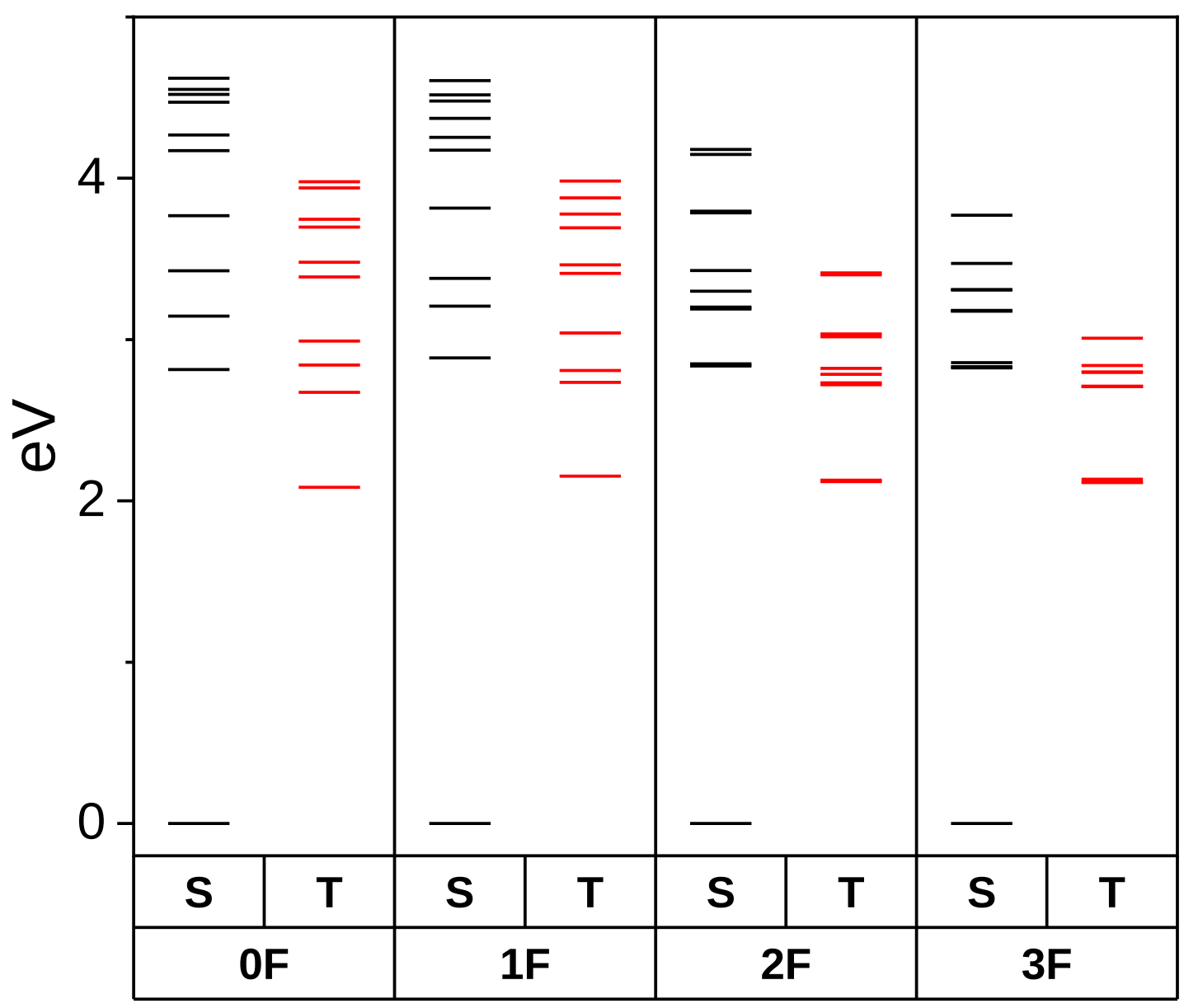

Figure S2 Energy profile of the 10 lowest-lying singlet and triplet excited states.

Table S2 Calculated energies of the 10 lowest-lying singlet and triplet excited states used to produce Figure S2.

	0F		1F		2F		3F	
**State**	**S**	**T**	**S**	**T**	**S**	**T**	**S**	**T**
1	2.814	2.085	2.886	2.154	2.836	2.119	2.825	2.113
2	3.145	2.673	3.208	2.735	2.849	2.129	2.832	2.117
3	3.427	2.843	3.380	2.809	3.190	2.720	2.857	2.135
4	3.768	2.991	3.815	3.041	3.202	2.732	3.176	2.708
5	4.172	3.388	4.174	3.410	3.300	2.785	3.178	2.709
6	4.268	3.480	4.254	3.464	3.428	2.821	3.181	2.711
7	4.471	3.697	4.371	3.693	3.785	3.017	3.307	2.797
8	4.520	3.746	4.479	3.778	3.796	3.035	3.311	2.800
9	4.551	3.940	4.517	3.879	4.148	3.402	3.472	2.840
10	4.621	3.978	4.606	3.983	4.178	3.413	3.771	3.008

Cyclic Voltammetry

The oxidation profiles for **0F**-**3F** are similar due to the common donor moieties across the series. **0F**, **1F** and **2F** show two quasi-reversible oxidation waves occurring at similar potentials. Cycling over both oxidation events in **3F** renders both as being essentially irreversible, however a shorter scan suggests the first oxidation is in fact quasi-reversible, albeit a complex wave profile is observed upon reversing the potential.

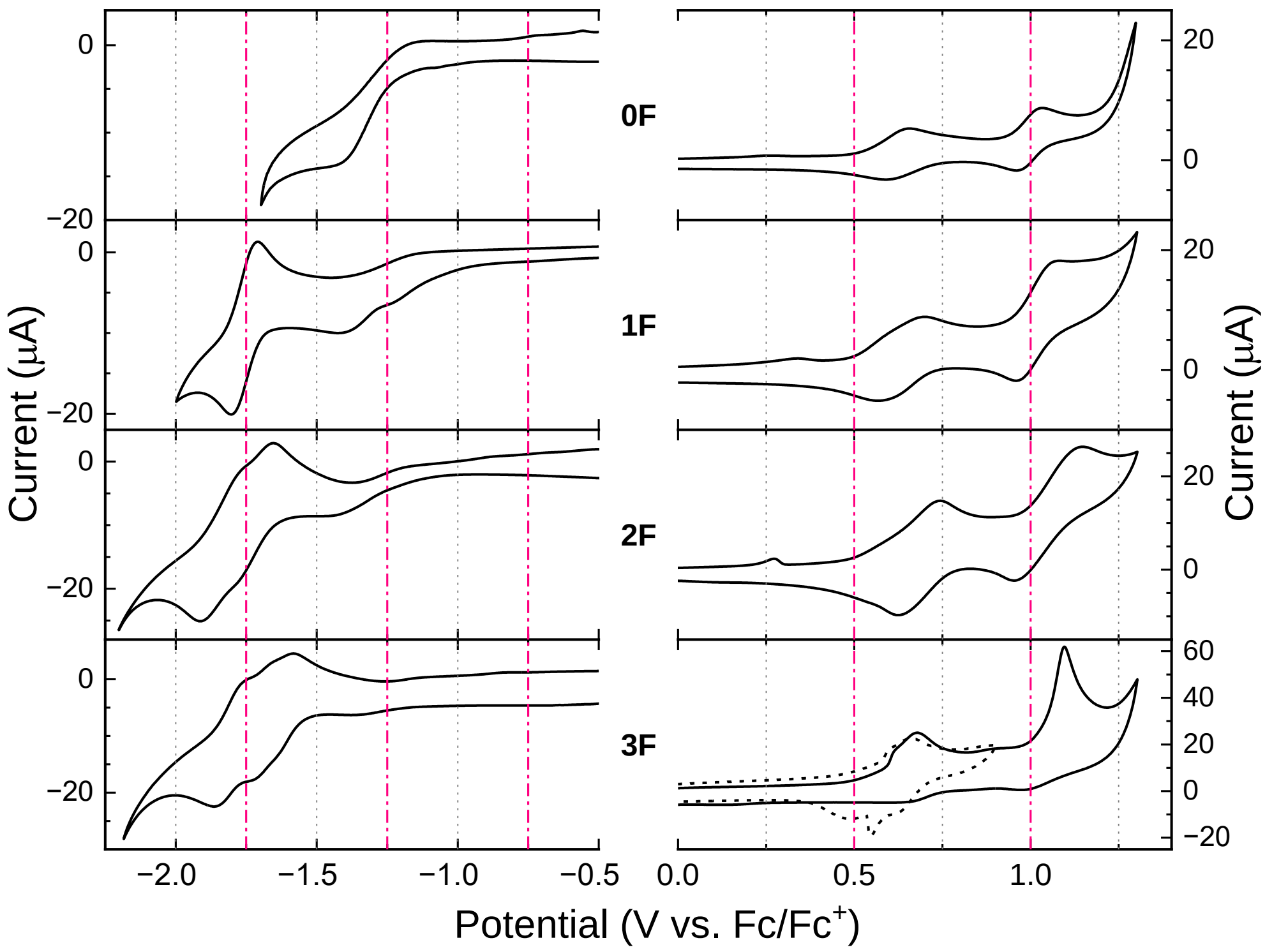

Figure S3 Cyclic voltammograms for **0F-3F**. The dashed line in the oxidation scan for **3F** highlights the improved reversibility if the second wave is not included in the scan.

Table S3 Electrochemical properties of **0F-3F**.[a]

	E_{ox1} (V)	E_{ox2} (V)	E_{red1} (V)	E_{red2} (V)	E_{red3} (V)
**0F**	+0.63[q]	+1.00[q]	−1.43[ir]		
**1F**	+0.64[q]	+1.01[q]	−1.42[ir]	−1.76[r]	
**2F**	+0.68[q]	+1.05[q]	−1.45[ir]	−1.73[q]	−1.84[q]
**3F**	+0.60[q]	+1.10[q]	−1.61[q]	−1.70[q]	−1.81[q]

a Cyclic voltammetry data were obtained from solutions of molarity 1 mM of the compound, 0.1 M [n-Bu_4N][PF_6] in CH_2Cl_2, scan rate 100 mV s−1, using a glassy carbon disc working electrode, a non-aqueous Ag/$AgNO_3$ reference electrode and a platinum wire counter electrode. Potentials are quoted versus the Fc/Fc+ couple which was used as an internal reference. Reversible and quasi-reversible reductions are quoted as E_{half} while for irreversible reductions peak potentials are provided. r = reversible, q = quasi-reversible, ir = irreversible peak.

In the cathodic regime a single irreversible reduction is observed for **0F** at −1.43 V. This differs from phenazine itself which has a fully reversible reduction at a more negative potential.[S9-S11] Fusion of further rings[S12] or iptycenes[S11,S13] onto phenazine has previously been observed to modulate the magnitude of both the reduction potential, and its reversibility to a significant level. In both scenarios, the LUMO becomes more accessible while the presence of the iptycene tends to retain the reversibility of the reduction. This first reduction persists to some extent in the voltammograms of **1F** and **2F** but becomes increasingly less pronounced which suggests that the increasing volume of the molecules is influencing the nature of its interaction with the working electrode surface. The reversibility of subsequent reductions observed for **1F**-**3F** are improved by the presence of the triptycene moiety.

The influence of the multiple ring systems is apparent in the reductions of **1F**-**3F** where the reduction scan is seen to consist of one wave in **1F** then two and three overlapping waves for **2F** and **3F** respectively. The profile of the reduction events in **3F** is comparable to those observed previously for some phenoxazine/quinoxaline-based ICT systems[S14] but occurring at much less negative potentials, due to the more strongly electron-withdrawing nature of the phenazine rings. The onset of these reductions becomes increasingly negative from **1F**-**3F** also indicative of the incrementally increasing electron-withdrawing strength of the entire tripctyene-framework upon addition of further fused di-aza heterocycles stabilizing the LUMO. A similar trend in reduction potentials was observed in the all-phenazine iptycene of Ushiroguchi et al which had a reduction potential of −1.22 V (vs. Fc/Fc^+) compared with phenazine itself at −1.56 V (vs. Fc/Fc^+) under the same experimental conditions.[S11]

Vibrational Modes Analysis

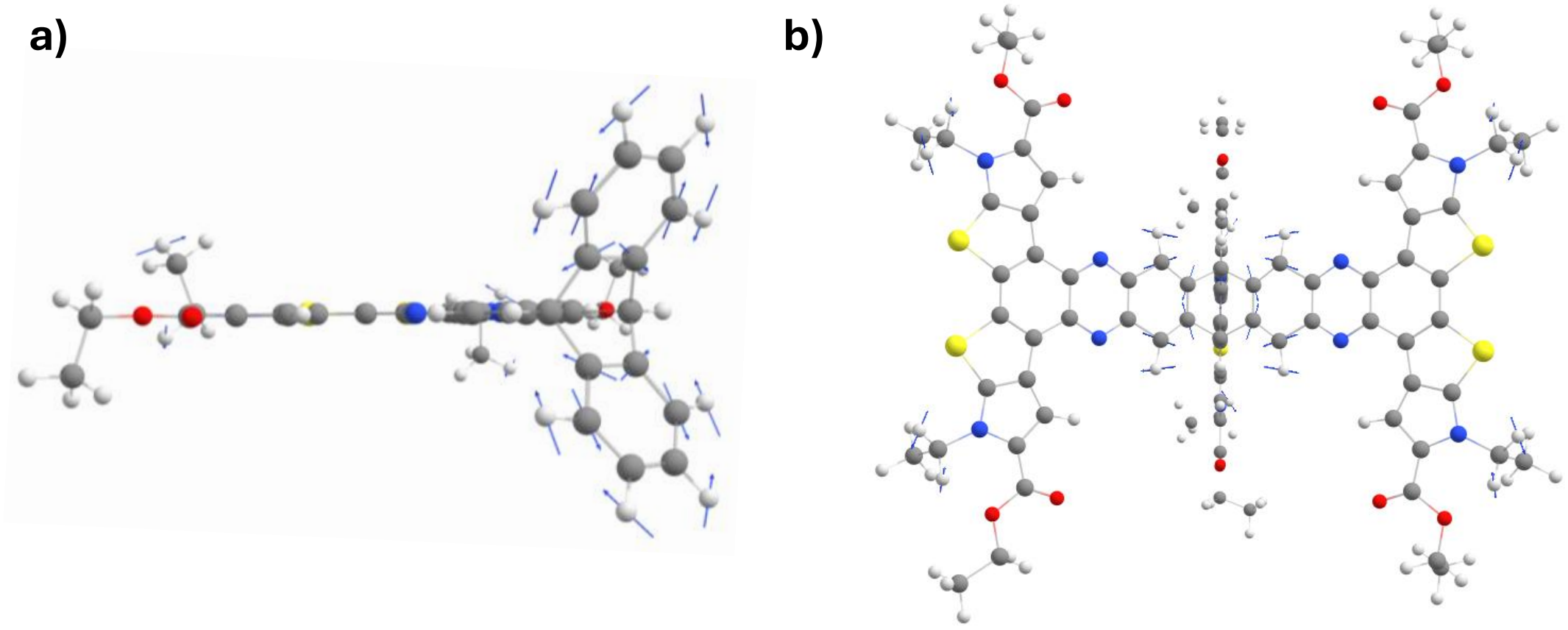

Figure S4. Calculated eigenvectors (B3LYP-6-31G(d)) for the triptycene-localised modes of (a) **1F** and (b) **3F**, calculated to occur at 1445 – 1470 cm^{-1}.

UV/Vis and Beer Lambert Plots

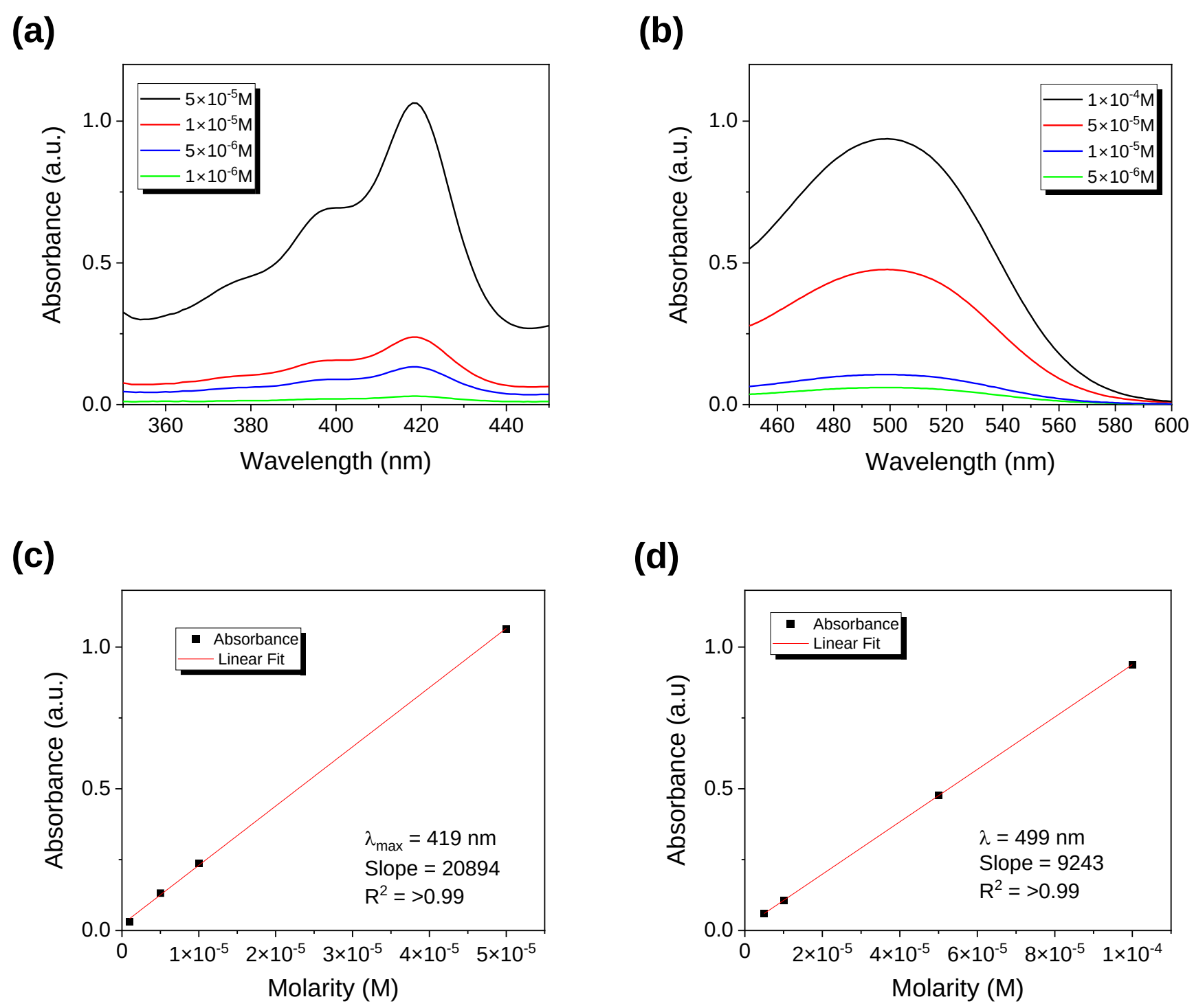

Figure S5 (a-b) UV/Vis spectra of **0F** at varying concentrations in CH_2Cl_2 using a 1 cm pathlength cuvette and Beer-Lambert plots for (c) λ_{max} = 419 nm and (d) λ_{max} = 499 nm.

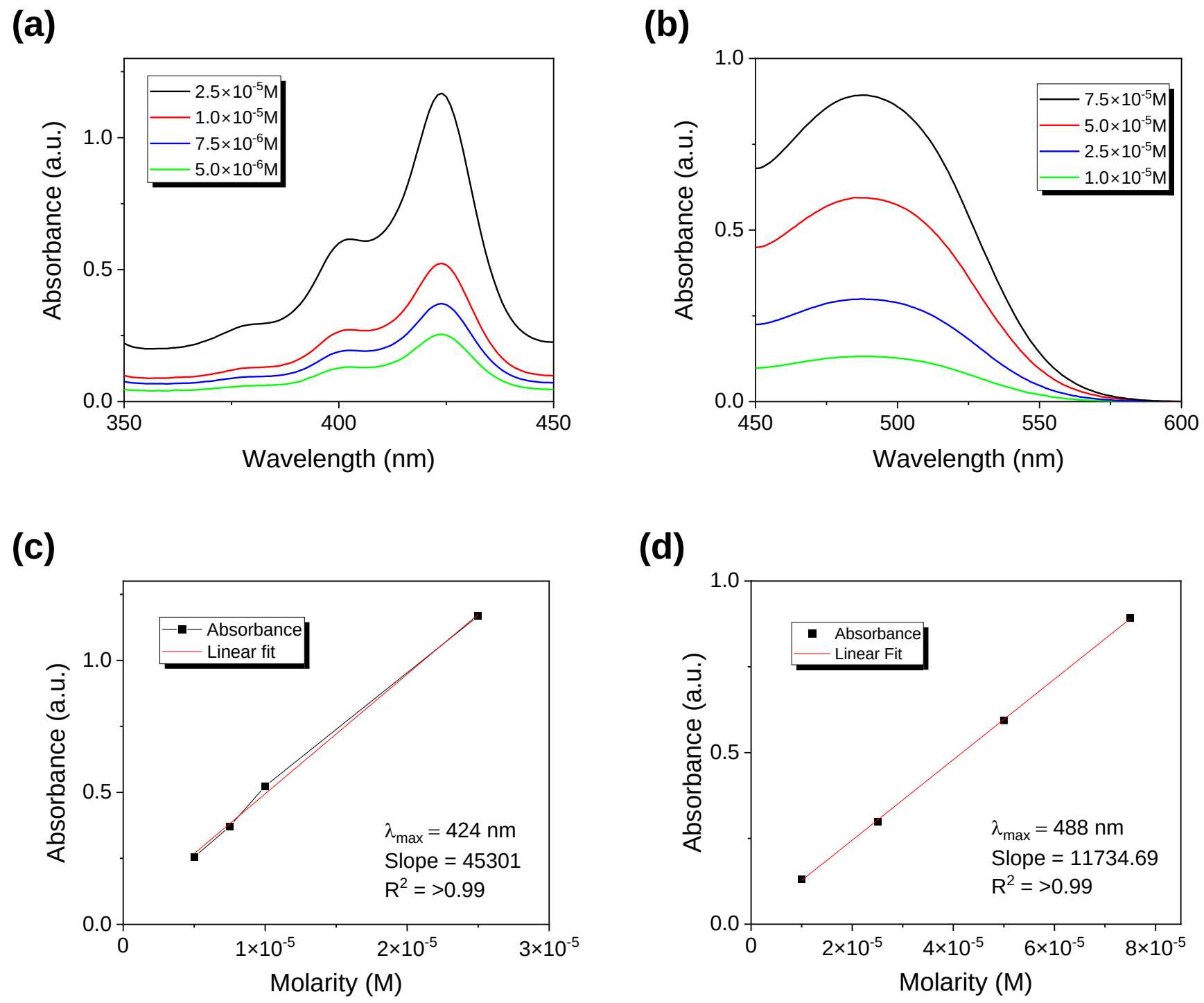

Figure S6 (a-b) UV/Vis spectra of **1F** at varying concentrations in CH_2Cl_2 using a 1 cm pathlength cuvette and Beer-Lambert plots for (c) λ_{max} = 424 nm and (d) λ_{max} = 488 nm.

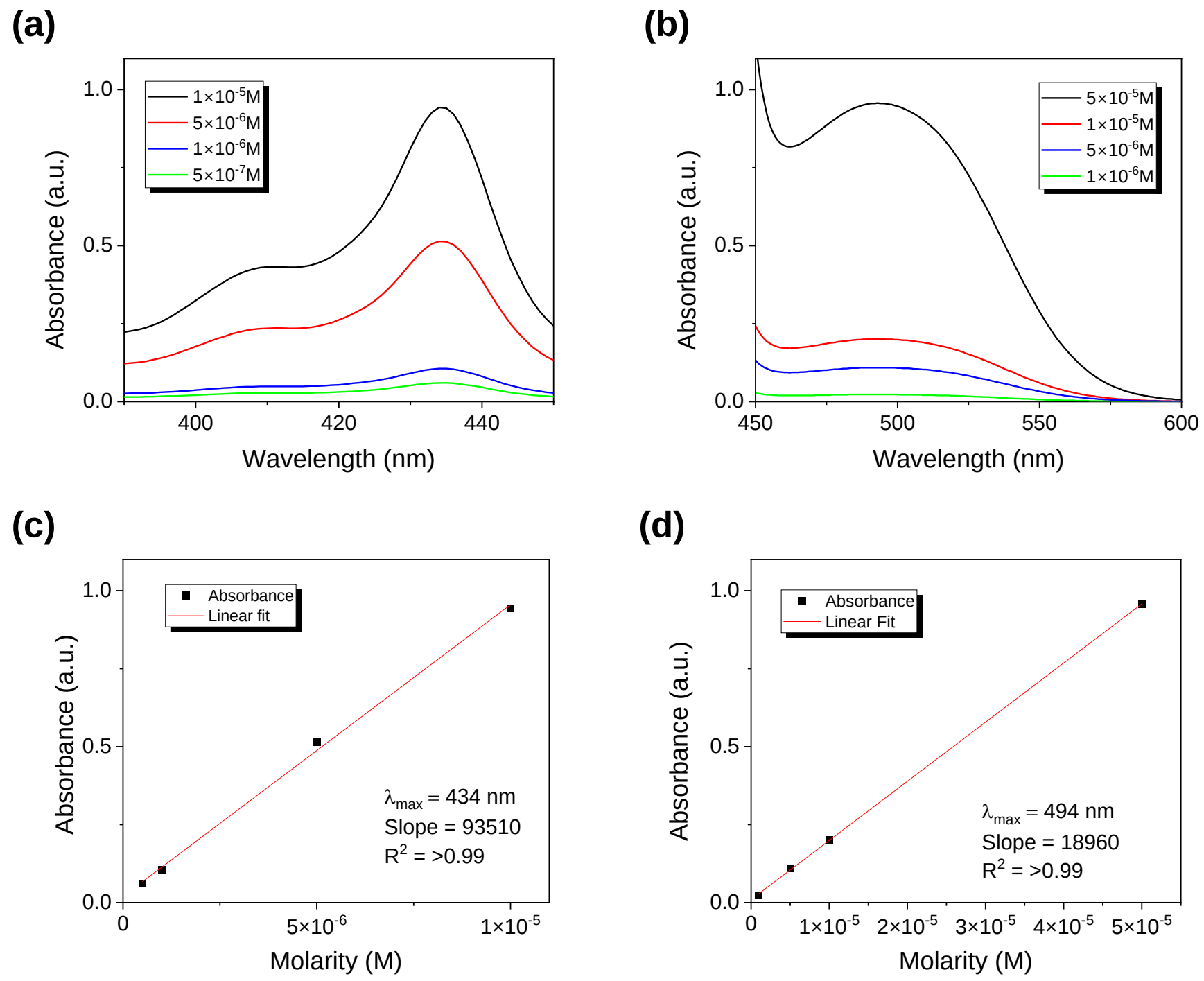

Figure S7 (a-b) UV/Vis spectra of **2F** at varying concentrations in CH_2Cl_2 using a 1 cm pathlength cuvette and Beer-Lambert plots for (c) λ_{max} = 434 nm and (d) λ_{max} = 494 nm.

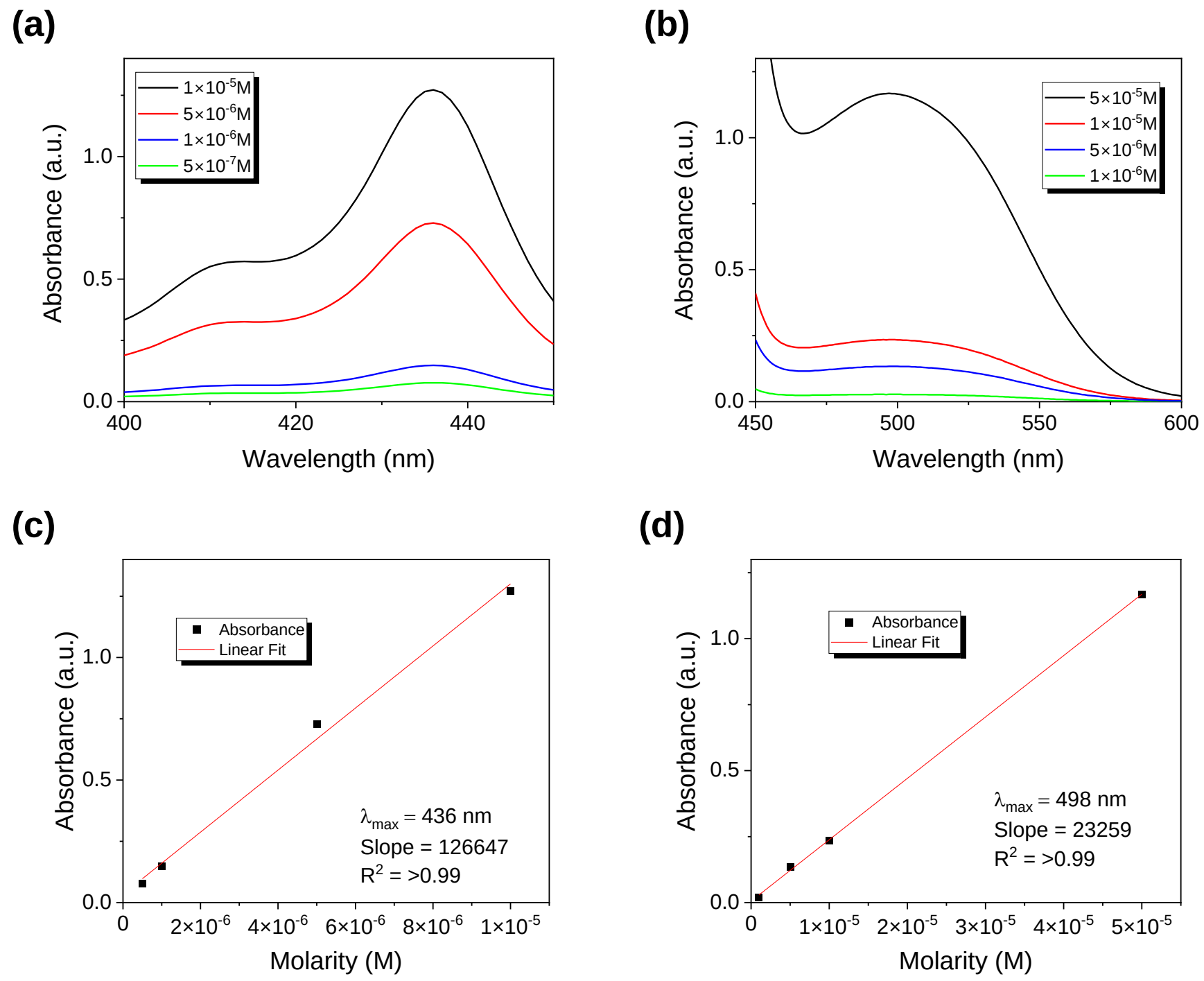

Figure S8 (a-b) UV/Vis spectra of **3F** at varying concentrations in CH_2Cl_2 using a 1 cm pathlength cuvette and Beer-Lambert plots for (c) λ_{max} = 436 nm and (d) λ_{max} = 498 nm.

Solvatochromism

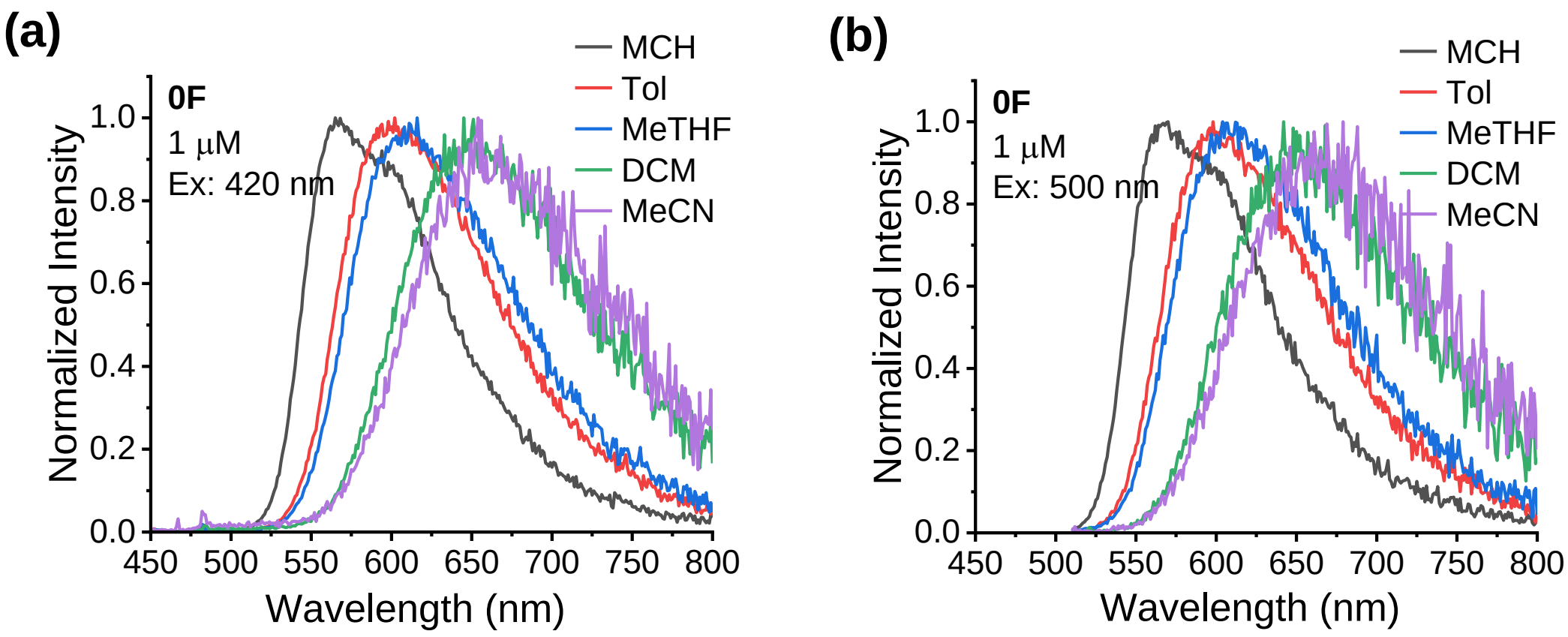

Figure S9 Normalised emission spectra in a range of solvents for **0F** using an excitation wavelength of (a) 420 nm and (b) 500 nm. Analyte molarity = 1 μM. (MCH = methylcyclohexane, Tol = toluene, MeTHF = 2-methyltetrahydrofuran, DCM = dichloromethane, MeCN = acetonitrile).

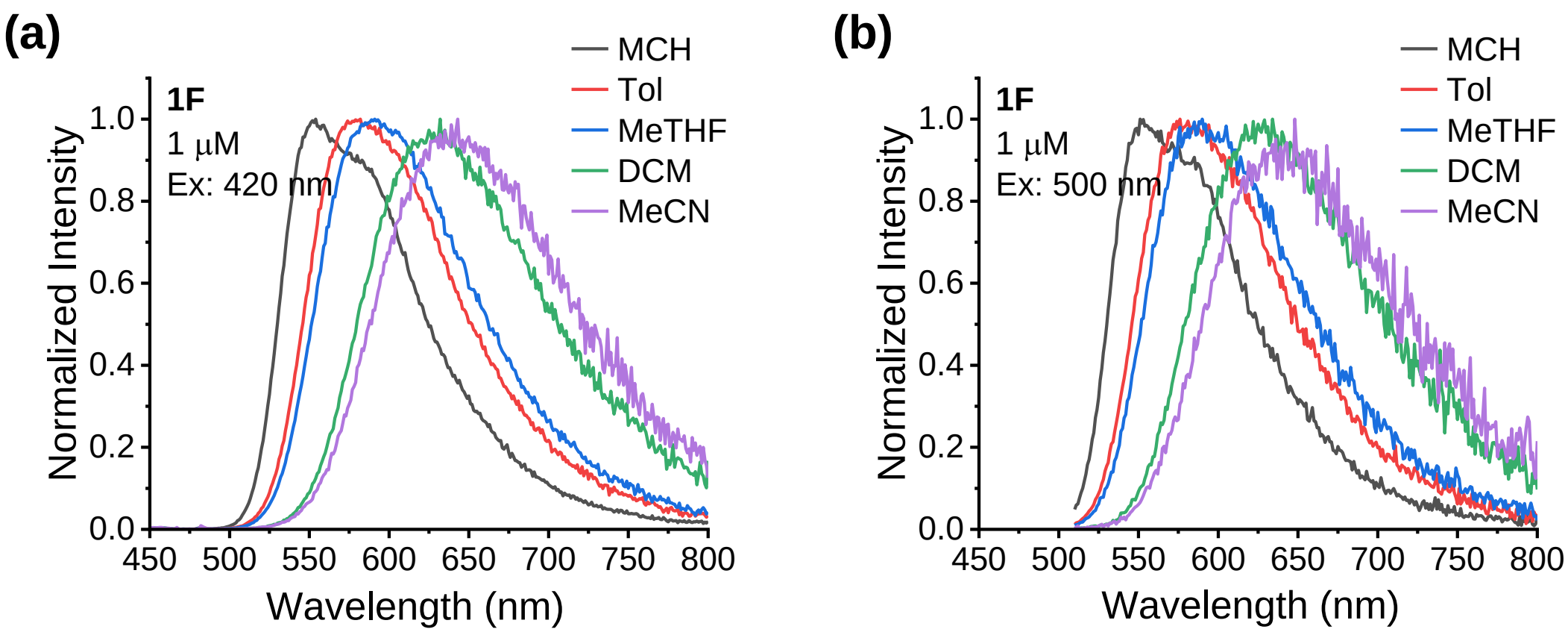

Figure S10 Normalised emission spectra in a range of solvents for **1F** using an excitation wavelength of (a) 420 nm and (b) 500 nm. Analyte molarity = 1 μM. (MCH = methylcyclohexane, Tol = toluene, MeTHF = 2-methyltetrahydrofuran, DCM = dichloromethane, MeCN = acetonitrile).

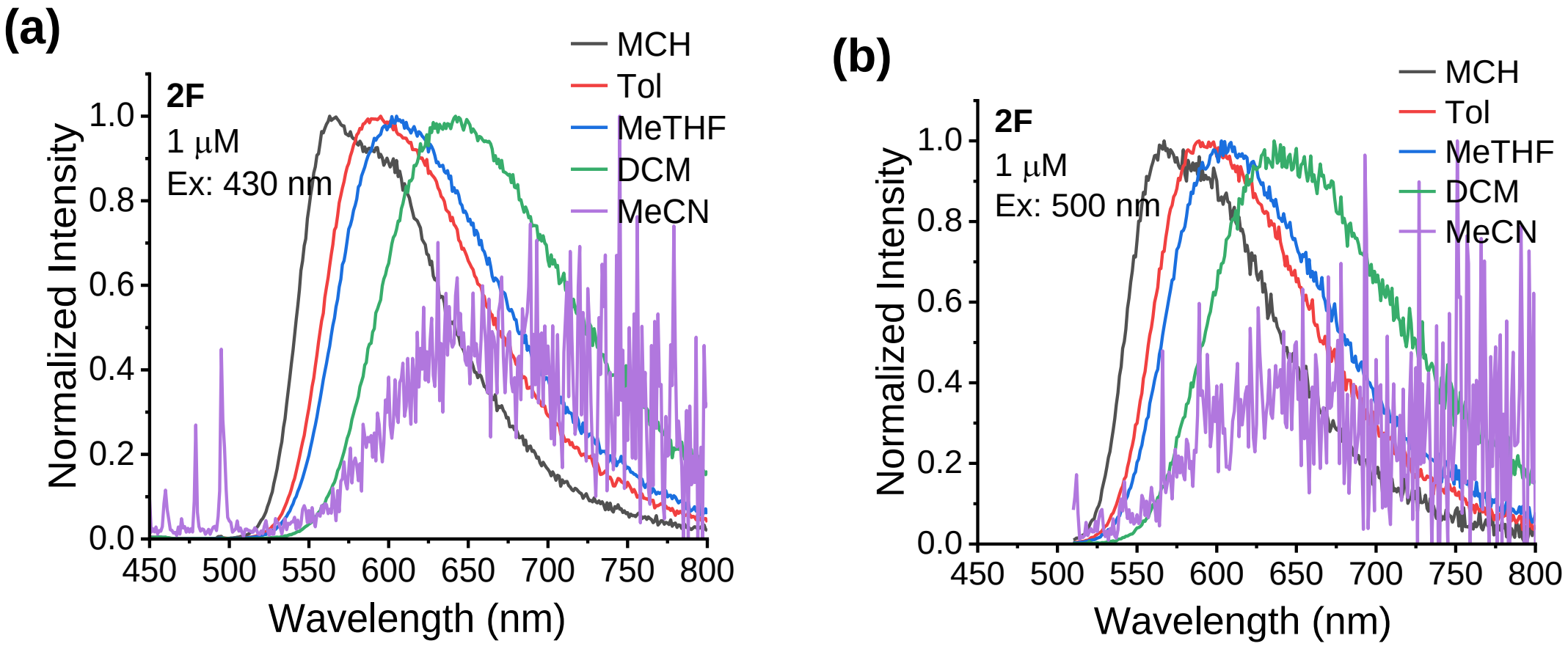

Figure S11 Normalised emission spectra in a range of solvents for **2F** using an excitation wavelength of (a) 430 nm and (b) 500 nm. Analyte molarity = 1 μM. (MCH = methylcyclohexane, Tol = toluene, MeTHF = 2-methyltetrahydrofuran, DCM = dichloromethane, MeCN = acetonitrile).

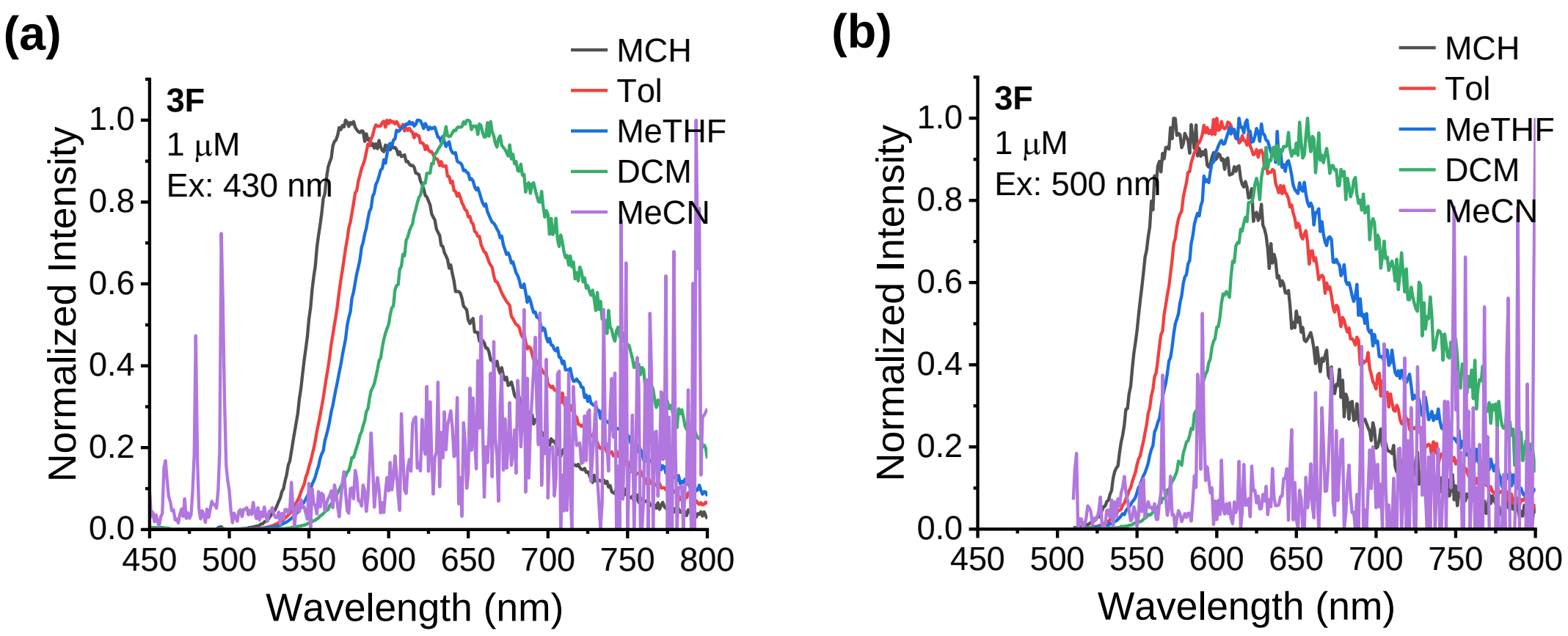

Figure S12 Normalised emission spectra in a range of solvents for **3F** using an excitation wavelength of (a) 430 nm and (b) 500 nm. Analyte molarity = 1 μM. (MCH = methylcyclohexane, Tol = toluene, MeTHF = 2-methyltetrahydrofuran, DCM = dichloromethane, MeCN = acetonitrile).

Time-Resolved Photophysics

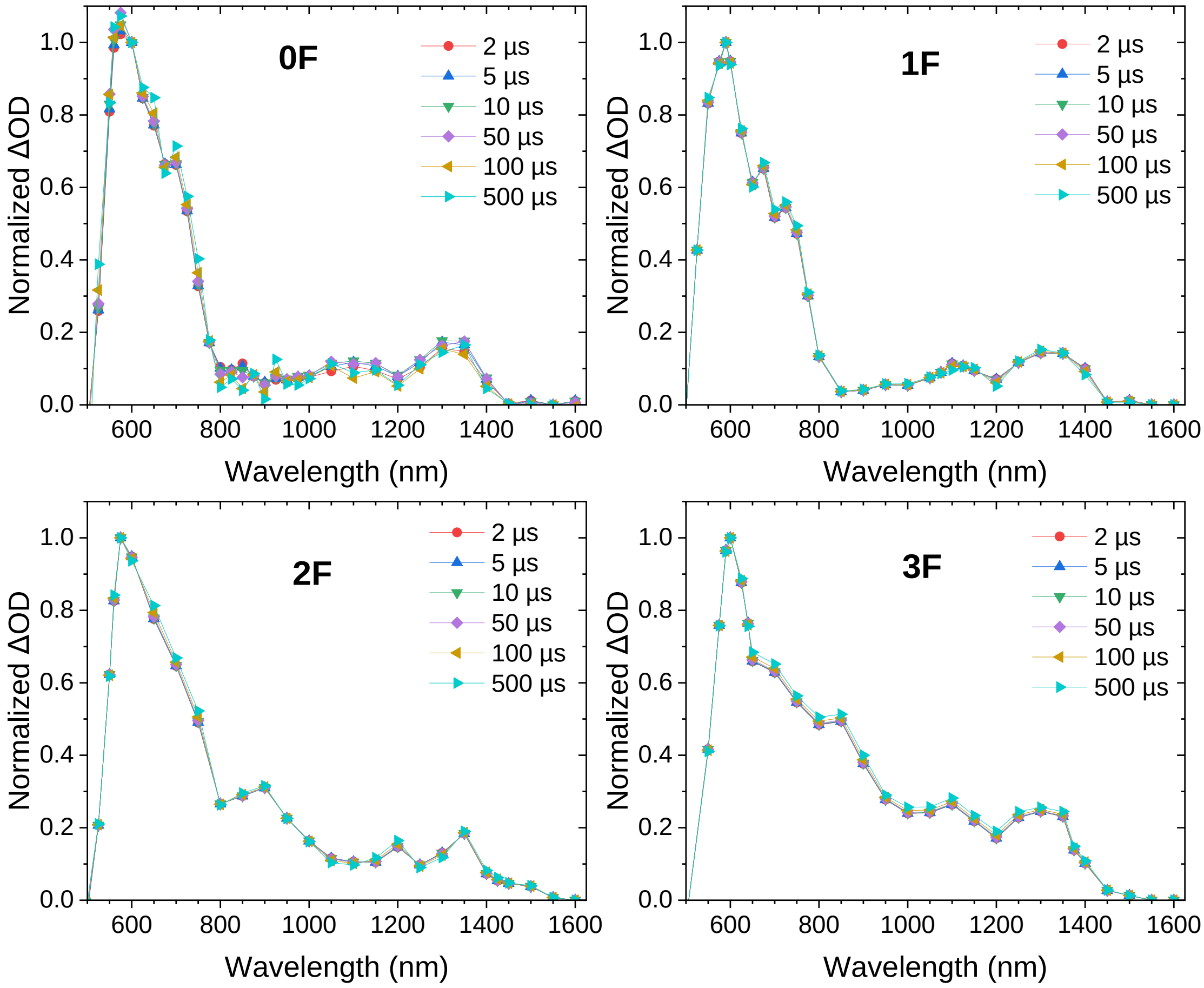

Figure S13 Normalised TA spectra of **0F-3F** (toluene) solution, excited at 415 nm, 20 µJ/cm^2.

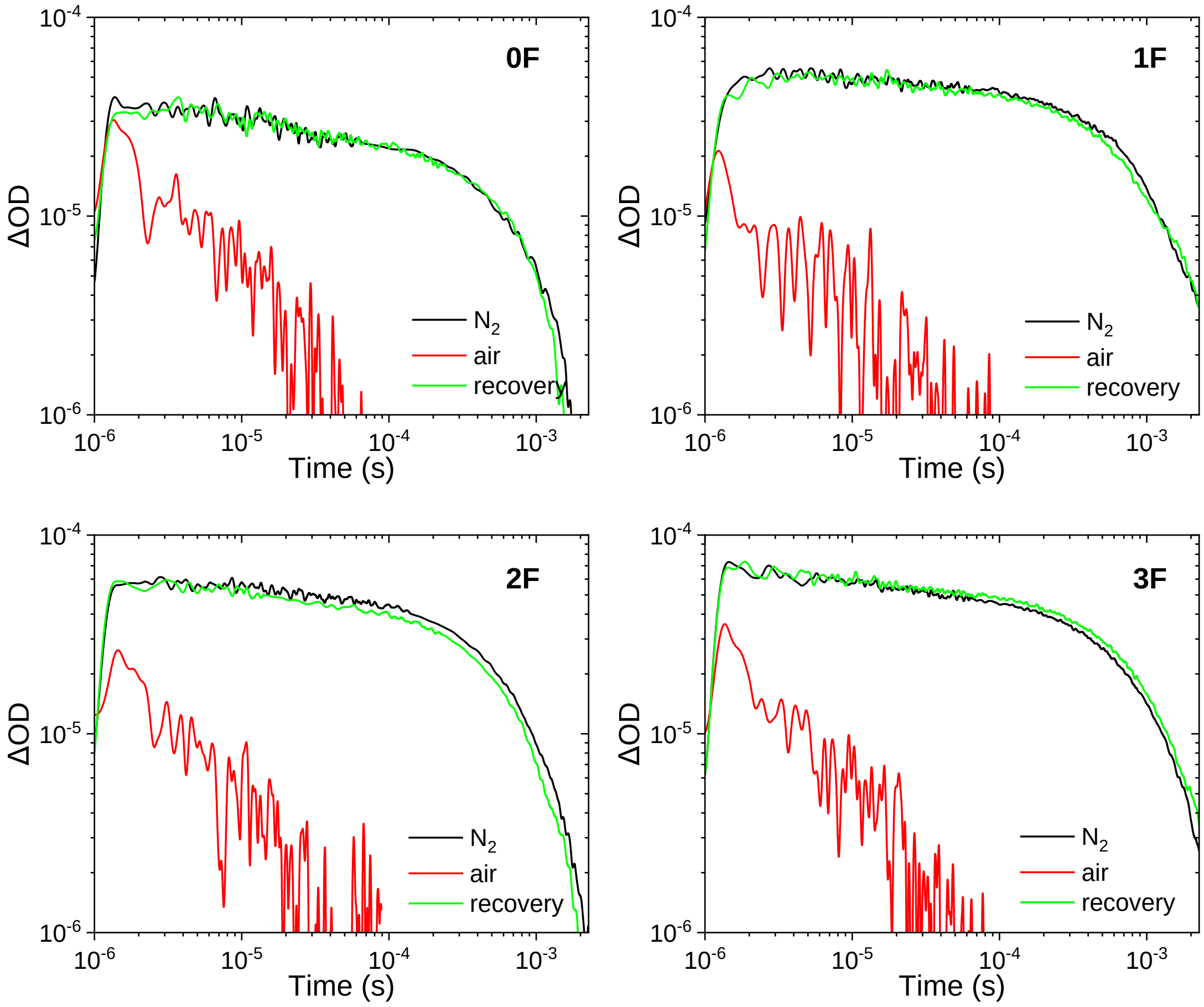

Figure S14 Reversible oxygen quenching of the long-lived decay component of **0F-3F**.

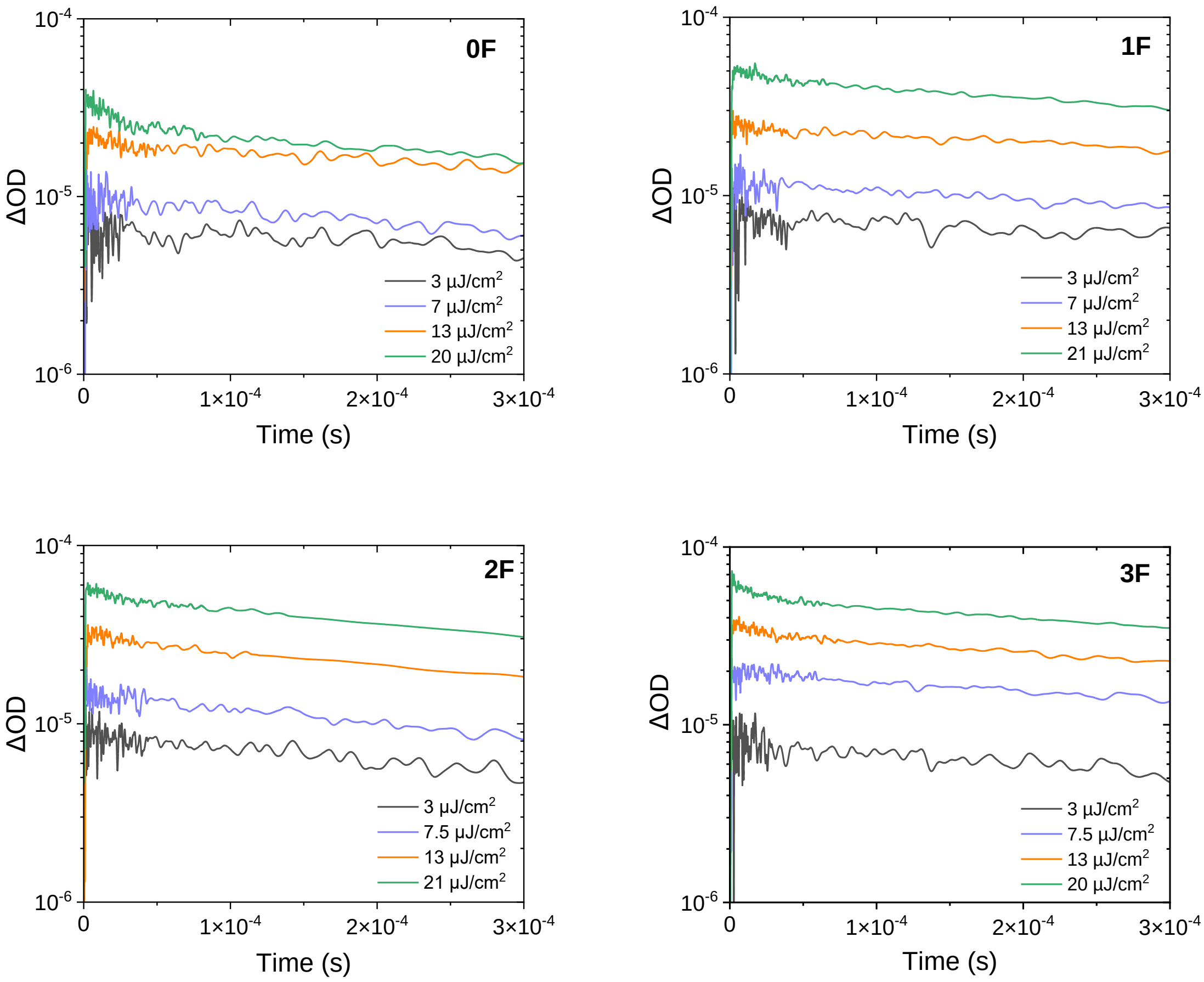

Figure S15 Energy dependence of **0F-3F** (toluene) solution, excited at 415 nm, probed at 700 nm

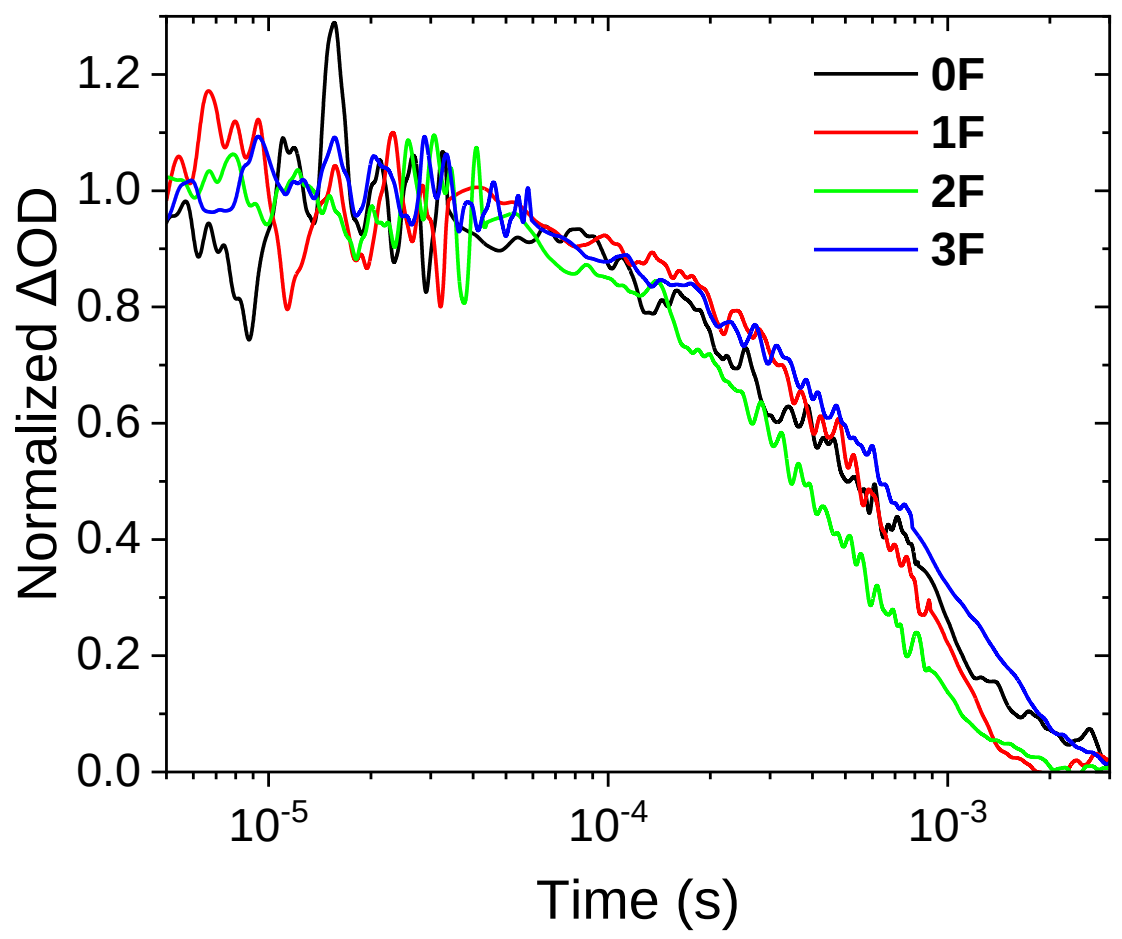

Figure S16 Comparison between the normalised triplet kinetics excited at 415 nm, 7 μJ cm^{-2}, probed at 700 nm.

References

S1. Vogt, A.; Henne, F.; Wetzel, C.; Mena-Osteritz, E.; Bäuerle, P. Synthesis and Characterization of *S*,*N*-heterotetracenes. *Beilstein J. Org. Chem.* **2020**, *16*, 2636–2644. DOI: 10.3762/bjoc.16.214

S2. Chong, J. H.; MacLachlan, M. J. Robust Non-Interpenetrating Coordination Frameworks from New Shape-Persistent Building Blocks. *Inorg. Chem.* **2006**, *45* (4), 1442–1444. DOI: 10.1021/ic052123w

S3. White, N. G.; MacLachlan M. J. Soluble Tetraaminotriptycene Precurors. *J. Org. Chem.* **2015**, *80* (16), 8390−8397. DOI: 10.1021/acs.joc.5b01221

S4. Zhu, Z.; Zhu, J.; Li, J.; Ma, X. Enhanced Gas Separation Properties of Tröger's Base Polymer Membranes Derived from Pure Triptycene Diamine Regioisomers. *Macromolecules*, **2020**, *53* (5), 1573–1584. DOI: 10.1021/acs.macromol.9b02328

S5. Zhang, C.; Chen, C.-F. Synthesis and Structure of 2,6,14- and 2,7,14-Trisubstituted Triptycene Derivatives. *J. Org. Chem.*, **2006**, *71* (17), 6626–6629. DOI: 10.1021/jo061067t

S6. Menke, E. H.; Lami, V.; Vaynzof, Y.; Mastalerz, M. π-Extended rigid triptycene trisaroylenimidazoles as electron acceptors. *Chem. Commun.* **2015**, *52* (5), 1048–1051. DOI: 10.1039/C5CC07238G

S7. Kohl, B.; Rominger, F.; Mastalerz, M. Rigid p-Extended Triptycenes via a Hexaketone Precursor. *Org. Lett.*, **2014**, *16* (3), 704–707. DOI: 10.1021/ol403383y

S8. Mastalerz, M.; Sieste, S.; Cenić, M.; Oppel, I. M. Two-Step Synthesis of Hexaammonium Triptycene: An Air-Stable Building Block for Condensation Reactions to Extended Triptycene Derivatives. *J. Org. Chem.* **2011**, *76* (15), 6389–6393. DOI: 10.1021/jo200843v

S9. Paduszek, B.; Kalinowski, M. K. Redox behaviour of phenothiazine and phenazine in organic solvents. *Electrochimica Acta* **1983**, *28* (5), 639–642. DOI: 10.1016/0013-4686(83)85057-9

S10. Sawyer, D. T.; Komai, R. Y. Electrochemistry of phenazine at a platinum electrode in aprotic solvents. *Anal. Chem.* **1972**, *44* (4), 715–721. DOI: 10.1021/ac60312a002

S11. Ushiroguchi, R.; Shuku, Y.; Suizu, R.; Awaga, K. Variable Host–Guest Charge-Transfer Interactions in 1D Channels Formed in a Molecule-Based Honeycomb Lattice of Phenazine Analogue of Triptycene. *Cryst. Growth Des.* **2020**, *20* (12), 7593–7597. DOI: 10.1021/acs.cgd.0c01176.

S12. Müller, M.; Reiss, H.; Tverskoy, O.; Rominger, F.; Freudenberg, J.; Bunz, U. H. F. Stabilization by Benzannulation: Butterfly Azaacenes. *Chem. Eur. J.* **2018**, *24* (49), 12801–12805. DOI: 10.1002/chem.201803118

S13. Biegger, P.; Stolz, S.; Intorp, S. N.; Zhang, Y.; Engelhart, J. U.; Rominger, F.; Hardcastle, K. I.; Lemmer, U.; Qian, X.; Hamburger, M.; Bunz, U. H. F. Soluble diazaiptycenes: materials for solution-processed organic electronics. *J. Org. Chem.* **2015**, *80* (1), 582–589. DOI: 10.1021/jo502564w

S14. Montanaro, S.; Pander, P.; Mistry, J.-R.; Elsegood, M. R. J.; Teat, S. J.; Bond, A. D.; Wright, I. A.; Congrave, D. G.; Etherington, M. K. Simultaneous enhancement of thermally activated delayed fluorescence and photoluminescence quantum yield via homoconjugation. *J. Mater. Chem. C* **2022**, *10* (16), 6306–6313. DOI: 10.1039/d2tc00460g.
